# Supplementary material for: Costs, consequences and value for money in non-medical prescribing: a scoping review
Source: BMJ Open. 2023 May 2;13(5):e067907. doi: 10.1136/bmjopen-2022-067907 (PMC10163523; doi:10.1136/bmjopen-2022-067907)
Supplement: Supplementary data [file bmjopen-2022-067907supp001.pdf]

Costs, consequences and value for money in non-medical prescribing: A scoping review

Supplementary Data

Table A.1 Search terms used in PubMed from 1 January 1999 to 1 January 2022

**Database**: PubMed		
**Date of search**: 14 January 2022		
**Search step**	**Search terms and queries**	**Results**
S1	"non-medical prescribing" OR "non-medical prescriber" OR "non-medical prescribers" OR "non-doctor prescribing" OR "non-doctor prescriber" OR "non-doctor prescribers" [All Fields]	216
S2	"non-medical prescribing" OR "non-medical prescriber" OR "non-medical prescribers" OR "non-doctor prescribing" OR "non-doctor prescriber" OR "non-doctor prescribers" [All Fields] Filters: from 1999/1/1 - 2022/1/1	215
S3	"medicine management" AND activities [All Fields] Filters: from 1999/1/1 - 2022/1/1	47
S4	S2 OR S3	262
S5	(outcome OR consequence OR effectiveness OR impact) AND (economic OR cost) [All Fields] Filters: from 1999/1/1 - 2022/1/1	555,862
S6	S4 AND S5	28
S7	"allied health profession" OR "allied health professions" OR "allied health professional" OR "allied health professionals" OR "non-medical profession" OR "non-medical professions" OR “non-medical professional" OR "non-medical professionals" OR nurse OR pharmacist OR dietitian OR dietician OR radiographer OR podiatrist OR physiotherapist OR podiatrist OR optometrist OR paramedic OR midwife [All Fields] Filters: from 1999/1/1 - 2022/1/1	563,041
S8	S6 AND S7	26

Table A.2 Search terms used in MEDLINE from 1 January 1999 to 1 January 2022

**Database**: MEDLINE		
**Date of search**: 14 January 2022		
**Step**	**Search terms and queries**	**Results**
S1	"non-medical prescribing" OR "non-medical prescriber" OR "non-medical prescribers" OR "non-doctor prescribing" OR "non-doctor prescriber" OR "non-doctor prescribers" OR "medicine management activities" in Anywhere	212
S2	"non-medical prescribing" OR "non-medical prescriber" OR "non-medical prescribers" OR "medicine management activities" in Anywhere Narrowed by: Entered date: 1999-01-01 to 2022-01-01	211
S3	(outcome OR consequence OR effectiveness OR impact) AND (economic	356,562

Babashahi S, *et al. BMJ Open* 2023; 13:e067907. doi: 10.1136/bmjopen-2022-067907

	OR cost) in Anywhere	
S4	S2 AND S3	26
S5	"allied health profession" OR "allied health professions" OR "allied health professional" OR "allied health professionals" OR "non-medical profession" OR "non-medical professions" OR "non-medical professional" OR "non-medical professionals" OR nurse OR pharmacist OR dietitian OR radiographer OR podiatrist OR physiotherapist OR podiatrist OR optometrist OR paramedic OR midwife in Anywhere	591,003
S6	S5 AND S4	25

Table A.3 Search terms used in Scopus from 1 January 1999 to 1 January 2022

**Database**: Scopus		
**Date of search**: 14 January 2022		
**Step**	**Search terms and queries**	**Results**
S1	ALL ("non-medical prescribing" OR "non-medical prescriber" OR "non-medical prescribers" OR "non-doctor prescribing" OR "non-doctor prescriber" OR "non-doctor prescribers") AND PUBYEAR > 1998	830
S2	ALL ("medicine management activities") AND PUBYEAR > 1998	11
S3	S1 OR S2	835
S4	ALL ((outcome OR consequence OR effectiveness OR impact) AND (economic OR cost)) AND PUBYEAR > 1998	4,456,222
S5	S3 AND S4	266
S6	ALL ("allied health profession" OR "allied health professions" OR "allied health professional" OR "allied health professionals" OR "non-medical profession" OR "non-medical professions" OR "non-medical professional" OR "non-medical professionals" OR nurse OR pharmacist OR dietitian OR radiographer OR podiatrist OR physiotherapist OR podiatrist OR optometrist OR paramedic OR midwife) AND PUBYEAR > 1998	1,091,538
S7	S5 AND S6	252

Table A.4 Search terms used in Web of Science from 1 January 1999 to 1 January 2022

**Database**: Web of Science		
**Date of search**: 14 January 2022		
**Step**	**Search terms and queries**	**Results**
S1	ALL=("non-medical prescribing" OR "non-medical prescriber" OR "non-medical prescribers" OR "non-doctor prescribing" OR "non-doctor prescriber" OR "non-doctor prescribers" OR "medicine management activities") Timespan: 1999-01-01 to 2022-01-01 (Publication date)	186
S2	ALL=(cost or economic) AND ALL=(outcome OR consequence OR impact OR effectiveness)	825,022
S3	S1 AND S2	17
S4	ALL=("allied health profession" OR "allied health professions" OR "allied health professional" OR "allied health professionals" OR "non-medical profession" OR "non-medical professions" OR "non-medical professional" OR	407,152

Babashahi S, *et al. BMJ Open* 2023; 13:e067907. doi: 10.1136/bmjopen-2022-067907

	"non-medical professionals" OR dietitian OR dietician OR radiographer OR therapist OR podiatrist OR physiotherapist OR paramedic OR optometrist OR midwife OR nurse OR pharmacist) Timespan: 1999-01-01 to 2022-01-01 (Publication date)	
S5	S3 AND S4	14

Table A.5 Search terms used in Cochrane Database of Systematic Reviews from 1 January 1999 to 1 January 2022

**Database**: Cochrane Database of Systematic Reviews		
**Date of search**: 14 January 2022		
**Step**	**Search terms and queries**	**Results**
S1	"non-medical prescribing" OR "non-medical prescriber" OR "non-medical prescribers" OR "non-doctor prescribing" OR "non-doctor prescriber" OR "medicine management activities" in All Texts	7
S2	(outcome OR effectiveness OR consequence OR impact) AND (cost or economic) in All Texts	5,675
S3	S1 AND S2	7

Costs, consequences and value for money in non-medical prescribing: A scoping review

Table B. Preferred Reporting Items for Systematic reviews and Meta-Analyses extension for Scoping Reviews (PRISMA-ScR) Checklist

SECTION	ITEM	PRISMA-ScR CHECKLIST ITEM	REPORTED ON PAGE #
**TITLE**			
Title	1	Identify the report as a scoping review.	1
**ABSTRACT**			
Structured summary	2	Provide a structured summary that includes (as applicable): background, objectives, eligibility criteria, sources of evidence, charting methods, results, and conclusions that relate to the review questions and objectives.	1
**INTRODUCTION**			
Rationale	3	Describe the rationale for the review in the context of what is already known. Explain why the review questions/objectives lend themselves to a scoping review approach.	1, 2
Objectives	4	Provide an explicit statement of the questions and objectives being addressed with reference to their key elements (e.g., population or participants, concepts, and context) or other relevant key elements used to conceptualise the review questions and/or objectives.	2, 3, Table 2
**METHODS**			
Protocol and registration	5	Indicate whether a review protocol exists; state if and where it can be accessed (e.g., a Web address); and if available, provide registration information, including the registration number.	2
Eligibility criteria	6	Specify characteristics of the sources of evidence used as eligibility criteria (e.g., years considered, language, and publication status), and provide a rationale.	2-4
Information sources*	7	Describe all information sources in the search (e.g., databases with dates of coverage and contact with authors to identify additional sources), as well as the date the most recent search was executed.	3, 4
Search	8	Present the full electronic search strategy for at least 1 database, including any limits used, such that it could be repeated.	3, Tables A.1 to A.5 in Supplementary Data
Selection of sources of evidence†	9	State the process for selecting sources of evidence (i.e., screening and eligibility) included in the scoping review.	3, 4
Data charting process‡	10	Describe the methods of charting data from the included sources of evidence (e.g., calibrated forms or forms that have been tested by the team before their use, and whether data charting was done independently or in duplicate) and any processes for obtaining and confirming data from investigators.	3, 4
Data items	11	List and define all variables for which data were sought and any assumptions and simplifications made.	2, Table 1
Critical appraisal of individual sources of evidence§	12	If done, provide a rationale for conducting a critical appraisal of included sources of evidence; describe the methods used and how this information was used in any data synthesis (if appropriate).	NA
Synthesis of results	13	Describe the methods of handling and summarising the data that were charted.	4
**RESULTS**			
Selection of sources of evidence	14	Give numbers of sources of evidence screened, assessed for eligibility, and included in the review, with reasons for exclusions at each stage, ideally using a flow diagram.	4, 5, Figure 1
Characteristics of	15	For each source of evidence, present characteristics for which data were charted and provide the citations.	8, Table 3 and Table C in

SECTION	ITEM	PRISMA-ScR CHECKLIST ITEM	REPORTED ON PAGE #
sources of evidence			Supplementary Data
Critical appraisal within sources of evidence	16	If done, present data on critical appraisal of included sources of evidence (see item 12).	NA
Results of individual sources of evidence	17	For each included source of evidence, present the relevant data that were charted that relate to the review questions and objectives.	5-8, Table 3 and Table C in Supplementary Data
Synthesis of results	18	Summarise and/or present the charting results as they relate to the review questions and objectives.	5-8, Table 3 and Table C in Supplementary Data
**DISCUSSION**			
Summary of evidence	19	Summarise the main results (including an overview of concepts, themes, and types of evidence available), link to the review questions and objectives, and consider the relevance to key groups.	8, 9
Limitations	20	Discuss the limitations of the scoping review process.	9
Conclusions	21	Provide a general interpretation of the results with respect to the review questions and objectives, as well as potential implications and/or next steps.	9, 10
**FUNDING**			
Funding	22	Describe sources of funding for the included sources of evidence, as well as sources of funding for the scoping review. Describe the role of the funders of the scoping review.	10

JBI = Joanna Briggs Institute; PRISMA-ScR = Preferred Reporting Items for Systematic reviews and Meta-Analyses extension for Scoping Reviews; NA = Not Available.
* Where *sources of evidence* (see second footnote) are compiled from, such as bibliographic databases, social media platforms, and Web sites.
† A more inclusive/heterogeneous term used to account for the different types of evidence or data sources (e.g., quantitative and/or qualitative research, expert opinion, and policy documents) that may be eligible in a scoping review as opposed to only studies. This is not to be confused with *information sources* (see first footnote).
‡ The frameworks by Arksey and O'Malley and Levac and colleagues and the JBI guidance refer to the process of data extraction in a scoping review as data charting.
§ The process of systematically examining research evidence to assess its validity, results, and relevance before using it to inform a decision. This term is used for items 12 and 19 instead of "risk of bias" (which is more applicable to systematic reviews of interventions) to include and acknowledge the various sources of evidence that may be used in a scoping review (e.g., quantitative and/or qualitative research, expert opinion, and policy document).

Reference*:* Tricco AC, Lillie E, Zarin W, O'Brien KK, Colquhoun H, Levac D, et al. PRISMA Extension for Scoping Reviews (PRISMAScR): Checklist and Explanation. Ann Intern Med. 2018;169:467–473. http://doi.org/10.7326/M18-0850.

Table C: Cost and outcome measures and key findings of included studies

Authors (year)	Type of prescribing	Outcome measures	Cost and resource use measures	Perspective	Study main findings
Black et al. (2022)[7]	Nurse IP	Patient consultation experience; patient satisfaction with information about medicines; medication appropriateness; medication effectiveness for the condition using the number of unplanned re-consultations in 3 months	From the NHS perspective: NMP course training and governance (staff supervision, study time and backfill); clinic processes (including medication provision, error, appropriateness, consultation duration, impact on the workload of other professionals, rates of unplanned re-consultations).<br>From nurses' perspective: study leave; personal time to study; out-of-pocket expenses for travel and purchase of learning resources for training; prospect promotions.	UK NHS, nurses and patients	***Consequences***<br>- Patient satisfaction: over 96% in both groups.<br>- Medication decisions safety: 96% for nurse prescribers vs 99% for PGD users.<br>- Medication errors: minor for both nurse prescribers (56%) and PGD users (62%), mainly documentation-related (78%), with no patients harmed.<br>- Consultation duration and unplanned re-consultations: similar for both groups. Nurse prescribers sought assistance from colleagues less frequently but provided longer consultations.<br>***Costs***<br>- NMP training fees: fully paid by their employers or health grants (£900 to £3,555) in 2016.<br>- Study days: An average of 20.1 employer-paid study days were reported by 92% of nurses, and an average of 7.4 clinically supervised days, for each nurse (an average cost of £6,45 to the NHS per nurse) during training. Eighty-one percent of nurse prescribers spent an average of 26.3 days of personal time studying for their NMP qualifications.<br>- PGD-related costs to the employer: £912 for creating a new PGD and £276 for updating.<br>- Medication costs: the average costs per patient were higher for the nurse prescriber group (£19) than for PGD users (£11.25).<br>- Nurse prescribers were in higher pay bands.
Carey et al. (2020)[20]	Physiotherapist IP<br>Podiatrist IP	Patient satisfaction; ease of access to services; quality of life	Rates of relevant tests or services ordered; frequency of new medicines; referrals to other health professionals; frequency of follow-up; consultations numbers and durations; time spent discussing the patient with other colleagues; unplanned consultations for the same condition within two months of the index consultation.	NA	***Consequences***<br>- Acceptability of independent prescribing: high (77%), with 23% preferring a GP to prescribe.<br>- Quality of life and patient satisfaction with services: significant improvements between baseline and follow-up with no significant differences in both groups.<br>***Costs***<br>- Consultation duration: An average of 6.8 minutes higher for physiotherapist prescribers and 3.5 minutes for podiatrist prescribers compared to non-prescribers.<br>- The prescriber podiatrists more frequently ordered medications and tests than non-prescribing colleagues. No cost was calculated and reported.<br>- Follow-up consultations: no significant differences in both groups.<br>- Cost of consultations: An average of £7.95 for physiotherapist prescribers and £8.62 for podiatrist prescribers higher than non-prescriber groups.<br>- No NMP training-related costs (training course, travel, supervision time, etc) were reported.

Babashahi S, *et al. BMJ Open* 2023; 13:e067907. doi: 10.1136/bmjopen-2022-067907

Authors (year)	Type of prescribing	Outcome measures	Cost and resource use measures	Perspective	Study main findings
Al Hamarneh et al. (2019)[37]	Pharmacist IP	QALYs; life years and CVD risk	Cost of baseline and follow-up visits, cost of half-day training and direct medical costs (e.g. inpatient, outpatient, medication, etc)	Canadian Medicare	***Consequences***<br>- Life years: increased by 0.11 per patient; QALYs: increased by 0.19; risk of CVD: decreased by 0.10.<br>***Costs***<br>- Medical costs: decreased by CA$2,149 compared to usual care.<br>- Cost of pharmacist intervention – 1st year: CA$233 [baseline visit: CA$125 (baseline); CA$25 (per follow-up) and cost of half-day training: CA$500 (CA$33.3 per patient)].<br>- Cost of pharmacist intervention – 2nd year: CA$175.<br>***Cost-effectiveness***<br>- Pharmacist prescribing was estimated to save more than CA$4.4 billion, add 576,689 QALYs and prevent more than 8.9 million CVD events over 30 years if applied to only 15% of the eligible patients in Canada.
Hale et al. (2018)[38]	Pharmacist IP	QALYs	Annual costs of existing and new pharmacy services; time taken by the pharmacist to prescribe (minus time saved for medical prescriber); annual salary of pharmacist (minus time costs offset by medical prescribers not having to prescribe); direct costs of acute DVT and PE to the healthcare system	Australian healthcare sector	***Consequences***<br>- QALYs: increased by 0.02 per patient in the pharmacist prescriber group.<br>- The proportion of treated VTE patients: 100% in the pharmacist prescriber and 91% in the GP groups.<br>***Costs***<br>- Cost of introducing the pharmacist IP: AU$2.24 per patient (calculated based on annual pharmacist salary and overhead costs minus GP time not required to prescribe).<br>***Cost-effectiveness***<br>- The probability of pharmacist prescribing and care being cost-effective at a willingness-to-pay of $AU40,000 was 95%.
Marra et al. (2017)[39]	Pharmacist IP	Reduced systolic blood pressure in patients with hypertension; relative risk of CVD; relative risk of renal disease; hazard ratio of mortality after CVD; life years; and HRQoL	Direct medical costs of the health conditions and the costs associated with implementing the pharmacist intervention strategy and training.	Canadian Medicare	***Consequences***<br>- Blood pressure and CVD cases: reduced by 0.21 per patient in the pharmacist prescribing group<br>- Life years: increased by 0.3 in the pharmacist prescribing group<br>- QALYs: increased by 0.4 in the pharmacist prescribing group<br>***Costs***<br>- Cost of pharmacist intervention – 1st year: CA$200 [baseline visit: CA$125 (baseline); CA$25 (per follow-up) and cost of half-day training: CA$500 (CA$33.3 per patient)].<br>- Cost of pharmacist intervention – 2nd year: CA$75.<br>- Cost of pharmacist intervention – 3rd year: CA$50.<br>***Cost-effectiveness***<br>- Pharmacist prescribing was found cost-effective and cost-saving (the reduction in costs associated with CVD and ESRD equated to cost-savings of CA$6,365 per patient in the pharmacist group over 30 years.

Authors (year)	Type of prescribing	Outcome measures	Cost and resource use measures	Perspective	Study main findings
i5 Health (2015)[42]	IP and SP (for a range of healthcare professions, e.g. physiotherapists, pharmacists and radiographers)<br>Community nurse prescribers	NA	Number of consultations; visits and referrals to medical prescribers avoided; lower A&E attendance and waiting times; avoided readmissions; inappropriate prescribing	NA	***Consequences***<br>- NMP practitioners provided information on patient's medications in 99% of reviews and identified inappropriate medication regimens in 50% of appointments.<br>***Costs***<br>- As shown by the i5 Health analysis of the audits for different non-medical prescriber groups in primary and secondary care in England, the main savings of NMP practices included 32% of GP appointments and 31% of follow-up appointments by a consultant.<br>- The annual value contributed by adding one new non-medical prescriber was over £270 million. The total cost-savings were predicted to be approximately £800 million in England in 2014.<br>- No NMP training-related costs (training course, travel, supervision time, etc) were reported.
Courtenay et al. (2015)[34]	Nurse IP	Patient-reported diabetes self-care activities; HbA1c test results; patients' satisfaction with activities, process and medicine management	Employment of cost; consultation cost; advice-seeking from other professionals and GP prescribing signing; prescribing costs; use of other health services	UK NHS	***Consequences***<br>- Patient satisfaction: in the nurse prescriber groups, patients were more satisfied with some specific aspects of care. However, there were no significant differences in general and overall satisfaction.<br>- Diabetes self-care activities: no significant differences were found.<br>- HbA1c test result: The HbA1c score decreased significantly in both groups over six months.<br>- Frequency of consultations: no significant differences were found.<br>***Costs***<br>- Consultation duration: longer consultations (average, 7.7 minutes) were provided by nurse prescribers for an additional cost of £6.<br>- New prescriptions and the use of healthcare services: no significant differences were reported regarding by patients in both groups.<br>- Most prescribing nurses were on a higher salary band than non-prescribing colleagues.
Neilson et al. (2015)[40]	Pharmacist IP	QALYs; chronic pain grade; anxiety and depression scale	Pharmacist training, pharmacist and GP time to deliver the intervention and provide follow-up; the number of hospitalisation days and outpatient visits); primary care visits for chronic pain (GP, nurse, healthcare assistant); telephone contacts for chronic pain; prescribed and non-prescribed OTC pain-related medications and health service resource use	UK NHS	***Consequences***<br>- QALYs: very small in both prescribing and review groups (relative to usual care, the mean differences in QALYs were 0.0069 for prescribing and 0.0097 for review groups, respectively)<br>***Costs***<br>- The average cost differences per patient were £77 for prescribing and £54 for review groups relative to usual care.<br>Overall, based on the expected value of sample information analysis the authors suggested a larger sample size (e.g. the optimal sample size was estimated at 780 for prescribing and 540 for the review group using a cost per QALY threshold of £20,000) for reliable findings.

Authors (year)	Type of prescribing	Outcome measures	Cost and resource use measures	Perspective	Study main findings
Norman et al. (2010)[41]	Nurse SP	Medication adherence; health status; adverse effects; patient satisfaction (with information, consultation, treatment, etc), patient's perception of improvement in their health problem; social functioning and impairment; depression scale	Training costs (time off work and other expenses, e.g. travel, supervision time, etc); costs of prescribing (e.g. time spent preparing for a prescribing, time taken to prescribe for a patient and number of patients prescribed for); patients' use of healthcare; cost of service per patient	Health and social care	***Consequences***<br>- Medicine adherence, health status, side effects, and satisfaction with overall care: no significant differences were reported between patients across the nurse and medical prescriber groups.<br>***Costs***<br>- Psychiatric inpatient costs were an average of £1,186 significantly higher per patient for the nurse prescriber group than those in the medical prescriber group.<br>- Total annual costs per patient: no significant differences were reported.<br>- The prescribing training course fee: £497 per patient.<br>- No additional training-related costs (travel, supervision time, etc) were reported.

Note. NMP: non-medical prescribing; PGD: patient group direction; IP: independent prescribing; SP: supplementary prescribing; CVD: cardiovascular disease; HRQoL: health-related quality of life; NHS: National Health Service; HbA1c: this refers to average blood sugar test. OTC: over the counter; QALYs: quality-adjusted life years. QALY is a generic metric used to value and quantify health outcomes in terms of both the quality and the quantity of life lived.[26]; ESRD: end-stage renal disease; VTE: venous thromboembolism, which manifests as either deep vein thrombosis (DVT) or pulmonary embolism (PE); GP: general practitioners; NA: not available.

Babashahi S, *et al. BMJ Open* 2023; 13:e067907. doi: 10.1136/bmjopen-2022-067907
