# Supplementary material for: Costs, consequences and value for money in non-medical prescribing: a scoping review
Source: BMJ Open. 2023 May 2;13(5):e067907. doi: 10.1136/bmjopen-2022-067907 (PMC10163523; doi:10.1136/bmjopen-2022-067907)
Supplement: Author's manuscript [file bmjopen-2022-067907.draft_revisions.pdf]

BMJ Open is committed to open peer review. As part of this commitment we make the peer review history of every article we publish publicly available.

When an article is published we post the peer reviewers' comments and the authors' responses online. We also post the versions of the paper that were used during peer review. These are the versions that the peer review comments apply to.

The versions of the paper that follow are the versions that were submitted during the peer review process. They are not the versions of record or the final published versions. They should not be cited or distributed as the published version of this manuscript.

BMJ Open is an open access journal and the full, final, typeset and author-corrected version of record of the manuscript is available on our site with no access controls, subscription charges or pay-per-view fees (http://bmjopen.bmj.com).

If you have any questions on BMJ Open's open peer review process please email info.bmjopen@bmj.com

BMJ Open

Costs, consequences and value for money in non-medical prescribing: A scoping review

Journal:	*BMJ Open*
Manuscript ID	bmjopen-2022-067907
Article Type:	Original research
Date Submitted by the Author:	31-Aug-2022

Complete List of Authors:	Babashahi, Saeideh; University of Sussex, Global Health and Infection<br>Carey, Nicola; University of the Highlands and Islands, Department of Nursing and Midwifery<br>Jani, Yogini; Imperial College London, Pharmacy Department<br>Hart, Kath; University of Surrey, Department of Nutritional Sciences<br>Hounsome, Natalia; Brighton and Sussex Medical School, Centre for Global Health Research

Keywords:	Health economics < HEALTH SERVICES ADMINISTRATION & MANAGEMENT, Health policy < HEALTH SERVICES ADMINISTRATION & MANAGEMENT, PUBLIC HEALTH

SCHOLARONE™
Manuscripts

I, the Submitting Author has the right to grant and does grant on behalf of all authors of the Work (as defined in the below author licence), an exclusive licence and/or a non-exclusive licence for contributions from authors who are: i) UK Crown employees; ii) where BMJ has agreed a CC-BY licence shall apply, and/or iii) in accordance with the terms applicable for US Federal Government officers or employees acting as part of their official duties; on a worldwide, perpetual, irrevocable, royalty-free basis to BMJ Publishing Group Ltd ("BMJ") its licensees and where the relevant Journal is co-owned by BMJ to the co-owners of the Journal, to publish the Work in this journal and any other BMJ products and to exploit all rights, as set out in our licence.

The Submitting Author accepts and understands that any supply made under these terms is made by BMJ to the Submitting Author unless you are acting as an employee on behalf of your employer or a postgraduate student of an affiliated institution which is paying any applicable article publishing charge ("APC") for Open Access articles. Where the Submitting Author wishes to make the Work available on an Open Access basis (and intends to pay the relevant APC), the terms of reuse of such Open Access shall be governed by a Creative Commons licence – details of these licences and which Creative Commons licence will apply to this Work are set out in our licence referred to above.

Other than as permitted in any relevant BMJ Author's Self Archiving Policies, I confirm this Work has not been accepted for publication elsewhere, is not being considered for publication elsewhere and does not duplicate material already published. I confirm all authors consent to publication of this Work and authorise the granting of this licence.

For peer review only

Costs, consequences and value for money in non-medical prescribing: A scoping review

Saeideh Babashahi[1,*], Nicola Carey[2], Yogini Jani[3], Kathryn Hart[4], Natalia Hounsome[5]

[1] Global Health and Infection, Brighton and Sussex Medical School, University of Sussex, Brighton, UK. Email: s.babashahi@bsms.ac.uk

[2] Department of Nursing & Midwifery, University of the Highlands and Islands, UK. Email: nicola.carey@uhi.ac.uk

[3] Centre for Medicines Optimisation Research and Education, UCLH NHS Foundation Trust and UCL School of Pharmacy, UK. Email: y.jani@ucl.ac.uk

[4] School of Biosciences & Medicine, Faculty of Health & Medical Sciences, University of Surrey Guildford, UK. Email: k.hart@surrey.ac.uk

[5] Global Health and Infection, Brighton and Sussex Medical School, University of Sussex, Brighton, UK. Email: n.ivashikina@bsms.ac.uk

* Correspondence: Saeideh Babashahi, Global Health and Infection, Brighton and Sussex Medical School, University of Sussex, Brighton, UK. Email: s.babashahi@bsms.ac.uk

Word count: 3797

For peer review only

ABSTRACT

Objectives: Non-medical prescribing (NMP) is a key feature of the UK healthcare system that refers to the legal prescribing rights granted to nurses, pharmacists and allied health professionals who have completed an approved training programme. NMP is deemed to facilitate better patient care and timely access to medicine. The aim of this scoping review is to identify, synthesise and report the evidence on the costs, consequences and value for money of NMP provided by non-medical healthcare professionals.

Methods: The scoping review was conducted using Arksey and O'Malley's refined framework. MEDLINE, Cochrane Library, Scopus, PubMed, ISI Web of Science and Google Scholar were systematically searched from 1999 to 2021, including both peer-reviewed and grey literature written in English. The research was limited to original studies evaluating economic values or both consequences and costs of NMP. The identified studies were screened independently by two reviewers for final inclusion. The results were reported in tabular form and descriptively.

Results: A total of 420 records were identified. Of these, nine studies evaluating and comparing NMP with patient group discussions, GP-led usual care, or services provided by non-prescribing colleagues were included. All studies evaluated the costs and economic values of prescribing services by non-medical prescribers, and eight assessed patient, health or clinical outcomes. Three studies showed pharmacist prescribing was superior in all outcomes and cost-saving at large scale. Others reported similar results in most health and patient outcomes across other non-medical prescribers and control groups. NMP was deemed resource-intensive to both providers and other groups of non-medical prescribers (e.g. nurses, physiotherapists, podiatrists).

Conclusions: The review demonstrated the need for quality evidence from more rigorous methodological studies examining all relevant costs and consequences to show value for money in NMP and inform the commissioning of NMP for different groups of healthcare professionals.

[Word count: 296]

Keywords: Non-medical prescribing (NMP), non-medical prescribers, health and non-health outcomes, economic impacts, cost-effectiveness, consequences, value for money

For peer review only - http://bmjopen.bmj.com/site/about/guidelines.xhtml

Strengths and limitations of this study

1) This scoping review addresses an under-researched area to provide state-of-the-art evidence on resource use and consequences (e.g. service improvement, patient satisfaction, waiting times, safety, etc) of non-medical prescribing (NMP) from a large body of peer-reviewed and grey literature.

2) The review was limited to original studies that evaluated economic impacts or both costs and consequences of NMP.

3) Drawing conclusions on the cost-effectiveness and value for money in NMP remains difficult as the existing literature is heterogenous with significant variation in participants, NMP types, comparators, study designs, costs and consequences evaluated.

For peer review only

For peer review only - http://bmjopen.bmj.com/site/about/guidelines.xhtml

INTRODUCTION

Doctors have traditionally been authorised as the main group of healthcare professionals to prescribe medicines.[1,2] With an increasing pace of population ageing and higher risks of chronic diseases, there is a growing demand for healthcare services and access to medicines.[3-5] Due, in part, to shortages within the medical workforce,[1,6] the authority for other healthcare professionals, such as nurses and pharmacists, to prescribe medicines has been introduced in several countries such as the United States, United Kingdom (UK), Canada and Australia.[7-9]

Non-medical prescribing (NMP) is a key feature of the UK healthcare system that refers to the legal prescribing rights granted to nurses, pharmacists and allied health professionals (AHPs) who have completed an approved programme of education.[27-31], delivered via a variety of methods (often hybrid), including classroom teaching, one-to-one instruction, self-directed learning and e-learning.[32] NMP first emerged in the UK in 1999 for district nurses and health visitors,[2] and it came into effect for all registered nurses in 2001 and for pharmacists in 2002.[9-11] The UK has pioneered the gradual expansion of these prescribing roles to include a wider population of healthcare professionals across both primary care and secondary care.[8,12] Since 2005 physiotherapists, podiatrists, and both diagnostic and therapeutic radiographers have been able to train to become supplementary prescribers.[13] Independent prescribing rights were subsequently granted to optometrists in 2008 optometrists[14] and in 2013 physiotherapists, podiatrists and chiropodists.[13,15,16] More recent changes in 2016, enabled therapeutic radiographers train as independent prescribers,[17] and dietitians as supplementary prescribers,[17] and in 2019 paramedics were awarded both IP and SP rights (See Table 1 for a glossary of terms).

For peer review only - http://bmjopen.bmj.com/site/about/guidelines.xhtml

Table 1. Definition of main terms (and variables) used in the study

Terminology	Definition
NMP	NMP is a term widely used in the UK, and it represents the prescribing authorities given to non-medical healthcare professionals (e.g. nurses, dietitians, physiotherapists) after completing a prescribing training course.[9,32,36]
IP	Those using IP are responsible for assessing patients' health conditions and making decisions about patients' treatment and clinical management, including prescribing, within their scope of practice.[9,32,36]
SP	Using SP, the initial assessment and diagnosis of a patient's condition are carried out by an independent prescriber (i.e. a GP or dentist), and the clinical condition is managed using a patient-specific clinical management plan agreed by the independent prescriber, supplementary prescriber and patient.[9,32,36]
PGD	PGD is a legal written framework that allows registered healthcare professionals to supply and/or prescribe specified medicines to a pre-defined group of patients without them having to see a medical prescriber (e.g. a GP).[7,37]
Medicines management or prescribing activities	A system of processes that determines how medicines are used by patients and health providers. For the purposes of this study, medicine management and prescribing activities refer to prescribing and/or the process of giving advice about medicines and the supply of medicines, as described in the research questions sub-section.[12,15]
Cost and resource use	This refers to the direct and/or indirect medical and/or non-medical resources consumed by the study population and/or the costs associated with setting up and implementing the intervention(s) under study.[38]
Consequence	This refers to the health, non-health, clinical and patient outcomes representing the effects of the intervention(s) under study.[38]
Perspective	This refers to the stakeholders' viewpoint from which economic evaluation or cost analysis is conducted.[38]
Comparator	This refers to the alternative courses of action (e.g. usual care) against which the intervention under study (e.g. NMP, the subject of this study) is evaluated.[38]

Note. NMP: non-medical prescribing; IP: independent prescribing; SP: supplementary prescribing; PGD: patient group direction; GP: general practitioner

Reviews of NMP developments and its benefits in the UK and other countries have been reported by others.[19-27] Although NMP is embedded within UK healthcare delivery in primary and secondary care, there is still a lack of evidence regarding its value for money.[10,15,38-42] Building on an earlier review by Noblet et al. [2018] on the effectiveness and cost-effectiveness of NMP from three randomised controlled trials (RCTs),[39] this scoping review aimed to assess a wider body of literature, including both peer-reviewed and grey literature, to identify state-of-the-art evidence on resource use, cost and consequence outcomes of non-medical prescribing.

For peer review only - http://bmjopen.bmj.com/site/about/guidelines.xhtml
For peer review only

METHODS

The scoping review protocol was registered with the Open Science Framework Registry on 31 July 2021 (registered DOI: 10.17605/OSF.IO/PSR3N, accessible from https://osf.io/psr3n). We followed the Preferred Reporting Items for Systematic Reviews and Meta-Analysis extension for Scoping Reviews (PRISMA-ScR) reporting guideline recommended by Tricco et al. [2018] to report our scoping review study.[46] This scoping review was conducted using the 5 stage methodological framework developed by Arksey and O'Malley and further developed by Levac et al. and the Joanna Briggs Institute to ensure rigour in reporting the review and its methodology.[43-45] The five stages are outlined below:

Stage 1: identifying the research questions

1) What types of prescribing practices (e.g. SP, IP) have been implemented and evaluated across eligible groups of healthcare professions (e.g. pharmacists, podiatrists, dietitians, etc) in different studies?

2) What measures and tools have been used to evaluate the economic values, safety, effectiveness and other consequences of prescribing by non-medical prescribers in various settings?

3) What are relevant costs, resource use, health, non-health and clinical outcomes associated with services provided by non-medical prescribers in both peer-reviewed and grey literature?

Stage 2: search strategy and screening

The scope and practice of NMPs vary globally, ranging from a restricted formulary to writing a prescription.[7,12,47] For the purposes of this review, NMP was assumed to include medicine management activities that are legally and technically considered prescribing and provided by healthcare professionals who are eligible to prescribe and have completed an approved

For peer review only - http://bmjopen.bmj.com/site/about/guidelines.xhtml

programme of education. Consistent with Courtenay et al. [2012] and Carey et al. [2020], these medicine management activities include "making recommendations for patients to buy medicine(s) over the counter; amending prescribed medication; medication review; written recommendation to GP; recommending in patients' hospital notes; prescribing via hospital medication charts; patient group directions; remote prescribing via telephone, email and fax; issuing hospital-specific prescription; signing issued prescription via GP repeat prescribing system; issuing private prescription directly to the patient".[15,51]

A comprehensive search strategy was developed by the research team to enable a stepwise search process. Based on initial exploratory research, we included grey literature and journal and conference articles with full-text written in English from 1999 to 2021.[36] On 14 January 2022, we searched PubMed, Cochrane Database of Systematic Reviews, Web of Science, Scopus and MEDLINE databases for articles published between 1 January 1999 and 1 January 2022.

The detailed search terms and strategies for different databases are presented in Tables A.1 to A.5 in Supplementary Data. A non-systematic search in Google Scholar was performed to find the grey literature. The search terms used for Google Scholar are equivalent to those of other search engines. In brief, our search strategy included (non-medical prescrib* OR NMP OR non-doctor prescrib*) AND (pharmac* OR nurs* OR non-medical healthcare professionals OR allied health professionals OR AHPs OR diet* OR radiograph* OR midwiv* OR physiotherap* OR podiatr* OR optometr* OR paramedic*) AND (consequences OR health outcomes OR non-health OR clinical outcomes OR effectiveness OR patient outcomes) AND (economic impacts OR costs OR resource use).

The scoping review included original research, RCT studies and grey literature analyses of resource use or both effects and costs to evaluate NMP provided by non-medical healthcare professionals. Commentaries, letters, protocols and editorials were excluded. A broad search strategy was implemented to ensure that the inclusion of studies was as comprehensive as

For peer review only - http://bmjopen.bmj.com/site/about/guidelines.xhtml

possible. Search terms were derived from titles, abstracts, and keywords identified in key publications and from search terms used in previous reviews related to non-medical prescribing.[8,9,39]

All articles identified from the searches were transferred to the EndNote reference manager software version 20.2©, and all duplicates and titles in other languages were removed. The PICOS (population, intervention, context, outcome, and study design) framework was used to establish eligibility criteria.[48] Table 2 provides further information regarding the inclusion criteria according to the PICOS approach.

Table 2. PICOS table describing inclusion criteria

Component	Description
Population	- Human participants (nurse, pharmacist and AHP prescribers, and patients with any health conditions managed by these groups)<br>- No restriction on age or gender
Intervention	- All types of non-medical prescribing (any medicine management activity that is legally and technically considered prescribing and provided by non-medical professionals)
Context	- All peer-reviewed published articles (in journals and conferences) and grey literature with full-text written in English from 1999 to 2021<br>- No restriction on setting or country
Outcome	- Cost and consequence outcomes of NMP services managed by nurses, pharmacists and AHPs
Study design	- Original research and clinical trials that evaluated costs and economic impacts of NMP or both cost and consequence outcomes of NMP (peer-reviewed or grey literature)

Note. PICOS: (population, intervention, context, outcome, and study design); AHP: allied health professionals; NMP: non-medical prescribing.

Stage 3: study selection

The review process included an initial screening of the title and abstract of the studies by three authors (SB, NH and NC) to assess their eligibility for full-text retrieval. Any studies that were not excluded confidently through title and abstract screening during the initial screening step were included for full-text screening. The full-text screening of the selected studies was carried

For peer review only - http://bmjopen.bmj.com/site/about/guidelines.xhtml

out independently by two reviewers from the team (SB, NH, KH and YJ). Any disagreement on selected papers was resolved through discussion among the authors. After identifying and removing duplicates, studies were excluded if (1) they were not original studies, (2) no abstract or full-text was available, (3) they were not in English, or (4) the focus of the study was outside the scope of our review (see Table 2), or (5) prescribing and medicine management activities evaluated did not meet those indicated by Courtenay et al. [2012] and Carey et al. [2020].[15,51]

Stage 4: data extraction and analysis

Data from the articles and grey literature based on the inclusion criteria mentioned above was extracted using a bespoke data extraction form. A Microsoft Excel 2019© based form was initially developed by the first author and validated by other authors for charting the data from selected studies and reporting the variables regarding the study, participants, interventions and outcome characteristics – e.g. authors, publication year, study context and design, sample size, type of prescribing, cost and consequence outcomes measures and key findings – based on our research questions (Table 1 represents the definition of the main variables). Data extracted were checked by a second reviewer for accuracy and completeness.

Stage 5: collating and reporting the results

The PRISMA-ScR reporting checklist (Table B) was used to synthesise and report the results of our scoping review.[46] Data synthesis was undertaken by the first author in consultation with the research team. The findings of selected studies were summarised and presented in tabular forms and a descriptive report highlighting the key research findings (e.g. economic impacts, consequences of NMP, setting, NMP type, etc) of selected studies and the existing research gaps around NMP practice.

Patient and Public Involvement

No patients or public were involved in the study.

For peer review only - http://bmjopen.bmj.com/site/about/guidelines.xhtml

RESULTS

Database search findings

The database search generated 420 records. A total of 236 records were removed due to duplication. Of the remaining 184 records, we excluded 171 records in the initial review of titles and abstracts as these studies were not original research evaluating NMP. For the remaining 13 records, the full-text papers were independently reviewed by two reviewers, and a further four studies were excluded because they did not report resource use and economic impacts of NMP or did not fit within our definition of prescribing and medicine management activities. Nine studies were included in the final review (eight original research studies[7,15,50-55] and one grey literature paper[49]). Figure 1 shows the PRISMA flow chart of the included studies in our scoping review.

Figure 1, here

General characteristics of included studies

The key characteristics of included papers are summarised in Table 3 and Table C (Supplementary Data). Of the nine papers, six were from the UK,[7,15,49,51,54,55] two from Canada,[50,53] and one from Australia.[52] Papers were published between 2010 and 2022 and evaluated the impact of NMP practices by pharmacists (n=4), nurses (n=3),[7,50-55] physiotherapists and podiatrists (n=1),[15] and another estimating NMP cost-savings in primary and secondary care for a range of health professions.[49] Types of prescribing services evaluated in these studies included SP (n=2)[49,55] or IP (n=8)[7,15,49-54] and community nurse.[49]

For peer review only - http://bmjopen.bmj.com/site/about/guidelines.xhtml

Table 3: Overview and general characteristics of included studies

Authors (year)	Context (country/setting)	Study design	Type of prescribing	Comparator	Medicine management or prescribing activity	Study population
Black et al. (2022)[7]	UK, urban sexual health services	Mixed methods and a comparative case study (cost-consequence framework)	Nurse IP	PGD by non-prescribing nurses	Prescribed medications	N = 26 nurse prescribers<br>N = 67 PGDs users
Carey et al. (2020)[15]	UK mixed range of settings (primary and secondary care, social enterprise and private practice)	Mixed methods and a comparative case study (cost-consequence framework)	Physiotherapist IP<br>Podiatrist IP	Non-prescribing physiotherapists<br>Non-prescribing podiatrists	Prescribed and reviewed medications	N = 488 patients (243 IP sites and 245 NP sites)<br>N = 7 matched pairs of IP and NP sites (3 podiatrists and 4 physiotherapists)
Al Hamarneh et al. (2019)[50]	Canada, primary care (cardiovascular risk reduction)	Cost-effectiveness analysis (Markov model)	Pharmacist IP	Usual care	Prescribed and reviewed medications	The authors developed their model based on the population observed in R_XEACH trial,[56] as follows:<br>N = 723 patients (370 in intervention and 353 in control)<br>N = 54 pharmacies in the RCT
Hale et al. (2018)[52]	Australia, an elective surgery pre-admission clinic (venous thromboembolism)	Cost-effectiveness analysis (Decision tree model)	Pharmacist IP	Usual care	Prescribed medications	The authors developed their model based on the population observed in an earlier trial,[57] as follows:<br>N = 384 patients (194 in intervention and 190 in control)<br>N = 1 pharmacist prescriber<br>N = 59 medical prescribers
Marra et al. (2017)[53]	Canada, community care, hospitals, or primary care (hypertension)	Cost-effectiveness analysis (Markov model)	Pharmacist IP	Usual care	Prescribed medications	The authors developed their model based on the population observed in R_XACRION trial,[58] as follows:<br>N = 248 patients (181 in intervention and 67 in control)<br>N = 20 pharmacists practised in the community<br>N = 2 pharmacists from hospital outpatient clinics<br>N = 6 pharmacists from primary care clinics

For peer review only - http://bmjopen.bmj.com/site/about/guidelines.xhtml

Authors (year)	Context (country/setting)	Study design	Type of prescribing	Comparator	Medicine management or prescribing activity	Study population
i5 Health (2015)[49]	England, various settings (e.g. primary and secondary care)	Economic analysis of audits, self-reported questionnaire, interviews	IP and SP (for a range of professions, e.g. physiotherapists, podiatrists, midwives and radiographers)<br>Community nurse prescribers	NA	NA	Based on an estimation of the NMP practitioners registered with Northwest England NHS trusts (N = 1,566 unique prescribers)
Courtenay et al. (2015)[51]	England, primary care (type 2 diabetes)	Mixed methods and a comparative case study (cost-consequence framework)	Nurse IP	Non-prescribing nurses	Prescribed and reviewed medications, recommended decisions, provided advice, and discussed medications with GPs or colleagues	N = 12 general practices (6 prescribing nurses and 6 non-prescribing nurses)<br>N = 214 patients (131 in nurse prescriber sites and 83 in non-prescriber sites)
Neilson et al. (2015)[54]	UK, primary care (chronic pain)	Regression analysis of costs and effects; EVSI	Pharmacist IP	Usual care	Prescribed and reviewed medications	N = 6 general practices<br>N = 125 patients (39 in prescribing, 44 in review and 42 in usual care arms)<br>No information is provided about the number of non-medical prescribers in the two groups
Norman et al. (2010)[55]	UK, primary care (mental health)	Cost-consequences analysis; matched post-test control study	Nurse SP	Usual care	Prescribed medicines	N = 90 patients (45 matched pairs)<br>No information is provided about the numbers of prescribers in the two groups

Note. PGD: patient group direction; IP: independent prescribing; NP: non-prescribing; SP: supplementary prescribing; CVD: cardiovascular disease; EVSI: expected value of sample information analysis; HRQoL: health-related quality of life; NHS: National Health Service; OTC: over the counter; QALYs: Quality-adjusted life years. QALY is a generic metric used to value and quantify health outcomes in terms of both the quality and the quantity of life lived.[38]; RCT: randomised controlled trial; VTE: venous thromboembolism, which manifests as either deep vein thrombosis (DVT) or pulmonary embolism (PE); GP: general practitioner; NA: Not Available

For peer review only - http://bmjopen.bmj.com/site/about/guidelines.xhtml

Table C (available in Supplementary Data), here

Methodological and reporting considerations

Three out of nine papers conducted a model-based economic evaluation (i.e. cost-effectiveness analysis) using the outcomes from an earlier trial with an assessment of uncertainty (in the form of a deterministic and/or probabilistic sensitivity analysis).[50,52,53] Four studies conducted a cost-consequence-based approach listing costs and outcomes of NMP without assessing sources of uncertainty.[7,15,51,55] A bottom-up costing approach was used in most with clear information on costs per unit.[7,15,50-54] Overall non-model-based studies did not provide an explanation of sample size sufficiency. Only one study suggested that determining an optimal and larger sample size would be required to draw a precise and accurate conclusion.[54] Two studies failed to specify the number or characteristics of the study participants (e.g. non-medical prescribers or patients).[54,55]

Measures of costs

The resource use and costs evaluated in the included studies fall into the following three main categories:

i) Prescribing training course

Four out of nine articles applied direct costs associated with prescribing training and NMP courses (e.g. training course fee, supervision time, study time, etc),[7,50,53,54] with one study using time-off-work to complete the course.[55] Other relevant expenses such as out-of-pocket expenses (e.g. travel, accommodation, etc) by non-medical prescribers and their personal study time were included in two studies.[7,55]

ii) Prescription (and consultation)

For peer review only - http://bmjopen.bmj.com/site/about/guidelines.xhtml

Expenses applied in this category by some studies included rates of tests and other relevant services, referrals to other healthcare professionals, frequency of follow-up, time spent preparing for a prescribing consultation, time taken to prescribe, review or complete the medication plan for a patient, and the number of patients prescribed for, consultation frequency, time spent discussing the patient and obtaining prescriptions or clinical advice sought from GPs or other NMP practitioners, unplanned consultations for the health condition after the index consultation and frequency of new medications.[7,15,50-52,54,55] Incorrect or over-prescribing was identified and considered as an indication of wastage, and the 'wasted' medication, as well as under-prescribed medicine that should have been prescribed, were considered as another source of cost.[7,55]

iii) Other relevant expenses

Some studies also considered the expenses associated with service utilisation, e.g. hospital admissions, outpatient expenses, inpatient days and A&E visits,[15,51,53-55] or the healthcare and medical costs associated with targeted health conditions across case and control groups.[50,52,53]

Measures of outcome

Health-related quality of life was one of the main health outcomes evaluated using EQ-5D or SF-6D questionnaires,[15,50,52-54] and the benefits to patients of appropriate prescribing were measured in terms of increased quality-adjusted life years (QALYs) in some of these studies.[50,52-54] Multiple studies evaluated patient experience and satisfaction as one of the main patient outcomes.[7,15,51,55] Life years gained was applied by two studies.[50,53] Medicine adherence and ease of access to services were other outcomes reported in one study.[15] Examples of specific clinical and health outcomes used in the studies were self-care and relevant clinical indicators (e.g. HbA1c test results and body mass index) for patients with diabetes,[51] the reduced risk of the disease under study (e.g. venous thromboembolism, CVD)[50,52,53] and reduced blood pressure.[53]

For peer review only - http://bmjopen.bmj.com/site/about/guidelines.xhtml

Key findings: the costs and consequences of NMP

A summary of the key cost and consequence findings is provided for pharmacists, nurses and AHP professions in turn (see Table C for detailed information).

Pharmacists

The NMP practices by pharmacists were evaluated across a range of health conditions (e.g. venous thromboembolism, hypertension, etc) and significant improvements in health and clinical outcomes were reported at the end of the observation in three studies.[50,52,53] As such, Marra et al. [2017] found the 30-year risk of cardiovascular disease (CVD) in the pharmacist prescriber group was reduced from 0.61 in base-case to 0.41 (indicating a reduction of two CVD events in every 10 individuals receiving the intervention).[53] Although the intervention was associated with increased costs of CA$7,145 due to the intervention itself and medications, this was compensated for by a reduction of CA$15,094 in CVD and other co-morbidities costs, suggesting pharmacist independent prescribing was less costly and more effective than usual care.[53]

Consistent with Marra et al. [2017], two other studies (Al Hamarneh et al. [2019] and Hale et al. [2018]) reported that pharmacist prescribing was cost-effective and cost-saving for patients with CVD and venous thromboembolism, respectively.[50,52] Only Neilson et al. [2015] found that, relative to the usual-care arm, pharmacist prescribing for chronic pain was more costly (£77.5 for prescribing and £54.4 for review arms) and provided similar QALYs. Neilson et al. recruited a total sample of 125 patients in this RCT, but the authors recommended a larger sample size (between 460–690 for a threshold of £30,000 QALY gained or 540–780 for a threshold of £20,000 QALY gained) according to an expected value of sample information analysis.[54]

For peer review only - http://bmjopen.bmj.com/site/about/guidelines.xhtml

Nurses

Norman et al. [2010] indicated that patients in the mental health nurse prescriber group had a significantly higher level of satisfaction than those in the medical prescriber group.[55] Similarly, Courtenay et al. [2015] reported that the average patient satisfaction for some specified aspects of care was significantly higher among diabetic patients in the nurse prescriber group than among those of the non-prescribing nurses.[51] Nonetheless, no significant differences were reported with respect to patients' overall satisfaction by Courtenay et al. [2015] and Black et al. [2022]. Other specific or generic health and social outcomes were found to be similar among nurse prescribers and the control groups in these three studies.[7,51,55]

NMP was deemed resource-intensive to both providers and nurse prescribers. According to Black et al. [2022], the training-related costs included the course fee (paid fully by employers or training grants – ranging from £900 to £3,555 in 2016), an average of 7.4 paid study days (ranging from 2 to 13.7 day, incurring a cost of £6,451 to the NHS for each nurse – ranging from £1,283 to £11,138) and an average of 20.1 of study days for 92% of nurse prescribers (ranging from 1 to 31 funded by employer).[7] It is important to note however that although PGDs, provide a legal framework for health professionals to supply and administer a specified medicine to a pre-defined group of patients, there is no mandatory training required prior to their use. Employment costs of prescribing nurses were deemed potentially higher as they were on higher pay bands compared to non-prescribing nurses including PGD users.[7,51,55] Consultation durations and unplanned re-consultations were similar for both sexual health nurse prescribers and medical prescribers, as reported by Black et al. [2022].[7] However, Courtenay et al. [2015] reported longer consultations for patients with diabetes managed by nurse prescribers suggesting it was more costly relative to GPs and non-prescribing nurses.[51] No statistically significant differences in prescribing new medicines or use of other healthcare services between groups were identified in any of the three studies assessing this outcome.[7,51,55]

For peer review only - http://bmjopen.bmj.com/site/about/guidelines.xhtml

Allied health professionals

Only one study evaluated the benefits and costs of services by physiotherapist and podiatrist independent prescribers compared to non-prescribing physiotherapists and podiatrists.[15] Carey et al. [2020] showed the level of satisfaction with consultation and services was significantly higher in both non-medical prescriber groups. Patients of physiotherapist or podiatrist independent prescribers were more likely to receive medicine information or advice during consultations (39.7%) compared to patients managed by non-prescribers (24.5%). No significant differences were reported in quality of life in patients for all groups.[15] Consultation durations were longer for both prescriber groups, resulting in increased costs for prescribing physiotherapists (£7.95 per contact) and prescribing podiatrists (£8.62) compared to non-prescribers. No training-related costs were reported.[15]

DISCUSSION

Building on a previous systematic review[39] which included only three RCTs published before 2015, we have included a wider range of studies evaluating the effects, resource use and value for money in NMP. We used the PRISMA-ScR framework to guide the review, searched multiple databases and used snowballing techniques to improve the comprehensiveness of the study. Despite this, only one additional source of evidence from the grey literature was identified. The NMP literature has largely focused on assessing the benefits and effectiveness of prescribing authorities without evaluating the costs and resource use. Some other studies have concentrated on topics such as NMP trends and related national policies over time or implementation barriers and/or facilitators of NMP for different professions.[1,9,12,18,21,59] This review demonstrated the lack of evidence on costs, consequences and value for money in NMP by different groups of healthcare groups of healthcare professionals.

Our scoping review identified nine sources of evidence that evaluated economic impacts, resource use and consequences of NMP. Three studies showed pharmacist prescribing was

For peer review only - http://bmjopen.bmj.com/site/about/guidelines.xhtml

superior in all outcomes and cost-saving at large scale. Others reported similar results in most health, clinical and patient outcomes across other non-medical prescribers and control groups. NMP was deemed resource-intensive to both providers and other groups of non-medical prescribers (e.g. nurses, physiotherapists, podiatrists).

This scoping review revealed evidence sources were heterogeneous with regard to design, setting, range of cost and consequence outcomes, NMP types and comparators. In general, the existing evidence indicates that services provided by non-medical prescribers might positively influence patients' satisfaction with care, medication and their quality of life.[15,50,51,52,53] However, some of these findings came from non-RCT studies without robust evaluation of all relevant consequences and costs. Besides, some of these studies recruited small sample sizes, suggesting it is difficult to make any statement about the significance of the results beyond the sample included and therefore, these findings should be treated with caution.

The costs and consequences evidence on NMP has slowly grown since 2010, and appears to be concentrated in three countries, the UK, Canada and Australia, as prescribing rights are more developed in these countries. Despite the large increase in NMP in the UK and around the world and the increasing number of studies on NMP, there is still very limited information. Many papers evaluated NMP delivered by nurses and pharmacists using various sources of costs, health, clinical and patient outcomes with varied comparators for a range of health conditions, which limits their generalisability and usefulness for other settings and professions. Only three studies conducted a cost-effectiveness analysis to evaluate and demonstrate value for money in pharmacist prescribing.[50,51,53] Other studies used a cost-consequence approach (CCA) that provided disaggregated costs and outcomes of NMP for nurses, physiotherapists and podiatrists.[7,15,51,55] Although CCA helps identify and list relevant costs and outcomes associated with the interventions, it does not provide a definitive cost-outcome ratio and definite cost-effectiveness results for the interventions under study.[38]

For peer review only - http://bmjopen.bmj.com/site/about/guidelines.xhtml

The number of studies, particularly economic evaluation studies, assessing the economic burden, effects, and cost-effectiveness of NMP has been increasing, but there is still a dearth of evidence on the cost-effectiveness and value for money in NMP authorities by AHPs such as radiographers and dietitians. As most evidence relates to nurses and pharmacists, it is important to evaluate the impact, safety, resource use and economic value of prescribing by non-medical prescribers in other professions to inform policy and practice around NMP where it provides value for money. It is also important to acknowledge and further explore the challenges related to capturing these data, as NMP has been introduced as an additional role for healthcare professions, and hence it is not easy to separate and capture some of the added costs and values in terms of these additional prescriptive authorities.[15,22]

There seem to be research-quality gaps in the literature. Although we did not assess the quality of included studies, some of the studies have performed non-model-based analysis using small samples that might affect the analysis, and in some cases, the main outcomes and sources of costs (e.g. training-related, etc) were not included in the analysis. Despite the importance of rigour in quantitative research, sample size reporting and sufficiency assessment remained inconsistent and partial in these studies.

Strengths and limitations

A rigorous search was conducted, allowing for a diverse set of literature (from both peer-reviewed and grey) to be identified in a robust and reproducible manner. This review also included the findings from RCT studies that were published before 2015 and was not considered by the previous review by Noblet et al. (2018).[39] To our knowledge, this is the first scoping review covering and representing the largest and most up-to-date evidence on costs and consequences of prescribing practices by nurses, pharmacists and AHPs. This scoping review contributes to the discussion of the costs and consequences of NMP and the existing research gaps regarding value for money in NMP for different groups of healthcare professionals. Original studies that did not report resource use and costs associated with NMP

For peer review only - http://bmjopen.bmj.com/site/about/guidelines.xhtml

were not included in our review. While this strategy contributed to a more focused search, studies that reported only the effectiveness and benefits of NMP practices without evaluating costs are missing. Comparison of studies was challenged by heterogeneity regarding the profession, type of NMP, costs and consequences evaluated.

CONCLUSION

NMP practice is now an integrated feature of healthcare delivery in the UK and around the world, but considerable uncertainty remains regarding the costs, consequences and cost-effectiveness of the prescribing rights granted to AHPs, including therapeutic radiographers and dietitians. In order to determine accurate mean values and detect cost and benefit differences across non-medical prescribers and control groups it is important that future studies involve larger and more representative samples with greater power. Adopting a model-based approach within each profession using targeted outcome measures would also enable a more robust comparison and improve understanding how to best utilise NMP to ensure it offers a cost-effective solution to providing faster access to medicine and healthcare services for patients.

For peer review only - http://bmjopen.bmj.com/site/about/guidelines.xhtml

Acknowledgements: None.

Author Contributions: SB: Study design; formulation of search strategies; investigation, screening and review – titles, abstracts and full-texts; data extraction; writing – original draft, review & editing. NH, NC, YJ, KH: Study design; formulation of search strategies; screening and review – full-texts, data extraction; writing – review & editing. All authors read and approved the final version of the manuscript before submission.

Funding: This scoping review is conducted as part of the health economic evaluation in the TRaDiP project that was funded by the Department of Health Policy Research Programme, the National Institute for Health Research (grant number: PRP12697/86202356).

Conflict of interest: Authors declare no competing interest.

Patient and public involvement: Patients and/or the public were not involved in the design, or conduct, or reporting or dissemination plans of this scoping review.

Data availability: Data are available upon reasonable request.

Ethics statements

Ethics approval: This scoping review study does not require ethical approval as it consists of reviewing and collecting secondary data from already published papers.

Patient consent for publication: Not required.

For peer review only

REFERENCES

1. Cooper R, Bissell P, Ward P, et al. Further challenges to medical dominance? The case of nurse and pharmacist supplementary prescribing. *Health & Social Care in the Community* 2012;**16**(2):115–133. https://doi.org/10.1177/1363459310364159.
2. Cooper R, Guillaume L, Avery T, et al. Nonmedical prescribing in the United Kingdom: developments and stakeholder interests. *J Ambul Care Manage* 2008;**31**(3):244-52. https://doi.org/10.1097/01.JAC.0000324670.91153.b4.
3. World Health Organization (WHO). Ageing and health. WHO, 2021. https://www.who.int/news-room/fact-sheets/detail/ageing-and-health. [Accessed 12 March 2022].
4. Maresova P, Javanmardi E, Barakovic S, et al. Consequences of chronic diseases and other limitations associated with old age - a scoping review. *BMC Public Health* 2019;**19**(1):1431. https://doi.org/10.1186/s12889-019-7762-5.
5. Kingston A, Comas-Herrera A, Jagger C, et al. Forecasting the care needs of the older population in England over the next 20 years: estimates from the Population Ageing and Care Simulation (PACSim) modelling study. *Lancet Public Health* 2018;**3**(9):e447-e455. https://doi.org/10.1016/S2468-2667(18)30118-X.
6. Nancarrow SA, Borthwick AM. Dynamic professional boundaries in the healthcare workforce. *Sociol Health Illn* 2005;**27**(7):897-919. https://doi.org/10.1111/j.1467-9566.2005.00463.x.
7. Black A, Courtenay M, Norton C, et al. Independent nurse medication provision: A mixed method study assessing impact on patients' experience, processes, and costs in sexual health clinics. *J Adv Nurs* 2022;**78**(1):239-251. https://doi.org/10.1111/jan.15075.
8. Kroezen M, van Dijk L, Groenewegen PP, et al. Nurse prescribing of medicines in Western European and Anglo-Saxon countries: a systematic review of the literature. *BMC Health Serv Res* 2011;**11**:127. https://doi.org/10.1186/1472-6963-11-127.
9. Graham-Clarke E, Rushton A, Noblet T, et al. Non-medical prescribing in the United Kingdom National Health Service: A systematic policy review. *PLoS One* 2019;**14**(7):e0214630. https://doi.org/10.1371/journal.pone.0214630.
10. Cope LC, Abuzour AS, Tully MP. Nonmedical prescribing: where are we now? *Ther Adv Drug Saf* 2016;**7**(4):165-72. https://doi.org/10.1177/2042098616646726.
11. Department of Health and Social Security (DHSS). Medicinal Products; Prescriptions by Nurses etc Act, 1992. https://www.legislation.gov.uk/ukpga/1992/28/contents [Accessed 10 February 2022].
12. Courtenay M, Carey N, Stenner K. Non medical prescribing leads views on their role and the implementation of non medical prescribing from a multi-organisational perspective. *BMC Health Serv Res* 2011;**11**:142. https://doi.org/10.1186/1472-6963-11-142.
13. Allied Health Professions Federation. Outline curriculum framework for education programmes to prepare physiotherapists and podiatrists as independent/supplementary prescribers and to prepare radiographers as supplementary prescribers, 2013. http://www.ahpf.org.uk/AHP_Prescribing_Programme_Information.htm [Accessed 10 March 2022].
14. Rumney N. Optometry and independent prescribing. *Journal of Prescribing Practice* 2019;**1**(2):87-92. https://doi.org/10.12968/jprp.2019.1.2.87.
15. Carey N, Edwards J, Otter S, et al. A comparative case study of prescribing and non-prescribing physiotherapists and podiatrists. *BMC Health Serv Res* 2020;**20**(1):1074. https://doi.org/10.1186/s12913-020-05918-8.
16. Morris L. Nonmedical independent prescribing: Podiatrists to take the next step. *The Diabetic Foot Journal* 2014;**16**:146–50.
17. National Health Service. Non-medical prescribing by allied health professionals, 2016. https://www.england.nhs.uk/ahp/med-project/ [Accessed 10 March 2022].
18. Stenner K, van Even S, Collen A. Paramedic independent prescribing: a qualitative study of early adopters in the UK. *Br Paramed J* 2021;**6**(1):30-37. https://doi.org/10.29045/14784726.2021.6.6.1.30.

For peer review only - http://bmjopen.bmj.com/site/about/guidelines.xhtml

19. Zhou M, Desborough J, Parkinson A, et al. Barriers to pharmacist prescribing: a scoping review comparing the UK, New Zealand, Canadian and Australian experiences. *Int J Pharm Pract* 2019;**27**(6):479-489. https://doi.org/10.1111/ijpp.12557.

20. Emmerton L, Marriott J, Bessell T, et al. Pharmacists and prescribing rights: review of international developments. *J Pharm Pharm Sci* 2005;**8**(2):217-225.

21. Weeks G, George J, Maclure K, et al. Non-medical prescribing versus medical prescribing for acute and chronic disease management in primary and secondary care. *Cochrane Database Syst Rev* 2016;**11**(11):CD011227. https://doi.org/10.1002/14651858.CD011227.pub2.

22. Stewart D, Jebara T, Cunningham S, et al. Future perspectives on nonmedical prescribing. *Ther Adv Drug Saf* 2017;**8**(6):183-197. https://doi.org/10.1177/2042098617693546.

23. Salgado T, Rosenthal M, Coe A, et al. Primary healthcare policy and vision for community pharmacy and pharmacists in the United States. *Pharm Pract (Granada)* 2020;**18**(3):1-16. https://doi.org/10.18549/pharmpract.2020.3.2160.

24. Gadbois E, Miller E, Tyler D, et al. Trends in State Regulation of Nurse Practitioners and Physician Assistants, 2001 to 2010. *Med Care Res and Rev* 2015;**72**(2):200-219. https://doi.org/10.1177/1077558714563763.

25. Maier CB. Nurse prescribing of medicines in 13 European countries. *Hum Resour Health* 2019;**17**(1):95. https://doi.org/10.1186/s12960-019-0429-6.

26. Wilson D, Murphy J, Nam M, et al. Nurse and midwifery prescribing in Ireland: A scope-of-practice development for worldwide consideration. *Nurs Health Sci* 2018;**20**:264–70. https://doi.org/10.1111/nhs.12408.

27. Department of Health. The National Health System plan: A plan for investment. A plan for reform, 2000. http://1nj5ms2lli5hdggbe3mm7ms5.wpengine.netdna-cdn.com/files/2010/03/pnsuk1.pdf. [Accessed 12 August 2021].

28. Health and Care Professions Council. Non-medical prescribing approved courses. Health and Care Professions Council; 2021. https://www.hcpc-uk.org/education/approved-programmes/. [Accessed 10 August 2021].

29. General Pharmaceutical Council. Discussion paper on supervising pharmacist independent prescribers in training, 2016. https://www.pharmacyregulation.org/sites/default/files/discussion_paper_on_supervising_pharmacist_independent_prescribers_in_training_november_2016.pdf [Accessed 12 March 2021].

30. Nursing and Midwifery Council. Standards for prescribing programmes, 2018. https://www.nmc.org.uk/standards/standards-for-post-registration/standards-for-prescribers/standards-for-prescribing-programmes/ [Accessed 14 March 2021].

31. General Optical Council. Independent Prescribing, 2021. https://optical.org/en/news/news-and-press-releases/goc-council-approves-updated-requirements-for-independent-prescribing-categories/ [Accessed 10 March 2021].

32. Black A, Gage H, Norton C, et al. Patient satisfaction with medication consultations and medicines information provided by nurses working autonomously in sexual health services: A questionnaire study. *J Adv Nurs* 2022;**78**(2):523-531. https://doi.org/10.1111/jan.15087.

33. Sharman A. Independent prescribing: a journey to provide the best possible care. *Journal of Paramedic Practice* 2015;**7**(5):228-232. https://doi.org/10.12968/jpar.2015.7.5.228.

34. Department of Health. Improving patients' access to medicines: A guide to implementing nurse and pharmacist independent prescribing within the NHS in England, 2006. Available at: http://webarchive.nationalarchives.gov.uk/. [Accessed 15 July 2021].

35. National Health Service England. Allied health professions medicines project, 2016. https://www.england.nhs.uk/ahp/med-project/ [Accessed 15 March 2021].

36. Babashahi S, Carey N, Hart K, et al. Costs and consequences of services provided by non-medical prescribers: a scoping review protocol. *Journal of Prescribing Practice* 2022;**4**(4):160-164. https://doi.org/10.12968/jprp.2022.4.4.160.

37. Department of Health. (2000). Health Services Circular (HSC 2000/026): Patient Group Directions. http://webar chive.natio nalarchives.gov.uk/20130 10710 5354/http:/www.dh.gov.uk/prod_consum_dh/group s/dh_digit alass ets/@dh/@en/docum ents/digit alass et/dh_40122 60.pdf. [Accessed 15 July 2021].

For peer review only - http://bmjopen.bmj.com/site/about/guidelines.xhtml

38. Drummond M, Sculpher M, Torrance G. Methods for the economic evaluation of health care programmes. Oxford University Press: Oxford, 2005.

39. Noblet T, Marriott J, Graham-Clarke E, et al. Clinical and cost-effectiveness of non-medical prescribing: A systematic review of randomised controlled trials. *PLoS One* 2018;**13**(3):e0193286. https://doi.org/10.1371/journal.pone.0193286.

40. Alghamdi SSA, Hodson K, Deslandes P, et al. Prescribing trends over time by non-medical independent prescribers in primary care settings across Wales (2011-2018): a secondary database analysis. *BMJ Open* 2020;**10**(10):e036379. https://doi.org/10.1136/bmjopen-2019-036379.

41. Courtenay M, Carey N, Stenner K. An overiew of non medical prescribing across one strategic health authority: a questionnaire survey. *BMC Health Serv Res* 2012;**12**:138. https://doi.org/10.1186/1472-6963-12-138.

42. Graham-Clarke E, Rushton A, Marriott J. A Delphi study to explore and gain consensus regarding the most important barriers and facilitators affecting physiotherapist and pharmacist non-medical prescribing. *PLoS One* 2021;**16**(2):e0246273. https://doi.org/10.1371/journal.pone.0246273.

43. Arksey H, O'Malley L. Scoping studies: towards a methodological framework. *International Journal of Social Research Methodology* 2005;**8**:19-32. https://doi.org/10.1080/1364557032000119616.

44. Levac D, Colquhoun H, O'Brien K. Scoping studies: advancing the methodology. *Implementation Sci* 2010;**5**(1):1-9. https://doi.org/10.1186/1748-5908-5-69.

45. JBI. *The Joanna Briggs Institute Reviewers' Manual 2015: Methodology for JBI Scoping Reviews*, 2015. The Joanna Briggs Institute. www.joannabriggs.org. [Accessed 15 July 2021].

46. Tricco AC, Lillie E, Zarin W, et al. PRISMA Extension for Scoping Reviews (PRISMA-ScR): Checklist and Explanation. *Ann Intern Med* 2018;**169**(7):467-473. https://doi.org/10.7326/M18-0850.

47. Gielen SC, Dekker J, Francke AL, et al. The effects of nurse prescribing: a systematic review. *Int J Nurs Stud* 2014;**51**(7):1048-61. https://doi.org/10.1016/j.ijnurstu.2013.12.003.

48. Petticrew M Roberts H. (2006). Systematic reviews in the social sciences: a practical guide. Blackwell Publishing. https://doi.org/10.1002/9780470754887

49. i5 Health. Non-medical prescribing (NMP): An economic evaluation. National Health System: Health Education North West 2015. http://www.i5health.com/NMP/NMPEconomicEvaluation.pdf

50. Al Hamarneh YN, Johnston K, Marra CA, et al. Pharmacist prescribing and care improves cardiovascular risk, but is it cost-effective? A cost-effectiveness analysis of the RxEACH study. *Can Pharm J (Ott)* 2019;**152**(4):257-266. https://doi.org/10.1177/1715163519851822.

51. Courtenay M, Carey N, Gage H, et al. A comparison of prescribing and non-prescribing nurses in the management of people with diabetes. *J Adv Nurs* 2015;**71**(12):2950-64. https://doi.org/10.1111/jan.12757.

52. Hale A, Merlo G, Nissen L, et al. Cost-effectiveness analysis of doctor-pharmacist collaborative prescribing for venous thromboembolism in high risk surgical patients. *BMC Health Serv Res* 2018;**18**(1):749. https://doi.org/10.1186/s12913-018-3557-0.

53. Marra C, Johnston K, Santschi V, et al. Cost-effectiveness of pharmacist care for managing hypertension in Canada. *Can Pharm J (Ott)* 2017;**150**(3):184-197. https://doi.org/10.1177/1715163517701109.

54. Neilson AR, Bruhn H, Bond CM, et al. Pharmacist-led management of chronic pain in primary care: costs and benefits in a pilot randomised controlled trial. *BMJ Open* 2015;**5**(4):e006874. https://doi.org/10.1136/bmjopen-2014-006874.

55. Norman IJ, Coster S, McCrone P, et al. A comparison of the clinical effectiveness and costs of mental health nurse supplementary prescribing and independent medical prescribing: a post-test control group study. *BMC Health Serv Res* 2010;**10**:4. https://doi.org/10.1186/1472-6963-10-4.

56. Tsuyuki RT, Al Hamarneh YN, Jones CA, et al. The Effectiveness of Pharmacist Interventions on Cardiovascular Risk: The Multicenter Randomized Controlled RxEACH Trial. *J Am Coll Cardiol* 2016;**67**(24):2846-54. https://doi.org/10.1016/j.jacc.2016.03.528.

For peer review only - http://bmjopen.bmj.com/site/about/guidelines.xhtml

57. Hale AR, Coombes ID, Stokes J, et al. Perioperative medication management: expanding the role of the preadmission clinic pharmacist in a single centre, randomised controlled trial of collaborative prescribing. *BMJ Open* 2013;**3**(7):e003027. https://doi.org/10.1136/bmjopen-2013-003027.

58. Tsuyuki RT, Houle SK, Charrois TL, et al. Randomized Trial of the Effect of Pharmacist Prescribing on Improving Blood Pressure in the Community: The Alberta Clinical Trial in Optimizing Hypertension (RxACTION). *Circulation* 2015;**132**(2):93-100. https://doi.org/10.1161/CIRCULATIONAHA.115.015464.

59. Edwards J, Coward M, Carey N. Barriers and facilitators to implementation of non-medical independent prescribing in primary care in the UK: a qualitative systematic review. BMJ Open 2022;12:e052227. https://doi.org/10.1136/ bmjopen-2021-052227.

For peer review only

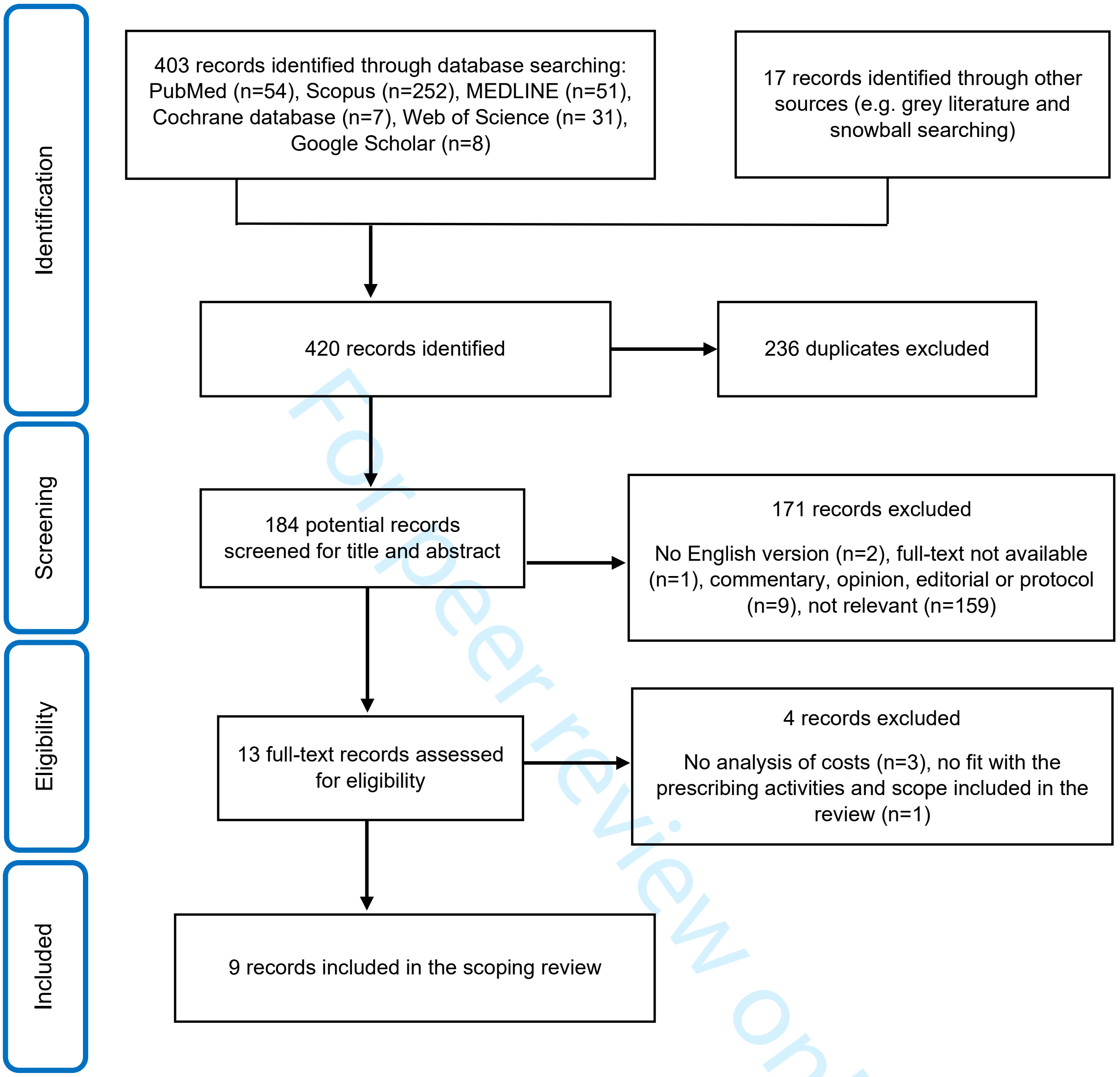

Figure 1. PRISMA flowchart of the study selection process

Costs, consequences and value for money in non-medical prescribing: A scoping review

Supplementary Data

Table A.1 Search terms used in PubMed from 1 January 1999 to 1 January 2022

**Database**: PubMed		
**Date of search**: 14 January 2022		
**Search step**	**Search terms and queries**	**Results**
S1	"non-medical prescribing" OR "non-medical prescriber" OR "non-medical prescribers" OR "non-doctor prescribing" OR "non-doctor prescriber" OR "non-doctor prescribers" [All Fields]	216
S2	"non-medical prescribing" OR "non-medical prescriber" OR "non-medical prescribers" OR "non-doctor prescribing" OR "non-doctor prescriber" OR "non-doctor prescribers" [All Fields] Filters: from 1999/1/1 - 2022/1/1	215
S3	"medicine management" AND activities [All Fields] Filters: from 1999/1/1 - 2022/1/1	47
S4	S2 OR S3	262
S5	(outcome OR consequence OR effectiveness OR impact) AND (economic OR cost) [All Fields] Filters: from 1999/1/1 - 2022/1/1	555,862
S6	S4 AND S5	28
S7	"allied health profession" OR "allied health professions" OR "allied health professional" OR "allied health professionals" OR "non-medical profession" OR "non-medical professions" OR “non-medical professional" OR "non-medical professionals" OR nurse OR pharmacist OR dietitian OR dietician OR radiographer OR podiatrist OR physiotherapist OR podiatrist OR optometrist OR paramedic OR midwife [All Fields] Filters: from 1999/1/1 - 2022/1/1	563,041
S8	S6 AND S7	26

Table A.2 Search terms used in MEDLINE from 1 January 1999 to 1 January 2022

**Database**: MEDLINE		
**Date of search**: 14 January 2022		
**Step**	**Search terms and queries**	**Results**
S1	"non-medical prescribing" OR "non-medical prescriber" OR "non-medical prescribers" OR "non-doctor prescribing" OR "non-doctor prescriber" OR "non-doctor prescribers" OR "medicine management activities" in Anywhere	212
S2	"non-medical prescribing" OR "non-medical prescriber" OR "non-medical prescribers" OR "medicine management activities" in Anywhere Narrowed by: Entered date: 1999-01-01 to 2022-01-01	211

For peer review only - http://bmjopen.bmj.com/site/about/guidelines.xhtml

S3	(outcome OR consequence OR effectiveness OR impact) AND (economic OR cost) in Anywhere	356,562
S4	S2 AND S3	26
S5	"allied health profession" OR "allied health professions" OR "allied health professional" OR "allied health professionals" OR "non-medical profession" OR "non-medical professions" OR “non-medical professional" OR "non-medical professionals" OR nurse OR pharmacist OR dietitian OR radiographer OR podiatrist OR physiotherapist OR podiatrist OR optometrist OR paramedic OR midwife in Anywhere	591,003
S6	S5 AND S4	25

Table A.3 Search terms used in Scopus from 1 January 1999 to 1 January 2022

**Database**: Scopus		
**Date of search**: 14 January 2022		
**Step**	**Search terms and queries**	**Results**
S1	ALL ("non-medical prescribing" OR "non-medical prescriber" OR "non-medical prescribers" OR "non-doctor prescribing" OR "non-doctor prescriber" OR "non-doctor prescribers") AND PUBYEAR > 1998	830
S2	ALL ("medicine management activities") AND PUBYEAR > 1998	11
S3	S1 OR S2	835
S4	ALL ((outcome OR consequence OR effectiveness OR impact) AND (economic OR cost)) AND PUBYEAR > 1998	4,456,222
S5	S3 AND S4	266
S6	ALL ("allied health profession" OR "allied health professions" OR "allied health professional" OR "allied health professionals" OR "non-medical profession" OR "non-medical professions" OR "non-medical professional" OR "non-medical professionals" OR nurse OR pharmacist OR dietitian OR radiographer OR podiatrist OR physiotherapist OR podiatrist OR optometrist OR paramedic OR midwife) AND PUBYEAR > 1998	1,091,538
S7	S5 AND S6	252

Table A.4 Search terms used in Web of Science from 1 January 1999 to 1 January 2022

**Database**: Web of Science		
**Date of search**: 14 January 2022		
**Step**	**Search terms and queries**	**Results**
S1	ALL=("non-medical prescribing" OR "non-medical prescriber" OR "non-medical prescribers" OR "non-doctor prescribing" OR "non-doctor prescriber" OR "non-doctor prescribers" OR "medicine management activities") Timespan: 1999-01-01 to 2022-01-01 (Publication date)	186
S2	ALL=(cost or economic) AND ALL=(outcome OR consequence OR impact OR effectiveness)	825,022
S3	S1 AND S2	17
S4	ALL=("allied health profession" OR "allied health professions" OR "allied health professional" OR "allied health professionals" OR "non-medical	407,152

For peer review only

	profession" OR "non-medical professions" OR “non-medical professional" OR "non-medical professionals" OR dietitian OR dietician OR radiographer OR therapist OR podiatrist OR physiotherapist OR paramedic OR optometrist OR midwife OR nurse OR pharmacist) Timespan: 1999-01-01 to 2022-01-01 (Publication date)	
S5	S3 AND S4	14

Table A.5 Search terms used in Cochrane Database of Systematic Reviews from 1 January 1999 to 1 January 2022

**Database**: Cochrane Database of Systematic Reviews		
**Date of search**: 14 January 2022		
**Step**	**Search terms and queries**	**Results**
S1	"non-medical prescribing" OR "non-medical prescriber" OR "non-medical prescribers" OR "non-doctor prescribing" OR "non-doctor prescriber" OR "medicine management activities" in All Texts	7
S2	(outcome OR effectiveness OR consequence OR impact) AND (cost or economic) in All Texts	5,675
S3	S1 AND S2	7

For peer review only

Table C: Cost and outcome measures and key findings of included studies

Authors (year)	Type of prescribing	Outcome measures	Cost and resource use measures	Perspective	Study main findings
Black et al. (2022)[7]	Nurse IP	Patient consultation experience; patient satisfaction with information about medicines; medication appropriateness; medication effectiveness for the condition using the number of unplanned re-consultations in 3 months	From the NHS perspective: NMP course training and governance (staff supervision, study time and backfill); clinic processes (including medication provision, error, appropriateness, consultation duration, impact on the workload of other professionals, rates of unplanned re-consultations).<br>From nurses' perspective: study leave; personal time to study; out-of-pocket expenses for travel and purchase of learning resources for training; prospect promotions.	UK NHS, nurses and patients	***Consequences***<br>- Patient satisfaction: over 96% in both groups.<br>- Medication decisions safety: 96% for nurse prescribers vs 99% for PGD users.<br>- Medication errors: minor for both nurse prescribers (56%) and PGD users (62%), mainly documentation-related (78%), with no patients harmed.<br>- Consultation duration and unplanned re-consultations: similar for both groups. Nurse prescribers sought assistance from colleagues less frequently but provided longer consultations.<br>***Costs***<br>- NMP training fees: fully paid by their employers or health grants (£900 to £3,555) in 2016.<br>- Paid study days: An average of 7.4 study days, including clinical supervision, for each nurse (an average cost of £6,45 to the NHS per nurse) and an average of 20.1 employer-funded study days were reported by 92% of nurses.<br>- PGD-related costs to the employer: £912 for creating a new PGD and £276 for updating.<br>- Medication costs: the average costs per patient were higher for the nurse prescriber group (£19) than for PGD users (£11.25).<br>- Nurse prescribers were in higher pay bands.
Carey et al. (2020)[15]	Physiotherapist IP<br>Podiatrist IP	Patient satisfaction; ease of access to services; quality of life	Rates of relevant tests or services ordered; frequency of new medicines; referrals to other health professionals; frequency of follow-up; consultations numbers and durations; time spent discussing the patient with other colleagues; unplanned consultations for the same condition within two months of the index consultation.	NA	***Consequences***<br>- Acceptability of independent prescribing: high (77%), with 23% preferring a GP to prescribe.<br>- Quality of life and patient satisfaction with services: significant improvements between baseline and follow-up with no significant differences in both groups.<br>***Costs***<br>- Consultation duration: An average of 6.8 min higher for physiotherapist prescribers and 3.5 min for podiatrist prescribers compared to non-prescribers.<br>- The prescriber podiatrists more frequently ordered medications and tests than non-prescribing colleagues. No cost was calculated and reported.<br>- Follow-up consultations: no significant differences in both groups.<br>- Cost of consultations: An average of £7.95 for physiotherapist prescribers and £8.62 for podiatrist prescribers higher than non-prescriber groups.<br>- No NMP training-related costs (training course, travel, supervision time, etc) were reported.
Al Hamarneh et al. (2019)[50]	Pharmacist IP	QALYs; life years and CVD risk	Cost of baseline and follow-up visits, cost of half-day training and direct medical costs (e.g. inpatient, outpatient, medication, etc)	Canadian Medicare	***Consequences***<br>- Life years: increased by 0.11 per patient; QALYs: increased by 0.19; risk of CVD: decreased by 0.10.<br>***Costs***<br>- Medical costs: decreased by CA$2,149 compared to usual care.<br>- Cost of pharmacist intervention – 1st year: CA$233 [baseline visit: CA$125 (baseline); CA$25 (per follow-up) and cost of half-day training: CA$500 (CA$33.3 per patient)].<br>- Cost of pharmacist intervention – 2nd year: CA$175.<br>***Cost-effectiveness***

For peer review only - http://bmjopen.bmj.com/site/about/guidelines.xhtml

Authors (year)	Type of prescribing	Outcome measures	Cost and resource use measures	Perspective	Study main findings
					- Pharmacist prescribing was estimated to save more than CA$4.4 billion, add 576,689 QALYs and prevent more than 8.9 million CV events over 30 years if applied to only 15% of the eligible patients in Canada.
Hale et al. (2018)[52]	Pharmacist IP	QALYs	Annual costs of existing and new pharmacy services; time taken by the pharmacist to prescribe (minus time saved for medical prescriber); annual salary of pharmacist (minus time costs offset by medical prescribers not having to prescribe); direct costs of acute DVT and PE to the healthcare system	Australian healthcare sector	***Consequences***<br>- QALYs: increased by 0.02 per patient in the pharmacist prescriber group.<br>- The proportion of treated VTE patients: 100% in the pharmacist prescriber and 91% in the GP groups.<br>***Costs***<br>- Cost of introducing the pharmacist IP: AU$2.24 per patient (calculated based on annual pharmacist salary and overhead costs minus GP time not required to prescribe).<br>***Cost-effectiveness***<br>- The probability of pharmacist prescribing and care being cost-effective at a willingness-to-pay of $AU40,000 was 95%.
Marra et al. (2017)[53]	Pharmacist IP	Reduced systolic blood pressure in patients with hypertension; relative risk of CVD; relative risk of renal disease; hazard ratio of mortality after CVD; life years; and HRQoL	Direct medical costs of the health conditions and the costs associated with implementing the pharmacist intervention strategy and training.	Canadian Medicare	***Consequences***<br>- Blood pressure and CVD cases: reduced by 0.21 per patient in the pharmacist prescribing group<br>- Life years: increased by 0.3 in the pharmacist prescribing group<br>- QALYs: increased by 0.4 in the pharmacist prescribing group<br>***Costs***<br>- Cost of pharmacist intervention – 1st year: CA$200 [baseline visit: CA$125 (baseline); CA$25 (per follow-up) and cost of half-day training: CA$500 (CA$33.3 per patient)].<br>- Cost of pharmacist intervention – 2nd year: CA$75.<br>- Cost of pharmacist intervention – 3rd year: CA$50.<br>***Cost-effectiveness***<br>- The pharmacist prescribing was found cost-effective and cost-saving (the reduction in costs associated with CVD and ESRD equated to cost-savings of CA$6,365 per patient in the pharmacist group over 30 years.
i5 Health (2015)[49]	IP and SP (for a range of healthcare professions, e.g. physiotherapists, pharmacists and radiographers)<br>Community nurse prescribers	NA	Number of consultations; visits and referrals to medical prescribers avoided; lower A&E attendance and waiting times; avoided readmissions; inappropriate prescribing	NA	***Consequences***<br>- NMP practitioners provided information on patient's medications in 99% of reviews and identified inappropriate medication regimens in 50% of appointments.<br>***Costs***<br>- As shown by the i5 Health analysis of the audits for different non-medical prescriber groups in primary and secondary care in England, the main savings of NMP practices included 32% of GP appointments and 31% of follow-up appointments by a consultant.<br>- The annual value contributed by adding one new non-medical prescriber was over £270 million. The total cost-savings were predicted to be approximately £800 million in England in 2014.<br>- No NMP training-related costs (training course, travel, supervision time, etc) were reported.
Courtenay et al. (2015)[51]	Nurse IP	Patient-reported diabetes self-care activities; HbA1c test results; patients' satisfaction with activities, process and medicine management	Employment of cost; consultation cost; advice-seeking from other professionals and GP prescribing signing; prescribing costs; use of other health services	UK NHS	***Consequences***<br>- Patients satisfaction: in the nurse prescriber groups, patients were more satisfied with some specific aspects of care. However, there were no significant differences in general and overall satisfaction.<br>- Diabetes self-care activities: no significant differences were found.

For peer review only - http://bmjopen.bmj.com/site/about/guidelines.xhtml

Authors (year)	Type of prescribing	Outcome measures	Cost and resource use measures	Perspective	Study main findings
					- HbA1c test result: The HbA1c score decreased significantly in both groups over six months.<br>- Frequency of consultations: no significant differences were found.<br>***Costs***<br>- Consultation duration: longer consultations (average, 7.7 min) were provided by nurse prescribers for an additional cost of £6.<br>- New prescriptions and the use of healthcare services: no significant differences were reported regarding by patients in both groups.<br>- Most prescribing nurses were on a higher salary band than non-prescribing colleagues.
Neilson et al. (2015)[54]	Pharmacist IP	QALYs; chronic pain grade; anxiety and depression scale	Pharmacist training, pharmacist and GP time to deliver the intervention and provide follow-up; the number of hospitalisation days and outpatient visits); primary care visits for chronic pain (GP, nurse, healthcare assistant); telephone contacts for chronic pain; prescribed and non-prescribed OTC pain-related medications and health service resource use	UK NHS	***Consequences***<br>- QALYs: very small in both prescribing and review groups (relative to usual care, the mean differences in QALYs were 0.0069 for prescribing and 0.0097 for review groups, respectively)<br>***Costs***<br>- The average cost differences per patient were £77 for prescribing and £54 for review groups relative to usual care.<br>Overall, based on the EVSI, the authors suggested a larger sample size (e.g. the optimal sample size was estimated at 780 for prescribing and 540 for the review group using a cost per QALY threshold of £20,000) for reliable findings.
Norman et al. (2010)[55]	Nurse SP	Medication adherence; health status; adverse effects; patient satisfaction (with information, consultation, treatment, etc), patient's perception of improvement in their health problem; social functioning and impairment; depression scale	Training costs (time off work and other expenses, e.g. travel, supervision time, etc); costs of prescribing (e.g. time spent preparing for a prescribing, time taken to prescribe for a patient and number of patients prescribed for); patients' use of healthcare; cost of service per patient	Health and social care	***Consequences***<br>- Medicine adherence, health status, side effects, and satisfaction with overall care: no significant differences were reported between patients across the nurse and medical prescriber groups.<br>***Costs***<br>- Psychiatric inpatient costs were an average of £1,186 significantly higher per patient for the nurse prescriber group than those in the medical prescriber group.<br>- Total annual costs per patient: no significant differences were reported.<br>- The prescribing training course fee: £497 per patient.<br>- No additional training-related costs (travel, supervision time, etc) were reported.

Note. NMP: non-medical prescribing; PGD: patient group direction; IP: independent prescribing; SP: supplementary prescribing; CVD: cardiovascular disease; HRQoL: health-related quality of life; NHS: National Health Service; OTC: over the counter; QALYs: quality-adjusted life years; VTE: venous thromboembolism, which manifests as either deep vein thrombosis (DVT) or pulmonary embolism (PE); GP: general practitioners; NA: not available; EVSI: expected value of sample information analysis.

For peer review only - http://bmjopen.bmj.com/site/about/guidelines.xhtml

Costs, consequences and value for money in non-medical prescribing: A scoping review

Table B. Preferred Reporting Items for Systematic reviews and Meta-Analyses extension for Scoping Reviews (PRISMA-ScR) Checklist

SECTION	ITEM	PRISMA-ScR CHECKLIST ITEM	REPORTED ON PAGE #
**TITLE**			
Title	1	Identify the report as a scoping review.	1
**ABSTRACT**			
Structured summary	2	Provide a structured summary that includes (as applicable): background, objectives, eligibility criteria, sources of evidence, charting methods, results, and conclusions that relate to the review questions and objectives.	2
**INTRODUCTION**			
Rationale	3	Describe the rationale for the review in the context of what is already known. Explain why the review questions/objectives lend themselves to a scoping review approach.	5
Objectives	4	Provide an explicit statement of the questions and objectives being addressed with reference to their key elements (e.g., population or participants, concepts, and context) or other relevant key elements used to conceptualise the review questions and/or objectives.	4, 5-8
**METHODS**			
Protocol and registration	5	Indicate whether a review protocol exists; state if and where it can be accessed (e.g., a Web address); and if available, provide registration information, including the registration number.	6
Eligibility criteria	6	Specify characteristics of the sources of evidence used as eligibility criteria (e.g., years considered, language, and publication status), and provide a rationale.	7, 8
Information sources*	7	Describe all information sources in the search (e.g., databases with dates of coverage and contact with authors to identify additional sources), as well as the date the most recent search was executed.	7, 8
Search	8	Present the full electronic search strategy for at least 1 database, including any limits used, such that it could be repeated.	7, Tables A.1 to A.5 in Supplementary Data
Selection of sources of evidence†	9	State the process for selecting sources of evidence (i.e., screening and eligibility) included in the scoping review.	8, 9
Data charting process‡	10	Describe the methods of charting data from the included sources of evidence (e.g., calibrated forms or forms that have been tested by the team before their use, and whether data charting was done independently or in duplicate) and any processes for obtaining and confirming data from investigators.	8, 9
Data items	11	List and define all variables for which data were sought and any assumptions and simplifications made.	5, Table 1
Critical appraisal of individual sources of evidence§	12	If done, provide a rationale for conducting a critical appraisal of included sources of evidence; describe the methods used and how this information was used in any data synthesis (if appropriate).	NA
Synthesis of results	13	Describe the methods of handling and summarising the data that were charted.	9
**RESULTS**			
Selection of sources of evidence	14	Give numbers of sources of evidence screened, assessed for eligibility, and included in the review, with reasons for exclusions at each stage, ideally using a flow diagram.	10

St. Michael's
Inspired Care.
Inspiring Science.

For peer review only - http://bmjopen.bmj.com/site/about/guidelines.xhtml

SECTION	ITEM	PRISMA-ScR CHECKLIST ITEM	REPORTED ON PAGE #
Characteristics of sources of evidence	15	For each source of evidence, present characteristics for which data were charted and provide the citations.	10, Tables 3 and C
Critical appraisal within sources of evidence	16	If done, present data on critical appraisal of included sources of evidence (see item 12).	NA
Results of individual sources of evidence	17	For each included source of evidence, present the relevant data that were charted that relate to the review questions and objectives.	10-20, Table 3 and Table C in Supplementary Data
Synthesis of results	18	Summarise and/or present the charting results as they relate to the review questions and objectives.	10-20, Table 3 and Table C in Supplementary Data
**DISCUSSION**			
Summary of evidence	19	Summarise the main results (including an overview of concepts, themes, and types of evidence available), link to the review questions and objectives, and consider the relevance to key groups.	20, 21
Limitations	20	Discuss the limitations of the scoping review process.	22, 23
Conclusions	21	Provide a general interpretation of the results with respect to the review questions and objectives, as well as potential implications and/or next steps.	23
**FUNDING**			
Funding	22	Describe sources of funding for the included sources of evidence, as well as sources of funding for the scoping review. Describe the role of the funders of the scoping review.	24

JBI = Joanna Briggs Institute; PRISMA-ScR = Preferred Reporting Items for Systematic reviews and Meta-Analyses extension for Scoping Reviews; NA = Not Available.
* Where *sources of evidence* (see second footnote) are compiled from, such as bibliographic databases, social media platforms, and Web sites.
† A more inclusive/heterogeneous term used to account for the different types of evidence or data sources (e.g., quantitative and/or qualitative research, expert opinion, and policy documents) that may be eligible in a scoping review as opposed to only studies. This is not to be confused with *information sources* (see first footnote).
‡ The frameworks by Arksey and O'Malley (6) and Levac and colleagues (7) and the JBI guidance (4, 5) refer to the process of data extraction in a scoping review as data charting.
§ The process of systematically examining research evidence to assess its validity, results, and relevance before using it to inform a decision. This term is used for items 12 and 19 instead of "risk of bias" (which is more applicable to systematic reviews of interventions) to include and acknowledge the various sources of evidence that may be used in a scoping review (e.g., quantitative and/or qualitative research, expert opinion, and policy document).

Reference*:* Tricco AC, Lillie E, Zarin W, O'Brien KK, Colquhoun H, Levac D, et al. PRISMA Extension for Scoping Reviews (PRISMAScR): Checklist and Explanation. Ann Intern Med. 2018;169:467–473. http://doi.org/10.7326/M18-0850.

St. Michael's
Inspired Care.
Inspiring Science.

BMJ Open

Costs, consequences and value for money in non-medical prescribing: A scoping review

Journal:	*BMJ Open*
Manuscript ID	bmjopen-2022-067907.R1
Article Type:	Original research
Date Submitted by the Author:	27-Oct-2022
Complete List of Authors:	Babashahi, Saeideh; University of Sussex, Global Health and Infection<br>Carey, Nicola; University of the Highlands and Islands, Department of Nursing and Midwifery<br>Jani, Yogini; Imperial College London, Pharmacy Department<br>Hart, Kath; University of Surrey, Department of Nutritional Sciences<br>Hounsome, Natalia; Brighton and Sussex Medical School, Centre for Global Health Research
**Primary Subject Heading**:	Health economics
Secondary Subject Heading:	Global health, Health policy, Health services research
Keywords:	Health economics < HEALTH SERVICES ADMINISTRATION & MANAGEMENT, Health policy < HEALTH SERVICES ADMINISTRATION & MANAGEMENT, PUBLIC HEALTH

SCHOLARONE™
Manuscripts

I, the Submitting Author has the right to grant and does grant on behalf of all authors of the Work (as defined in the below author licence), an exclusive licence and/or a non-exclusive licence for contributions from authors who are: i) UK Crown employees; ii) where BMJ has agreed a CC-BY licence shall apply, and/or iii) in accordance with the terms applicable for US Federal Government officers or employees acting as part of their official duties; on a worldwide, perpetual, irrevocable, royalty-free basis to BMJ Publishing Group Ltd ("BMJ") its licensees and where the relevant Journal is co-owned by BMJ to the co-owners of the Journal, to publish the Work in this journal and any other BMJ products and to exploit all rights, as set out in our licence.

The Submitting Author accepts and understands that any supply made under these terms is made by BMJ to the Submitting Author unless you are acting as an employee on behalf of your employer or a postgraduate student of an affiliated institution which is paying any applicable article publishing charge ("APC") for Open Access articles. Where the Submitting Author wishes to make the Work available on an Open Access basis (and intends to pay the relevant APC), the terms of reuse of such Open Access shall be governed by a Creative Commons licence – details of these licences and which Creative Commons licence will apply to this Work are set out in our licence referred to above.

Other than as permitted in any relevant BMJ Author's Self Archiving Policies, I confirm this Work has not been accepted for publication elsewhere, is not being considered for publication elsewhere and does not duplicate material already published. I confirm all authors consent to publication of this Work and authorise the granting of this licence.

For peer review only

Costs, consequences and value for money in non-medical prescribing: A scoping review

Saeideh Babashahi[1,*], Nicola Carey[2], Yogini Jani[3], Kathryn Hart[4], Natalia Hounsome[5]

[1] Global Health and Infection, Brighton and Sussex Medical School, University of Sussex, Brighton, UK. Email: s.babashahi@bsms.ac.uk

[2] Department of Nursing and Midwifery, University of the Highlands and Islands, UK. Email: nicola.carey@uhi.ac.uk

[3] Centre for Medicines Optimisation Research and Education, UCLH NHS Foundation Trust and UCL School of Pharmacy, UK. Email: y.jani@ucl.ac.uk

[4] School of Biosciences & Medicine, Faculty of Health and Medical Sciences, University of Surrey Guildford, UK. Email: k.hart@surrey.ac.uk

[5] Global Health and Infection, Brighton and Sussex Medical School, University of Sussex, Brighton, UK. Email: n.ivashikina@bsms.ac.uk

* Correspondence: Saeideh Babashahi, Global Health and Infection, Brighton and Sussex Medical School, University of Sussex, Brighton, UK. Email: s.babashahi@bsms.ac.uk

Word count: 3890

For peer review only

For peer review only - http://bmjopen.bmj.com/site/about/guidelines.xhtml

ABSTRACT

Objectives: Non-medical prescribing (NMP) is a key feature of the UK healthcare system that refers to the legal prescribing rights granted to nurses, pharmacists and other non-medical healthcare professionals who have completed an approved training programme. NMP is deemed to facilitate better patient care and timely access to medicine. The aim of this scoping review is to identify, synthesise and report the evidence on the costs, consequences and value for money of NMP provided by non-medical healthcare professionals.

Design: Scoping review

Data sources: MEDLINE, Cochrane Library, Scopus, PubMed, ISI Web of Science and Google Scholar were systematically searched from 1999 to 2021.

Eligibility criteria: Peer-reviewed and grey literature written in English were included. The research was limited to original studies evaluating economic values only or both consequences and costs of NMP.

Data extraction and synthesis: The identified studies were screened independently by two reviewers for final inclusion. The results were reported in tabular form and descriptively.

Results: A total of 420 records were identified. Of these, nine studies evaluating and comparing NMP with patient group discussions, GP-led usual care, or services provided by non-prescribing colleagues were included. All studies evaluated the costs and economic values of prescribing services by non-medical prescribers, and eight assessed patient, health or clinical outcomes. Three studies showed pharmacist prescribing was superior in all outcomes and cost-saving at large scale. Others reported similar results in most health and patient outcomes across other non-medical prescribers and control groups. NMP was deemed resource-intensive to both providers and other groups of non-medical prescribers (e.g. nurses, physiotherapists, podiatrists).

Conclusions: The review demonstrated the need for quality evidence from more rigorous methodological studies examining all relevant costs and consequences to show value for money in NMP and inform the commissioning of NMP for different groups of healthcare professionals.

[Word count: 297]

Keywords: Non-medical prescribing (NMP), non-medical prescribers, health and non-health outcomes, economic impacts, cost-effectiveness, consequences, value for money

For peer review only - http://bmjopen.bmj.com/site/about/guidelines.xhtml

Strengths and limitations of this study

1) This scoping review addresses an under-researched area to provide evidence on resource use and consequences (e.g. service improvement, patient satisfaction, waiting times, safety, etc) of non-medical prescribing (NMP) from a large body of peer-reviewed and grey literature.

2) The review was limited to original studies that evaluated the economic impacts only or both costs and consequences of NMP.

3) Drawing conclusions on the cost-effectiveness and value for money in NMP remains difficult as the existing literature is heterogenous with significant variation in participants, NMP types, comparators, study designs, costs and consequences evaluated.

For peer review only

For peer review only - http://bmjopen.bmj.com/site/about/guidelines.xhtml

INTRODUCTION

Doctors have traditionally been authorised as the main group of healthcare professionals to prescribe medicines.[1],[2] With an increasing pace of population ageing and higher risks of chronic diseases, there is a growing demand for healthcare services and access to medicines.[3-5] Due, in part, to shortages within the medical workforce,[1],[6] the authority for other healthcare professionals, such as nurses and pharmacists, to prescribe medicines has been introduced in several countries such as the United States, United Kingdom (UK), Canada and Australia.[7-9]

Non-medical prescribing (NMP) is a key feature of the UK healthcare system that refers to the legal prescribing rights granted to nurses, pharmacists and other healthcare non-medical professionals who have completed an approved programme of education,[10-14] delivered via a variety of methods (often hybrid), including classroom teaching, one-to-one instruction, self-directed learning and e-learning.[15] NMP first emerged in the UK in 1999 for district nurses and health visitors,[2] and it came into effect for all registered nurses in 2001 and for pharmacists in 2003.[9],[16],[17] The UK has pioneered the gradual expansion of these prescribing roles to include a wider population of healthcare professionals across both primary care and secondary care.[8],[18] Since 2005 physiotherapists, podiatrists, and both diagnostic and therapeutic radiographers have been able to train to become supplementary prescribers (see Table 1 for definitions of supplementary prescribing (SP) and other terms).[11],[19] Independent prescribing (IP) rights were subsequently granted to optometrists in 2008[14],[20] and physiotherapists, podiatrists and chiropodists in 2013.[19],[21],[22] More recent changes in 2016 enabled therapeutic radiographers to train as independent prescribers,[23] and dietitians as supplementary prescribers[23] and in 2019 paramedics were awarded both IP and SP rights (see Table 1 for a glossary of terms).

For peer review only - http://bmjopen.bmj.com/site/about/guidelines.xhtml

Table 1. Definition of main terms (and variables) used in the study

Terminology	Definition
NMP	NMP is a term widely used in the UK, and it represents the prescribing authorities given to certain non-medical healthcare professionals (e.g. nurses, dietitians, physiotherapists) after completing a prescribing training course.[9],[15],[24]
IP	Those using IP are responsible for assessing patients' health conditions and making decisions about patients' treatment and clinical management, including prescribing, within their scope of practice.[9],[15],[24]
SP	Using SP, the initial assessment and diagnosis of a patient's condition are carried out by an independent prescriber (i.e. a GP or dentist), and the clinical condition is managed using a patient-specific clinical management plan agreed by the independent prescriber, supplementary prescriber and patient.[9],[15],[24]
PGD	PGD is a legal written framework that allows registered healthcare professionals to supply and/or prescribe specified medicines to a pre-defined group of patients without them having to see a medical prescriber (e.g. a GP).[7],[25]
Medicine management or prescribing activities	A system of processes that determines how medicines are used by patients and health providers. For the purposes of this study, medicine management and prescribing activities refer to prescribing and/or the process of giving advice about medicines and the supply of medicines, as described in the research questions sub-section.[18],[21]
Cost and resource use	This refers to the direct and/or indirect medical and/or non-medical resources consumed by the study population and/or the costs associated with setting up and implementing the intervention(s) under study.[26]
Consequence	This refers to the health, non-health, clinical and patient outcomes representing the effects of the intervention(s) under study.[26]
Perspective	This refers to (one or more groups of) stakeholders' viewpoints from which economic evaluation or cost analysis is conducted.[26] Examples include the patient perspective, societal perspective or healthcare provider perspective.
Comparator	This refers to the alternative courses of action (e.g. usual care) against which the intervention under study (e.g. NMP, the subject of this study) is evaluated.[26]

Note. NMP: non-medical prescribing; IP: independent prescribing; SP: supplementary prescribing; PGD: patient group direction; GP: general practitioner

For peer review only

Reviews of NMP developments and its benefits in the UK and other countries have been reported by others.[25],[27],[10] Although NMP is embedded within UK healthcare delivery in primary and secondary care, there is still a lack of evidence regarding its value for money.[16],[21],[28-31] Building on an earlier review by Noblet et al. (2018) on the effectiveness and cost-effectiveness of NMP from three randomised controlled trials (RCTs),[28] this scoping review aimed to assess a wider body of literature, including both peer-reviewed and grey

For peer review only - http://bmjopen.bmj.com/site/about/guidelines.xhtml

literature, to identify evidence on costs and consequences and the value for money of non-medical prescribing.

METHODS

The scoping review protocol was registered with the Open Science Framework Registry on 31 July 2021 (registered DOI: 10.17605/OSF.IO/PSR3N, accessible from https://osf.io/psr3n). We followed the Preferred Reporting Items for Systematic Reviews and Meta-Analysis extension for Scoping Reviews (PRISMA-ScR) reporting guideline recommended by Tricco et al. (2018) to report our scoping review study.[32] This scoping review was conducted using the five-stage methodological framework developed by Arksey and O'Malley and further developed by Levac et al. and the Joanna Briggs Institute to ensure rigour in reporting the review and its methodology.[33-35] The five stages are outlined below:

Stage 1: identifying the research questions

1) What types of prescribing practices (e.g. SP, IP) have been implemented and evaluated across eligible groups of healthcare professions (e.g. pharmacists, podiatrists, dietitians, etc) in different studies?

2) What measures and tools have been used to evaluate the economic values, safety, effectiveness and other consequences of prescribing by non-medical prescribers in various settings?

3) What are relevant costs, resource use, health, non-health and clinical outcomes associated with services provided by non-medical prescribers in both peer-reviewed and grey literature?

For peer review only - http://bmjopen.bmj.com/site/about/guidelines.xhtml

Stage 2: search strategy and screening

The scope and practice of NMPs vary globally.[7],[18],[30],[36],[37] For the purposes of this review, NMP was assumed to include medicine management activities that are legally and technically considered prescribing and provided by healthcare professionals who are eligible to prescribe and have completed an approved programme of education. Consistent with Courtenay et al. (2012) and Carey et al. (2020), these medicine management activities include “making recommendations for patients to buy medicine(s) over the counter; amending prescribed medication; medication review; written recommendation to GP; recommending in patients’ hospital notes; prescribing via hospital medication charts; patient group directions; remote prescribing via telephone, email and fax; issuing hospital-specific prescription; signing issued prescription via GP repeat prescribing system; issuing private prescription directly to the patient”.[21],[30]

A comprehensive search strategy was developed by the research team to enable a stepwise search process. Based on initial exploratory research, we included grey literature and journal and conference articles with full-text written in English from 1999 to 2021.[24] On 14 January 2022, we searched PubMed, Cochrane Database of Systematic Reviews, Web of Science, Scopus and MEDLINE databases for articles published between 1 January 1999 and 1 January 2022.

The detailed search terms and strategies for different databases are presented in Tables A.1 to A.5 in Supplementary Data. A non-systematic search in Google Scholar was performed to find the grey literature. The search terms used for Google Scholar are equivalent to those of other search engines. In brief, our search strategy included (non-medical prescrib* OR NMP OR non-doctor prescrib*) AND (pharmac* OR nurs* OR non-medical healthcare professionals OR allied health professionals OR AHPs OR diet* OR radiograph* OR midwiv* OR physiotherap* OR podiatr* OR optometr* OR paramedic*) AND (consequences OR health

For peer review only - http://bmjopen.bmj.com/site/about/guidelines.xhtml

outcomes OR non-health OR clinical outcomes OR effectiveness OR patient outcomes) AND (economic impacts OR costs OR resource use).

The scoping review included original research, RCT studies and grey literature analyses of resource use only or both consequences and costs to evaluate NMP provided by non-medical healthcare professionals. Commentaries, letters, protocols and editorials were excluded. A broad search strategy was implemented to ensure that the inclusion of studies was as comprehensive as possible. Search terms were derived from titles, abstracts, and keywords identified in key publications and from search terms used in previous reviews related to non-medical prescribing.[8],[9],[29] In addition, relevant references of included studies were checked (snowballing search).

All articles identified from the searches were transferred to the EndNote reference manager software version 20.2, and all duplicates and titles in languages other than English were removed. The PICOS (population, intervention, context, outcome, and study design) framework was used to establish eligibility criteria.[38] Table 2 provides further information regarding the inclusion criteria according to the PICOS approach.

Table 2. PICOS table describing inclusion criteria

Component	Description
Population	- Human participants (e.g. nurse, pharmacist and other non-medical prescribers, and patients with any health conditions managed by these groups)<br>- No restriction on age or gender
Intervention	- All types of non-medical prescribing (any medicine management activity that is legally and technically considered prescribing and provided by non-medical professionals)
Context	- All peer-reviewed published articles (in journals and conferences) and grey literature with full-text written in English from 1999 to 2021<br>- No restrictions on setting or country
Outcome	- Cost and consequence outcomes of NMP services provided by nurses, pharmacists and other non-medical prescribers
Study design	- Original research and clinical trials that evaluated costs and economic impacts of NMP only or both cost and consequence outcomes of NMP (peer-reviewed or grey literature)

Note. PICOS: (population, intervention, context, outcome, and study design); NMP: non-medical prescribing.

For peer review only - http://bmjopen.bmj.com/site/about/guidelines.xhtml

Stage 3: study selection

The review process included an initial screening of the title and abstract of the studies by three authors (SB, NH and NC) to assess their eligibility for full-text retrieval. Any studies that were not excluded confidently through title and abstract screening during the initial screening step were included for full-text screening. The full-text screening of the selected studies was divided between authors and carried out independently by two reviewers (SB, NH, YJ and KH). Any disagreement on selected papers was resolved through discussion among the authors. After identifying and removing duplicates, studies were excluded if (1) they were not original studies, (2) no abstract or full-text was available, (3) they were not in English, or (4) the focus of the study was outside the scope of our review (see Table 2), or (5) prescribing and medicine management activities evaluated did not meet those indicated by Courtenay et al. (2012) and Carey et al. (2020).[21],[30]

Stage 4: data extraction and analysis

Data from the articles and grey literature based on the inclusion criteria mentioned above were extracted using a bespoke data extraction form. A Microsoft Excel 2019 based form was initially developed by the first author and validated by other authors for charting the data from selected studies and reporting the variables regarding the study, participants, interventions and outcome characteristics – e.g. authors, publication year, study context and design, sample size, type of prescribing, cost and consequence outcomes measures and key findings – based on our research questions (Table 1 represents the definition of the main variables). Data extracted were checked by a second reviewer for accuracy and completeness.

Stage 5: collating and reporting the results

The PRISMA-ScR reporting checklist (Table B, Supplementary Data) was used to synthesise and report the results of our scoping review.[32] Data synthesis was undertaken by the first

For peer review only - http://bmjopen.bmj.com/site/about/guidelines.xhtml

author in consultation with the research team. The findings of selected studies were summarised and presented in tabular forms and descriptively highlighting the key research findings (e.g. economic impacts, consequences of NMP, setting, NMP type, etc) of selected studies and the existing research gaps around NMP practice.

Patient and Public Involvement

No patients or public were involved in the study.

RESULTS

Database search findings

The database search generated 420 records. A total of 236 records were removed due to duplication. Of the remaining 184 records, we excluded 171 records in the initial review of titles and abstracts as these studies were not original research evaluating NMP. For the remaining 13 records, the full-text papers were independently reviewed by two reviewers, and a further four studies were excluded because they did not report resource use and economic impacts of NMP or did not fit within our definition of prescribing and medicine management activities. Nine studies were included in the final review (eight original research studies[7],[21],[37],[39-43] and one grey literature paper[44]). Figure 1 shows the PRISMA flow chart of the included studies in our scoping review.

General characteristics of included studies

The key characteristics of included papers are summarised in Table 3 and Table C (Supplementary Data). Of the nine papers, six were from the UK,[7],[21],[37],[42-44] two from Canada,[39],[41] and one from Australia.[40] Papers were published between 2010 and 2022 and evaluated the impact of NMP practices by pharmacists (n=4),[39],[40-42] nurses (n=3),[7],[37],[43] physiotherapists and podiatrists (n=1),[21] and another estimating NMP cost-savings in primary and secondary care for a range of health professions.[44] Types of prescribing services

For peer review only - http://bmjopen.bmj.com/site/about/guidelines.xhtml

evaluated in these studies included SP (n=2)[44],[43] or IP (n=8)[7],[21],[37],[39-42],[44] and community nursing.[44]

For peer review only

For peer review only - http://bmjopen.bmj.com/site/about/guidelines.xhtml

Table 3: Overview and general characteristics of included studies

Authors (year)	Context (country/setting)	Study design	Type of prescribing	Comparator	Medicine management or prescribing activity	Study population
Black et al. (2022)[7]	UK, urban sexual health services	Mixed methods and a comparative case study (cost-consequence framework)	Nurse IP	PGD by non-prescribing nurses	Prescribed medications	N = 26 nurse prescribers<br>N = 67 PGDs users
Carey et al. (2020)[21]	UK mixed range of settings (primary and secondary care, social enterprise and private practice)	Mixed methods and a comparative case study (cost-consequence framework)	Physiotherapist IP<br>Podiatrist IP	Non-prescribing physiotherapists<br>Non-prescribing podiatrists	Prescribed and reviewed medications	N = 488 patients (243 IP sites and 245 NP sites)<br>N = 7 matched pairs of IP and NP sites (3 podiatrists and 4 physiotherapists)
Al Hamarneh et al. (2019)[39]	Canada, primary care (cardiovascular risk reduction)	Cost-effectiveness analysis (Markov model)	Pharmacist IP	Usual care	Prescribed and reviewed medications	The authors developed their model based on the population observed in the R_XEACH trial[45] as follows:<br>N = 723 patients (370 in intervention and 353 in control)<br>N = 54 pharmacies in the RCT
Hale et al. (2018)[40]	Australia, an elective surgery pre-admission clinic (venous thromboembolism)	Cost-effectiveness analysis (decision tree model)	Pharmacist IP	Usual care	Prescribed medications	The authors developed their model based on the population observed in an earlier trial[46] as follows:<br>N = 384 patients (194 in intervention and 190 in control)<br>N = 1 pharmacist prescriber<br>N = 59 medical prescribers
Marra et al. (2017)[41]	Canada, community care, hospitals, or primary care (hypertension)	Cost-effectiveness analysis (Markov model)	Pharmacist IP	Usual care	Prescribed medications	The authors developed their model based on the population observed in the R_XACRION trial[47] as follows:<br>N = 248 patients (181 in intervention and 67 in control)<br>N = 20 pharmacists practised in the community<br>N = 2 pharmacists from hospital outpatient clinics<br>N = 6 pharmacists from primary care clinics

For peer review only - http://bmjopen.bmj.com/site/about/guidelines.xhtml

Authors (year)	Context (country/setting)	Study design	Type of prescribing	Comparator	Medicine management or prescribing activity	Study population
i5 Health (2015)[44]	England, various settings (e.g. primary and secondary care)	Economic analysis of audits, self-reported questionnaires, interviews	IP and SP (for a range of professions, e.g. physiotherapists, podiatrists, midwives and radiographers)<br>Community nurse prescribers	NA	NA	Based on an estimation of the NMP practitioners registered with Northwest England NHS trusts (N = 1,566 unique prescribers)
Courtenay et al. (2015)[37]	England, primary care (type 2 diabetes)	Mixed methods and a comparative case study (cost-consequence framework)	Nurse IP	Non-prescribing nurses	Prescribed and reviewed medications, recommended decisions, provided advice, and discussed medications with GPs or colleagues	N = 12 general practices (6 prescribing nurses and 6 non-prescribing nurses)<br>N = 214 patients (131 in nurse prescriber sites and 83 in non-prescriber sites)
Neilson et al. (2015)[42]	UK, primary care (chronic pain)	Regression analysis of costs and effects; the expected value of sample information analysis	Pharmacist IP	Usual care	Prescribed and reviewed medications	N = 6 general practices<br>N = 125 patients (39 in prescribing, 44 in review and 42 in usual care arms)<br>No information is provided about the number of non-medical prescribers in the two groups
Norman et al. (2010)[43]	UK, primary care (mental health)	Cost-consequences analysis; matched post-test control study	Nurse SP	Usual care	Prescribed medicines	N = 90 patients (45 matched pairs)<br>No information is provided about the number of prescribers in the two groups

Note. PGD: patient group direction; IP: independent prescribing; NP: non-prescribing; SP: supplementary prescribing; RCT: randomised controlled trial; GP: general practitioner; NA: not available; NMP: non-medical prescribing.

For peer review only - http://bmjopen.bmj.com/site/about/guidelines.xhtml

Methodological and reporting considerations

Three out of nine papers conducted a model-based economic evaluation (i.e. cost-effectiveness analysis) using the outcomes from an earlier trial with an assessment of uncertainty (in the form of a deterministic and/or probabilistic sensitivity analysis).[39],[40],[41] Four studies conducted a cost-consequence-based approach listing costs and outcomes of NMP without assessing sources of uncertainty.[7],[21],[37],[43] A bottom-up costing approach (using detailed input data from records or questionnaires at the service provider level) was used in most studies with clear information on costs per unit.[7],[21],[37],[39-42] Overall non-model-based studies did not provide an explanation of sample size sufficiency. Only one study suggested that determining an optimal and larger sample size would be required to draw a precise and accurate conclusion.[42] Two studies failed to specify the number or characteristics of the study participants (e.g. non-medical prescribers or patients).[42],[43]

Measures of costs

The resource use and costs evaluated in the included studies fall into the following three main categories:

i) Prescribing training course

Four out of nine articles applied direct costs associated with prescribing training and NMP courses (e.g. training course fee, supervision time, employer-paid study time),[7],[39],[41],[42] with one study using time-off-work to complete the course.[43] Other relevant expenses such as out-of-pocket expenses (e.g. travel, accommodation, etc) by qualified non-medical prescribers and their (unpaid) personal study time were included in two studies.[7],[43]

ii) Prescription (and consultation)

Expenses applied in this category by some studies included tests and other relevant services, referrals to other healthcare professionals, frequency of follow-up, time spent preparing for a

For peer review only

For peer review only - http://bmjopen.bmj.com/site/about/guidelines.xhtml

prescribing consultation, time taken to prescribe, review or complete the medication plan for a patient, and the number of patients prescribed for, consultation frequency, time spent discussing the patient and obtaining prescriptions or clinical advice sought from GPs or other NMP practitioners, unplanned consultations for the health condition after the index consultation and frequency of new medications.[7],[21],[37],[39],[40],[42],[43] Incorrect or over-prescribing was identified and considered as an indication of wastage, and the 'wasted' medication, as well as under-prescribed medicine that should have been prescribed, were considered as another source of cost.[7],[43]

iii) Other relevant expenses

Some studies also considered the expenses associated with service utilisation, e.g. hospital admissions, outpatient expenses, inpatient days and A&E visits,[21],[37],[41-43] or the healthcare and medical costs associated with targeted health conditions across case and control groups.[39],[40],[41]

Measures of outcome

Health-related quality of life was one of the main health outcomes evaluated using EQ-5D or SF-6D questionnaires,[21],[39],[40-42] and the benefits to patients of appropriate prescribing were measured in terms of increased quality-adjusted life years (QALYs) in some of these studies.[39],[40-42] Multiple studies evaluated patient experience and satisfaction as one of the main patient outcomes.[7],[21],[37],[43] Life years gained were applied by two studies.[39],[41] Medicine adherence and ease of access to services were other outcomes reported in one study.[21] Examples of specific clinical and health outcomes used in the studies were self-care and relevant clinical indicators such as HbA1c test results (mean blood sugar level) and body mass index for patients with diabetes,[37] the reduced risk of the disease under study (e.g. venous thromboembolism, cardiovascular disease)[39],[40],[41] and reduced blood pressure.[41]

For peer review only - http://bmjopen.bmj.com/site/about/guidelines.xhtml

Key findings: the costs and consequences of NMP

A summary of the key cost and consequence findings is provided for pharmacists, nurses and other non-medical prescribers in turn (see Table C in Supplementary Data for detailed information).

Pharmacists

The NMP practices by pharmacists were evaluated across a range of health conditions (e.g. venous thromboembolism, hypertension, etc), and significant improvements in health and clinical outcomes were reported at the end of the observation in three studies.[39],[40],[41] As such, Marra et al. (2017) found the 30-year risk of cardiovascular disease (CVD) in the pharmacist prescriber group was reduced from 0.61 in base-case to 0.41 (indicating a reduction of two CVD events in every 10 individuals receiving the intervention).[41] Although the intervention was associated with increased costs of CA$7,145 due to the intervention itself and medications, this was compensated for by a reduction of CA$15,094 in CVD and other co-morbidities costs, suggesting pharmacist independent prescribing was less costly and more effective than usual care.[41]

Consistent with Marra et al. (2017), two other studies – i.e. Al Hamarneh et al. (2019) and Hale et al. (2018) – reported that pharmacist prescribing was cost-effective and cost-saving for patients with CVD and venous thromboembolism, respectively.[39],[40] Only Neilson et al. (2015) found that, relative to the usual-care arm, pharmacist prescribing for chronic pain was more costly (£77.5 for prescribing and £54.4 for review arms) and provided similar QALYs. Neilson et al. recruited a total sample of 125 patients in this RCT, but the authors recommended a larger sample size (between 460–690 for a threshold of £30,000 QALY gained or 540–780 for a threshold of £20,000 QALY gained) according to an expected value of sample information analysis (indicating that additional information collected from a larger sample will reduce uncertainty and provide more reliable data).[42]

For peer review only - http://bmjopen.bmj.com/site/about/guidelines.xhtml

Nurses

Norman et al. (2010) indicated that patients in the mental health nurse prescriber group had a significantly higher level of satisfaction with nurse prescribers than those in the medical prescriber group.[43] Similarly, Courtenay et al. (2015) reported that the average patient satisfaction for some specified aspects of care was significantly higher among diabetic patients in the nurse prescriber group than among those of the non-prescribing nurses.[37] Nonetheless, no significant differences were reported with respect to patients' overall satisfaction by Courtenay et al. (2015) and Black et al. (2022). Other specific or generic health and social outcomes were found to be similar among nurse prescribers and the control groups in these three studies.[7],[37],[43]

NMP was deemed resource-intensive to both providers and nurse prescribers. According to Black et al. (2022), the training-related costs included the course fee (paid fully by employers or training grants – ranging from £900 to £3,555 in 2016), an average of 20.1 of employer-paid study days for 92% of nurse prescribers (ranging from 1 to 31 funded by employer), and an average of 7.4 supervised days (ranging from 2 to 13.7 day, incurring a cost of £6,451 to the NHS for each nurse – ranging from £1,283 to £11,138) during training.[7] It is important to note that although PGDs (i.e. patient group directions, please see Table 1 for more information) provide a legal framework for health professionals to supply and administer a specified medicine to a pre-defined group of patients, and there is no mandatory training required prior to their use; there are limitations to their use, indicating that NMP might be worth the training cost.[7],[25] Employment costs of prescribing nurses were deemed potentially higher as they were on higher pay bands compared to non-prescribing nurses, including PGD users.[7],[37],[43] Consultation durations and unplanned re-consultations were similar for both sexual health nurse prescribers and medical prescribers, as reported by Black et al. (2022).[7] However, Courtenay et al. (2015) reported longer consultations for patients with diabetes managed by nurse prescribers suggesting it was more costly relative to GPs and non-prescribing nurses.[37] No statistically significant differences in prescribing new medicines or

For peer review only - http://bmjopen.bmj.com/site/about/guidelines.xhtml

use of other healthcare services between groups were identified in any of the three studies assessing this outcome.[7],[37],[43]

Other non-medical prescribers

Only one study evaluated the benefits and costs of services by physiotherapist and podiatrist independent prescribers compared to non-prescribing physiotherapists and podiatrists.[21] Carey et al. (2020) showed the level of satisfaction with consultation and services was significantly higher in both non-medical prescriber groups. Patients of physiotherapist or podiatrist independent prescribers were more likely to receive medicine information or advice during consultations (39.7%) compared to patients managed by non-prescribers (24.5%). No significant differences were reported in the quality of life in patients for all groups.[21] Consultation durations were longer for both prescriber groups, resulting in increased costs for prescribing physiotherapists (£7.95 per contact) and prescribing podiatrists (£8.62) compared to non-prescribers. No training-related costs were reported.[21]

DISCUSSION

Building on a previous systematic review[28] which included only three RCTs published before 2015, we have included a wider range of studies evaluating the consequences, resource use, costs and value for money in NMP. We used the PRISMA-ScR framework to guide the review, searched multiple databases and used snowballing techniques to improve the comprehensiveness of the study. Despite this, only one additional source of evidence from the grey literature was identified. The NMP literature has largely focused on assessing the benefits and effectiveness of prescribing authorities without evaluating the costs and resource use. Some other studies have concentrated on topics such as NMP trends and related national policies over time or implementation barriers and/or facilitators of NMP for different professions.[1],[9],[18],[24],[27],[48] This review demonstrated the lack of evidence on costs, consequences and value for money in NMP by different groups of healthcare professionals.

For peer review only - http://bmjopen.bmj.com/site/about/guidelines.xhtml

Our scoping review identified nine sources of evidence that evaluated the economic impacts, resource use and consequences of NMP. Three studies showed pharmacist prescribing was superior in all outcomes and cost-saving at large scale. Others reported similar results in most health, clinical and patient outcomes across other non-medical prescribers and control groups (e.g. GP-led usual care). NMP was deemed resource-intensive to both providers and other groups of non-medical prescribers (e.g. nurses, physiotherapists, podiatrists).

This scoping review revealed evidence sources were heterogeneous with regard to design, setting, range of cost and consequence outcomes, NMP types and comparators. In general, the existing evidence indicates that services provided by non-medical prescribers might positively influence patients' satisfaction with care, medication and their quality of life.[21],[37],[39],[40],[41] However, some of these findings came from non-RCT studies without robust evaluation of all relevant consequences and costs. Besides, some of these studies recruited small sample sizes, suggesting it is difficult to make any statement about the significance of the results beyond the sample included and therefore, these findings should be treated with caution.

The costs and consequences evidence on NMP has slowly grown since 2010 and appears to be concentrated in three countries, the UK, Canada and Australia, where prescribing rights are more developed. Despite the large increase in NMP in the UK and around the world and the increasing number of studies on NMP, there is still very limited information on the effectiveness, costs and cost-effectiveness of NMP by different professions. Many papers evaluated NMP delivered by nurses and pharmacists using various sources of costs, health, clinical and patient outcomes with varied comparators for a range of health conditions, which limits their generalisability and usefulness for other settings and professions. Only three studies conducted a cost-effectiveness analysis to evaluate and demonstrate value for money in pharmacist prescribing.[9],[39],[41] Other studies used a cost-consequence approach (CCA) that provided disaggregated costs and outcomes of NMP for nurses, physiotherapists and podiatrists.[7],[21],[37] Although CCA helps identify and list relevant costs and outcomes

For peer review only - http://bmjopen.bmj.com/site/about/guidelines.xhtml

associated with the interventions, it does not provide a definitive cost-outcome ratio and definite cost-effectiveness results for the interventions under study.[26]

The number of studies, particularly economic evaluation studies, assessing the economic burden and effectiveness of NMP has been increasing, but there is still a dearth of evidence on the cost-effectiveness and value for money in NMP authorities by recently awarded non-medical prescribers such as radiographers and dietitians. As most cost-effectiveness evidence relates to pharmacists, it is important to evaluate the impact, safety, resource use and economic value of prescribing by non-medical prescribers in other professions to inform policy and practice around NMP where it provides value for money. It is also important to acknowledge and further explore the challenges related to capturing these data, as NMP has been introduced as an additional role for healthcare professions, and hence it is not easy to separate and capture some of the added costs and values in terms of these additional prescriptive authorities.[21],[28]

There seem to be research-quality gaps in the literature. Although we did not assess the quality of included studies, some of the studies have performed non-model-based analysis using small samples that might affect the analysis, and in some cases, the main outcomes and sources of costs (e.g. training-related, etc) were not included in the analysis. Despite the importance of rigour in quantitative research, sample size reporting and sufficiency assessment remained inconsistent and partial in these studies.

Strengths and limitations

A rigorous search was conducted, allowing for a diverse set of literature (from both peer-reviewed and grey) to be identified in a robust and reproducible manner. To our knowledge, this is the first scoping review covering and representing the largest and most up-to-date evidence on the costs and consequences of prescribing practices by nurses, pharmacists and other non-medical prescribers. This scoping review contributes to the discussion of the costs and consequences of NMP and the existing research gaps regarding value for money in NMP

For peer review only - http://bmjopen.bmj.com/site/about/guidelines.xhtml

for different groups of healthcare professionals. Original studies that did not report resource use and costs associated with NMP were not included in our review. While this strategy contributed to a more focused search, studies that reported only the effectiveness and benefits of NMP practices without evaluating costs are missing. Comparison of studies was challenged by heterogeneity regarding the profession, type of NMP, costs and consequences evaluated.

CONCLUSION

NMP practice is now an integrated feature of healthcare delivery in the UK and around the world, but considerable uncertainty remains regarding the costs, consequences and cost-effectiveness of the prescribing rights granted to non-medical prescribers, including therapeutic radiographers and dietitians. In order to determine accurate mean values and detect cost and benefit differences across non-medical prescribers and control groups, it is important that future studies involve larger and more representative samples with greater power. Adopting a model-based approach within each profession using targeted outcome measures would also enable a more robust comparison and improve understanding of how to best utilise NMP and healthcare professionals' skills and ensure it offers a cost-effective solution to providing faster and improved access to medicine and healthcare services for patients by the most appropriate healthcare professionals.

Figure 1. PRISMA flowchart of the study selection process

For peer review only - http://bmjopen.bmj.com/site/about/guidelines.xhtml

Acknowledgements: None.

Author Contributions: SB: Study design; formulation of search strategies; investigation, screening and review – titles, abstracts and full-texts; data extraction; writing – original draft, review & editing; NC, YJ, KH, NH: Study design; formulation of search strategies; screening and review – full-texts, data extraction; writing – review & editing. All authors read and approved the final version of the manuscript before submission.

Funding: This scoping review is conducted as part of the health economic evaluation in the TRaDiP project that was funded by the Department of Health Policy Research Programme, the National Institute for Health Research (grant number: PRP12697/86202356).

Conflict of interest: Authors declare no competing interest.

Patient and public involvement: Patients and/or the public were not involved in the design, conduct, report or dissemination plans of this scoping review.

Data availability: All data are available within the article and the supplementary materials.

Ethics statements

Ethics approval: This scoping review study does not require ethical approval as it consists of reviewing and collecting secondary data from already published papers.

Patient consent for publication: Not required.

For peer review only

REFERENCES

1. Cooper R, Bissell P, Ward P, et al. Further challenges to medical dominance? The case of nurse and pharmacist supplementary prescribing. *Health & Social Care in the Community* 2012;**16**(2):115–133. https://doi.org/10.1177/1363459310364159.

2. Cooper R, Guillaume L, Avery T, et al. Nonmedical prescribing in the United Kingdom: developments and stakeholder interests. *Journal of Ambulatory Care Management* 2008;**31**(3):244–52. https://doi.org/10.1097/01.JAC.0000324670.91153.b4.

3. World Health Organization. Ageing and health. World Health Organization, 2021. https://www.who.int/news-room/fact-sheets/detail/ageing-and-health. (Accessed 12 March 2022).

4. Maresova P, Javanmardi E, Barakovic S, et al. Consequences of chronic diseases and other limitations associated with old age - a scoping review. *BMC Public Health* 2019;**19**(1),1431. https://doi.org/10.1186/s12889-019-7762-5.

5. Kingston A, Comas-Herrera A, Jagger C, et al. Forecasting the care needs of the older population in England over the next 20 years: estimates from the Population Ageing and Care Simulation (PACSim) modelling study. *Lancet Public Health* 2018;**3**(9):e447–e455. https://doi.org/10.1016/S2468-2667(18)30118-X.

6. Nancarrow SA, Borthwick AM. Dynamic professional boundaries in the healthcare workforce. *Socioogy of Health and Illness* 2005;**27**(7):897–919. https://doi.org/10.1111/j.1467-9566.2005.00463.x.

7. Black A, Courtenay M, Norton C, et al. Independent nurse medication provision: A mixed method study assessing impact on patients' experience, processes, and costs in sexual health clinics. *Journal of Advanced Nursing* 2022;**78**(1):239–251. https://doi.org/10.1111/jan.15075.

8. Kroezen M, van Dijk L, Groenewegen PP, et al. Nurse prescribing of medicines in Western European and Anglo-Saxon countries: a systematic review of the literature. *BMC Health Services Research* 2011;**11**,127. https://doi.org/10.1186/1472-6963-11-127.

9. Graham-Clarke E, Rushton A, Noblet T, et al. Non-medical prescribing in the United Kingdom National Health Service: A systematic policy review. *PLoS One* 2019;**14**(7):e0214630. https://doi.org/10.1371/journal.pone.0214630.

10. Department of Health. The National Health System plan: A plan for investment. A plan for reform. Department of Health, 2000. http://1nj5ms2lli5hdggbe3mm7ms5.wpengine.netdna-cdn.com/files/2010/03/pnsuk1.pdf. (Accessed 12 August 2021).

11. Health and Care Professions Council. Non-medical prescribing approved courses. Health and Care Professions Council, 2021. https://www.hcpc-uk.org/education/approved-programmes/. (Accessed 10 August 2021).

12. General Pharmaceutical Council. Discussion paper on supervising pharmacist independent prescribers in training. General Pharmaceutical Council, 2016. https://www.pharmacyregulation.org/sites/default/files/discussion_paper_on_supervising_pharmacist_independent_prescribers_in_training_november_2016.pdf. (Accessed 12 March 2021).

13. Nursing and Midwifery Council. Standards for prescribing programmes. Nursing and Midwifery Council, 2018. https://www.nmc.org.uk/standards/standards-for-post-registration/standards-for-prescribers/standards-for-prescribing-programmes/. (Accessed 14 March 2021).

14. General Optical Council. Independent Prescribing. General Optical Council, 2021. https://optical.org/en/news/news-and-press-releases/goc-council-approves-updated-requirements-for-independent-prescribing-categories/. (Accessed 10 March 2021).

15. Black A, Gage H, Norton C, et al. Patient satisfaction with medication consultations and medicines information provided by nurses working autonomously in sexual health services: A questionnaire study. *Journal of Advanced Nursing* 2022;**78**(2):523–531. https://doi.org/10.1111/jan.15087.

16. Cope LC, Abuzour AS, Tully MP. Nonmedical prescribing: where are we now? *Therapeutic Advances Drug Safety* 2016;**7**(4):165–72. https://doi.org/10.1177/2042098616646726.

For peer review only - http://bmjopen.bmj.com/site/about/guidelines.xhtml

17. Department of Health and Social Security. Medicinal Products; Prescriptions by Nurses etc Act. Department of Health and Social Security, 1992. https://www.legislation.gov.uk/ukpga/1992/28/contents. (Accessed 10 February 2022).

18. Courtenay M, Carey N, Stenner K. Non medical prescribing leads views on their role and the implementation of non medical prescribing from a multi-organisational perspective. *BMC Health Services Research* 2011;**11**:142. https://doi.org/10.1186/1472-6963-11-142.

19. Allied Health Professions Federation. Outline curriculum framework for education programmes to prepare physiotherapists and podiatrists as independent/supplementary prescribers and to prepare radiographers as supplementary prescribers. Allied Health Professions Federation, 2013. http://www.ahpf.org.uk/AHP_Prescribing_Programme_Information.htm. (Accessed 10 March 2022).

20. Rumney N. Optometry and independent prescribing. *Journal of Prescribing Practice* 2019;**1**(2):87–92. https://doi.org/10.12968/jprp.2019.1.2.87.

21. Carey N, Edwards J, Otter S, et al. A comparative case study of prescribing and non-prescribing physiotherapists and podiatrists. *BMC Health Services Research* 2020;**20**(1):1074. https://doi.org/10.1186/s12913-020-05918-8.

22. Morris L. Nonmedical independent prescribing: Podiatrists to take the next step. *The Diabetic Foot Journal* 2014;**16**:146–50.

23. National Health Service. Non-medical prescribing by allied health professionals. National Health Service, 2016. https://www.england.nhs.uk/ahp/med-project/. (Accessed 10 March 2022).

24. Babashahi S, Carey N, Hart K, et al. Costs and consequences of services provided by non-medical prescribers: a scoping review protocol. *Journal of Prescribing Practice* 2022;**4**(4):160–164. https://doi.org/10.12968/jprp.2022.4.4.160.

25. Department of Health. Health Services Circular (HSC 2000/026): Patient Group Directions. Department of Health, 2000. http://webar chive.natio nalarchives.gov.uk/20130 10710 5354/http:/www.dh.gov.uk/prod_consum_dh/group s/dh_digit alass ets/@dh/@en/docum ents/digit alass et/dh_40122 60.pdf. (Accessed 15 July 2021).

26. Drummond M, Sculpher M, Torrance G. Methods for the economic evaluation of health care programmes. Oxford University Press: Oxford 2005.

27. Wilson D, Murphy J, Nam M, et al. Nurse and midwifery prescribing in Ireland: A scope-of-practice development for worldwide consideration. *Nursing and Health Sciences* 2018;**20**:264–70. https://doi.org/10.1111/nhs.12408.

28. Noblet T, Marriott J, Graham-Clarke E, et al. Clinical and cost-effectiveness of non-medical prescribing: A systematic review of randomised controlled trials. *PLoS One* 2018;**13**(3):e0193286. https://doi.org/10.1371/journal.pone.0193286.

29. Alghamdi SSA, Hodson K, Deslandes P, et al. Prescribing trends over time by non-medical independent prescribers in primary care settings across Wales (2011-2018): a secondary database analysis. *BMJ Open* 2020;**10**(10):e036379. https://doi.org/10.1136/bmjopen-2019-036379.

30. Courtenay M, Carey N, Stenner K. An overiew of non medical prescribing across one strategic health authority: a questionnaire survey. *BMC Health Services Research* 2012;**12**:138. https://doi.org/10.1186/1472-6963-12-138.

31. Graham-Clarke E, Rushton A, Marriott J. A Delphi study to explore and gain consensus regarding the most important barriers and facilitators affecting physiotherapist and pharmacist non-medical prescribing. *PLoS One* 2021;**16**(2):e0246273. https://doi.org/10.1371/journal.pone.0246273.

32. Tricco AC, Lillie E, Zarin W, et al. PRISMA Extension for Scoping Reviews (PRISMA-ScR): Checklist and Explanation. *Annals of Internal Medicine* 2018;**169**(7):467–473. https://doi.org/10.7326/M18-0850.

33. Arksey H, O'Malley L. Scoping studies: towards a methodological framework. *International Journal of Social Research Methodology* 2005;**8**:19–32. https://doi.org/10.1080/1364557032000119616.

For peer review only - http://bmjopen.bmj.com/site/about/guidelines.xhtml

For peer review only

34. Levac D, Colquhoun H, O'Brien K. Scoping studies: advancing the methodology. *Implementation Science* 2010;**5**(1):1–9. https://doi.org/10.1186/1748-5908-5-69.

35. Joanna Briggs Institute. The Joanna Briggs Institute Reviewers' Manual 2015: Methodology for JBI Scoping Reviews, 2015. The Joanna Briggs Institute. www.joannabriggs.org. (Accessed 15 July 2021).

36. Gielen SC, Dekker J, Francke AL, et al. The effects of nurse prescribing: a systematic review. *International Journal of Nursing Studies* 2014;**51**(7):1048–1061. https://doi.org/10.1016/j.ijnurstu.2013.12.003.

37. Courtenay M, Carey N, Gage H, et al. A comparison of prescribing and non-prescribing nurses in the management of people with diabetes. *Journal of Advanced Nursing* 2015;**71**(12):2950–2964. https://doi.org/10.1111/jan.12757.

38. Petticrew M, Roberts H. Systematic reviews in the social sciences: a practical guide. John Wiley & Sons 2008:79–246. https://doi.org/10.1002/9780470754887.

39. Al Hamarneh YN, Johnston K, Marra CA, et al. Pharmacist prescribing and care improves cardiovascular risk, but is it cost-effective? A cost-effectiveness analysis of the RxEACH study. *Canadian Pharmacists Journal* 2019;**152**(4):257–266. https://doi.org/10.1177/1715163519851822.

40. Hale A, Merlo G, Nissen L, et al. Cost-effectiveness analysis of doctor-pharmacist collaborative prescribing for venous thromboembolism in high risk surgical patients. *BMC Health Services Research* 2018;**18**(1):749. https://doi.org/10.1186/s12913-018-3557-0.

41. Marra C, Johnston K, Santschi V, et al. Cost-effectiveness of pharmacist care for managing hypertension in Canada. *Canadian Pharmacists Journal* 2017;**150**(3):184–197. https://doi.org/10.1177/1715163517701109.

42. Neilson AR, Bruhn H, Bond CM, et al. Pharmacist-led management of chronic pain in primary care: costs and benefits in a pilot randomised controlled trial. *BMJ Open* 2015;**5**(4):e006874. https://doi.org/10.1136/bmjopen-2014-006874.

43. Norman IJ, Coster S, McCrone P, et al. A comparison of the clinical effectiveness and costs of mental health nurse supplementary prescribing and independent medical prescribing: a post-test control group study. *BMC Health Services Research* 2010;**10**:4. https://doi.org/10.1186/1472-6963-10-4.

44. i5 Health. Non-medical prescribing (NMP): An economic evaluation. National Health System: Health Education North West 2015. http://www.i5health.com/NMP/NMPEconomicEvaluation.pdf.

45. Tsuyuki RT, Al Hamarneh YN, Jones CA, et al. The Effectiveness of Pharmacist Interventions on Cardiovascular Risk: The Multicenter Randomized Controlled RxEACH Trial. *Journal of American College of Cardiology* 2016;**67**(24):2846–2854. https://doi.org/10.1016/j.jacc.2016.03.528.

46. Hale AR, Coombes ID, Stokes J, et al. Perioperative medication management: expanding the role of the preadmission clinic pharmacist in a single centre, randomised controlled trial of collaborative prescribing. *BMJ Open* 2013;**3**(7):e003027. https://doi.org/10.1136/bmjopen-2013-003027.

47. Tsuyuki RT, Houle SK, Charrois TL, et al. Randomized Trial of the Effect of Pharmacist Prescribing on Improving Blood Pressure in the Community: The Alberta Clinical Trial in Optimizing Hypertension (RxACTION). *Circulation* 2015;**132**(2):93–100. https://doi.org/10.1161/CIRCULATIONAHA.115.015464.

48. Edwards J, Coward M, Carey N. Barriers and facilitators to implementation of non-medical independent prescribing in primary care in the UK: a qualitative systematic review. BMJ Open 2022;**12**(6):e052227. https://doi.org/10.1136/ bmjopen-2021-052227.

For peer review only

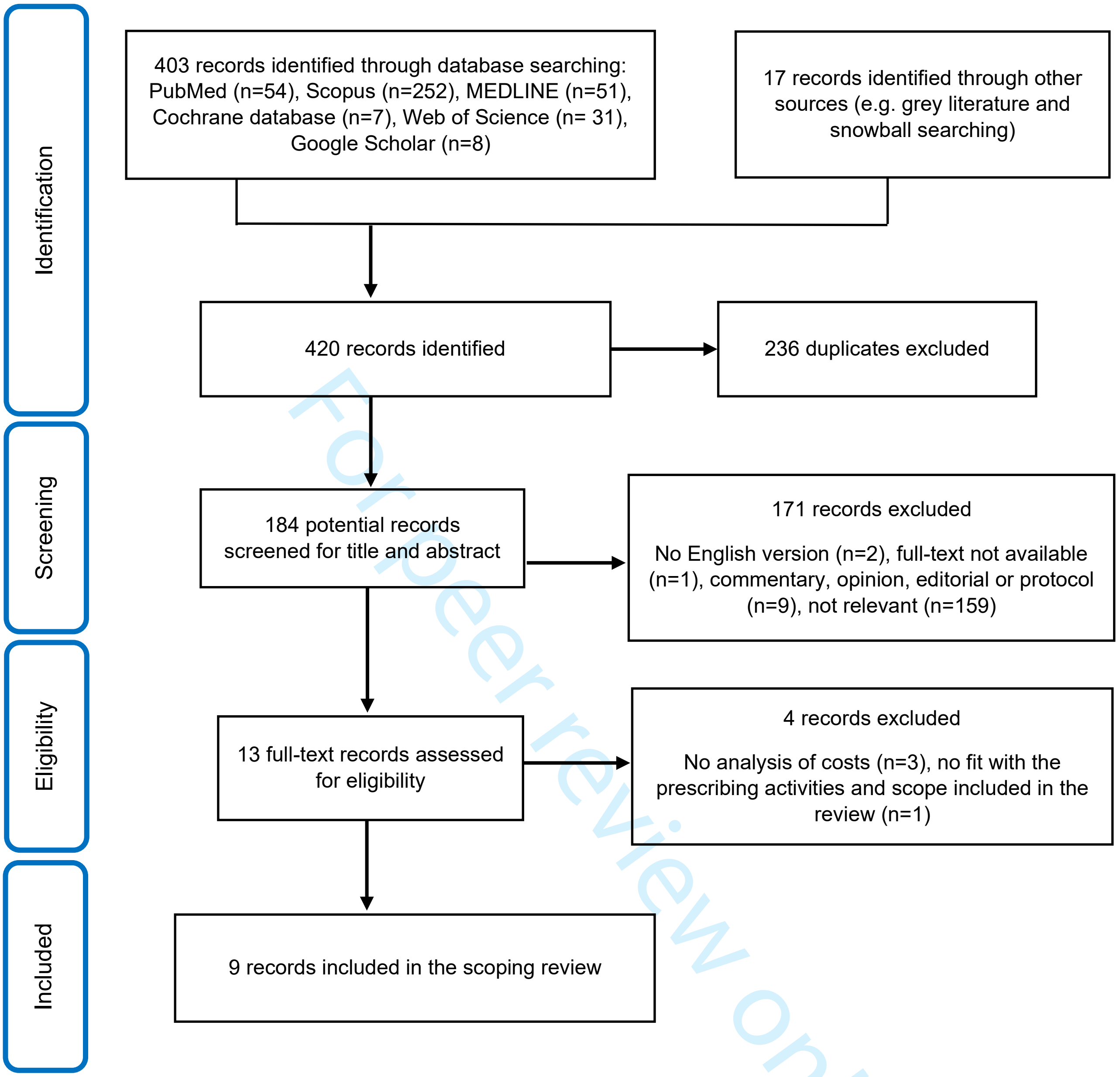

Figure 1. PRISMA flowchart of the study selection process

For peer review only

Costs, consequences and value for money in non-medical prescribing: A scoping review

Supplementary Data

Table A.1 Search terms used in PubMed from 1 January 1999 to 1 January 2022

**Database**: PubMed		
**Date of search**: 14 January 2022		
**Search step**	**Search terms and queries**	**Results**
S1	"non-medical prescribing" OR "non-medical prescriber" OR "non-medical prescribers" OR "non-doctor prescribing" OR "non-doctor prescriber" OR "non-doctor prescribers" [All Fields]	216
S2	"non-medical prescribing" OR "non-medical prescriber" OR "non-medical prescribers" OR "non-doctor prescribing" OR "non-doctor prescriber" OR "non-doctor prescribers" [All Fields] Filters: from 1999/1/1 - 2022/1/1	215
S3	"medicine management" AND activities [All Fields] Filters: from 1999/1/1 - 2022/1/1	47
S4	S2 OR S3	262
S5	(outcome OR consequence OR effectiveness OR impact) AND (economic OR cost) [All Fields] Filters: from 1999/1/1 - 2022/1/1	555,862
S6	S4 AND S5	28
S7	"allied health profession" OR "allied health professions" OR "allied health professional" OR "allied health professionals" OR "non-medical profession" OR "non-medical professions" OR “non-medical professional" OR "non-medical professionals" OR nurse OR pharmacist OR dietitian OR dietician OR radiographer OR podiatrist OR physiotherapist OR podiatrist OR optometrist OR paramedic OR midwife [All Fields] Filters: from 1999/1/1 - 2022/1/1	563,041
S8	S6 AND S7	26

Table A.2 Search terms used in MEDLINE from 1 January 1999 to 1 January 2022

**Database**: MEDLINE		
**Date of search**: 14 January 2022		
**Step**	**Search terms and queries**	**Results**
S1	"non-medical prescribing" OR "non-medical prescriber" OR "non-medical prescribers" OR "non-doctor prescribing" OR "non-doctor prescriber" OR "non-doctor prescribers" OR "medicine management activities" in Anywhere	212
S2	"non-medical prescribing" OR "non-medical prescriber" OR "non-medical prescribers" OR "medicine management activities" in Anywhere Narrowed by: Entered date: 1999-01-01 to 2022-01-01	211

For peer review only - http://bmjopen.bmj.com/site/about/guidelines.xhtml

S3	(outcome OR consequence OR effectiveness OR impact) AND (economic OR cost) in Anywhere	356,562
S4	S2 AND S3	26
S5	"allied health profession" OR "allied health professions" OR "allied health professional" OR "allied health professionals" OR "non-medical profession" OR "non-medical professions" OR “non-medical professional" OR "non-medical professionals" OR nurse OR pharmacist OR dietitian OR radiographer OR podiatrist OR physiotherapist OR podiatrist OR optometrist OR paramedic OR midwife in Anywhere	591,003
S6	S5 AND S4	25

Table A.3 Search terms used in Scopus from 1 January 1999 to 1 January 2022

**Database**: Scopus		
**Date of search**: 14 January 2022		
**Step**	**Search terms and queries**	**Results**
S1	ALL ("non-medical prescribing" OR "non-medical prescriber" OR "non-medical prescribers" OR "non-doctor prescribing" OR "non-doctor prescriber" OR "non-doctor prescribers") AND PUBYEAR > 1998	830
S2	ALL ("medicine management activities") AND PUBYEAR > 1998	11
S3	S1 OR S2	835
S4	ALL ((outcome OR consequence OR effectiveness OR impact) AND (economic OR cost)) AND PUBYEAR > 1998	4,456,222
S5	S3 AND S4	266
S6	ALL ("allied health profession" OR "allied health professions" OR "allied health professional" OR "allied health professionals" OR "non-medical profession" OR "non-medical professions" OR "non-medical professional" OR "non-medical professionals" OR nurse OR pharmacist OR dietitian OR radiographer OR podiatrist OR physiotherapist OR podiatrist OR optometrist OR paramedic OR midwife) AND PUBYEAR > 1998	1,091,538
S7	S5 AND S6	252

Table A.4 Search terms used in Web of Science from 1 January 1999 to 1 January 2022

**Database**: Web of Science		
**Date of search**: 14 January 2022		
**Step**	**Search terms and queries**	**Results**
S1	ALL=("non-medical prescribing" OR "non-medical prescriber" OR "non-medical prescribers" OR "non-doctor prescribing" OR "non-doctor prescriber" OR "non-doctor prescribers" OR "medicine management activities") Timespan: 1999-01-01 to 2022-01-01 (Publication date)	186
S2	ALL=(cost or economic) AND ALL=(outcome OR consequence OR impact OR effectiveness)	825,022
S3	S1 AND S2	17
S4	ALL=("allied health profession" OR "allied health professions" OR "allied health professional" OR "allied health professionals" OR "non-medical	407,152

For peer review only

For peer review only - http://bmjopen.bmj.com/site/about/guidelines.xhtml

	profession" OR "non-medical professions" OR “non-medical professional" OR "non-medical professionals" OR dietitian OR dietician OR radiographer OR therapist OR podiatrist OR physiotherapist OR paramedic OR optometrist OR midwife OR nurse OR pharmacist) Timespan: 1999-01-01 to 2022-01-01 (Publication date)	
S5	S3 AND S4	14

Table A.5 Search terms used in Cochrane Database of Systematic Reviews from 1 January 1999 to 1 January 2022

**Database**: Cochrane Database of Systematic Reviews		
**Date of search**: 14 January 2022		
**Step**	**Search terms and queries**	**Results**
S1	"non-medical prescribing" OR "non-medical prescriber" OR "non-medical prescribers" OR "non-doctor prescribing" OR "non-doctor prescriber" OR "medicine management activities" in All Texts	7
S2	(outcome OR effectiveness OR consequence OR impact) AND (cost or economic) in All Texts	5,675
S3	S1 AND S2	7

For peer review only - http://bmjopen.bmj.com/site/about/guidelines.xhtml
For peer review only

Costs, consequences and value for money in non-medical prescribing: A scoping review

Table B. Preferred Reporting Items for Systematic reviews and Meta-Analyses extension for Scoping Reviews (PRISMA-ScR) Checklist

SECTION	ITEM	PRISMA-ScR CHECKLIST ITEM	REPORTED ON PAGE #
**TITLE**			
Title	1	Identify the report as a scoping review.	1
**ABSTRACT**			
Structured summary	2	Provide a structured summary that includes (as applicable): background, objectives, eligibility criteria, sources of evidence, charting methods, results, and conclusions that relate to the review questions and objectives.	2
**INTRODUCTION**			
Rationale	3	Describe the rationale for the review in the context of what is already known. Explain why the review questions/objectives lend themselves to a scoping review approach.	5
Objectives	4	Provide an explicit statement of the questions and objectives being addressed with reference to their key elements (e.g., population or participants, concepts, and context) or other relevant key elements used to conceptualise the review questions and/or objectives.	4, 5-8
**METHODS**			
Protocol and registration	5	Indicate whether a review protocol exists; state if and where it can be accessed (e.g., a Web address); and if available, provide registration information, including the registration number.	6
Eligibility criteria	6	Specify characteristics of the sources of evidence used as eligibility criteria (e.g., years considered, language, and publication status), and provide a rationale.	7, 8
Information sources*	7	Describe all information sources in the search (e.g., databases with dates of coverage and contact with authors to identify additional sources), as well as the date the most recent search was executed.	7, 8
Search	8	Present the full electronic search strategy for at least 1 database, including any limits used, such that it could be repeated.	7, Tables A.1 to A.5 in Supplementary Data
Selection of sources of evidence†	9	State the process for selecting sources of evidence (i.e., screening and eligibility) included in the scoping review.	8, 9
Data charting process‡	10	Describe the methods of charting data from the included sources of evidence (e.g., calibrated forms or forms that have been tested by the team before their use, and whether data charting was done independently or in duplicate) and any processes for obtaining and confirming data from investigators.	8, 9
Data items	11	List and define all variables for which data were sought and any assumptions and simplifications made.	5, Table 1
Critical appraisal of individual sources of evidence§	12	If done, provide a rationale for conducting a critical appraisal of included sources of evidence; describe the methods used and how this information was used in any data synthesis (if appropriate).	NA
Synthesis of results	13	Describe the methods of handling and summarising the data that were charted.	9
**RESULTS**			
Selection of sources of evidence	14	Give numbers of sources of evidence screened, assessed for eligibility, and included in the review, with reasons for exclusions at each stage, ideally using a flow diagram.	10

For peer review only - http://bmjopen.bmj.com/site/about/guidelines.xhtml

SECTION	ITEM	PRISMA-ScR CHECKLIST ITEM	REPORTED ON PAGE #
Characteristics of sources of evidence	15	For each source of evidence, present characteristics for which data were charted and provide the citations.	10, Table 3 and Table C in Supplementary Data
Critical appraisal within sources of evidence	16	If done, present data on critical appraisal of included sources of evidence (see item 12).	NA
Results of individual sources of evidence	17	For each included source of evidence, present the relevant data that were charted that relate to the review questions and objectives.	10-20, Table 3 and Table C in Supplementary Data
Synthesis of results	18	Summarise and/or present the charting results as they relate to the review questions and objectives.	10-20, Table 3 and Table C in Supplementary Data
**DISCUSSION**			
Summary of evidence	19	Summarise the main results (including an overview of concepts, themes, and types of evidence available), link to the review questions and objectives, and consider the relevance to key groups.	20, 21
Limitations	20	Discuss the limitations of the scoping review process.	22, 23
Conclusions	21	Provide a general interpretation of the results with respect to the review questions and objectives, as well as potential implications and/or next steps.	23
**FUNDING**			
Funding	22	Describe sources of funding for the included sources of evidence, as well as sources of funding for the scoping review. Describe the role of the funders of the scoping review.	24

JBI = Joanna Briggs Institute; PRISMA-ScR = Preferred Reporting Items for Systematic reviews and Meta-Analyses extension for Scoping Reviews; NA = Not Available.
* Where *sources of evidence* (see second footnote) are compiled from, such as bibliographic databases, social media platforms, and Web sites.
† A more inclusive/heterogeneous term used to account for the different types of evidence or data sources (e.g., quantitative and/or qualitative research, expert opinion, and policy documents) that may be eligible in a scoping review as opposed to only studies. This is not to be confused with *information sources* (see first footnote).
‡ The frameworks by Arksey and O'Malley and Levac and colleagues and the JBI guidance refer to the process of data extraction in a scoping review as data charting.
§ The process of systematically examining research evidence to assess its validity, results, and relevance before using it to inform a decision. This term is used for items 12 and 19 instead of "risk of bias" (which is more applicable to systematic reviews of interventions) to include and acknowledge the various sources of evidence that may be used in a scoping review (e.g., quantitative and/or qualitative research, expert opinion, and policy document).

Reference: Tricco AC, Lillie E, Zarin W, O'Brien KK, Colquhoun H, Levac D, et al. PRISMA Extension for Scoping Reviews (PRISMAScR): Checklist and Explanation. Ann Intern Med. 2018;169:467–473. http://doi.org/10.7326/M18-0850.

For peer review only - http://bmjopen.bmj.com/site/about/guidelines.xhtml

Table C: Cost and outcome measures and key findings of included studies

Authors (year)	Type of prescribing	Outcome measures	Cost and resource use measures	Perspective	Study main findings
Black et al. (2022)[7]	Nurse IP	Patient consultation experience; patient satisfaction with information about medicines; medication appropriateness; medication effectiveness for the condition using the number of unplanned re-consultations in 3 months	From the NHS perspective: NMP course training and governance (staff supervision, study time and backfill); clinic processes (including medication provision, error, appropriateness, consultation duration, impact on the workload of other professionals, rates of unplanned re-consultations).<br>From nurses' perspective: study leave; personal time to study; out-of-pocket expenses for travel and purchase of learning resources for training; prospect promotions.	UK NHS, nurses and patients	***Consequences***<br>- Patient satisfaction: over 96% in both groups.<br>- Medication decisions safety: 96% for nurse prescribers vs 99% for PGD users.<br>- Medication errors: minor for both nurse prescribers (56%) and PGD users (62%), mainly documentation-related (78%), with no patients harmed.<br>- Consultation duration and unplanned re-consultations: similar for both groups. Nurse prescribers sought assistance from colleagues less frequently but provided longer consultations.<br>***Costs***<br>- NMP training fees: fully paid by their employers or health grants (£900 to £3,555) in 2016.<br>- Study days: An average of 20.1 employer-paid study days were reported by 92% of nurses, and an average of 7.4 clinically supervised days, for each nurse (an average cost of £6,45 to the NHS per nurse) during training. Eighty-one percent of nurse prescribers spent an average of 26.3 days of personal time studying for their NMP qualifications.<br>- PGD-related costs to the employer: £912 for creating a new PGD and £276 for updating.<br>- Medication costs: the average costs per patient were higher for the nurse prescriber group (£19) than for PGD users (£11.25).<br>- Nurse prescribers were in higher pay bands.
Carey et al. (2020)[21]	Physiotherapist IP<br>Podiatrist IP	Patient satisfaction; ease of access to services; quality of life	Rates of relevant tests or services ordered; frequency of new medicines; referrals to other health professionals; frequency of follow-up; consultations numbers and durations; time spent discussing the patient with other colleagues; unplanned consultations for the same condition within two months of the index consultation.	NA	***Consequences***<br>- Acceptability of independent prescribing: high (77%), with 23% preferring a GP to prescribe.<br>- Quality of life and patient satisfaction with services: significant improvements between baseline and follow-up with no significant differences in both groups.<br>***Costs***<br>- Consultation duration: An average of 6.8 minutes higher for physiotherapist prescribers and 3.5 minutes for podiatrist prescribers compared to non-prescribers.<br>- The prescriber podiatrists more frequently ordered medications and tests than non-prescribing colleagues. No cost was calculated and reported.<br>- Follow-up consultations: no significant differences in both groups.<br>- Cost of consultations: An average of £7.95 for physiotherapist prescribers and £8.62 for podiatrist prescribers higher than non-prescriber groups.<br>- No NMP training-related costs (training course, travel, supervision time, etc) were reported.

For peer review only - http://bmjopen.bmj.com/site/about/guidelines.xhtml

Authors (year)	Type of prescribing	Outcome measures	Cost and resource use measures	Perspective	Study main findings
Al Hamarneh et al. (2019)[39]	Pharmacist IP	QALYs; life years and CVD risk	Cost of baseline and follow-up visits, cost of half-day training and direct medical costs (e.g. inpatient, outpatient, medication, etc)	Canadian Medicare	***Consequences***<br>- Life years: increased by 0.11 per patient; QALYs: increased by 0.19; risk of CVD: decreased by 0.10.<br>***Costs***<br>- Medical costs: decreased by CA$2,149 compared to usual care.<br>- Cost of pharmacist intervention – 1st year: CA$233 [baseline visit: CA$125 (baseline); CA$25 (per follow-up) and cost of half-day training: CA$500 (CA$33.3 per patient)].<br>- Cost of pharmacist intervention – 2nd year: CA$175.<br>***Cost-effectiveness***<br>- Pharmacist prescribing was estimated to save more than CA$4.4 billion, add 576,689 QALYs and prevent more than 8.9 million CVD events over 30 years if applied to only 15% of the eligible patients in Canada.
Hale et al. (2018)[40]	Pharmacist IP	QALYs	Annual costs of existing and new pharmacy services; time taken by the pharmacist to prescribe (minus time saved for medical prescriber); annual salary of pharmacist (minus time costs offset by medical prescribers not having to prescribe); direct costs of acute DVT and PE to the healthcare system	Australian healthcare sector	***Consequences***<br>- QALYs: increased by 0.02 per patient in the pharmacist prescriber group.<br>- The proportion of treated VTE patients: 100% in the pharmacist prescriber and 91% in the GP groups.<br>***Costs***<br>- Cost of introducing the pharmacist IP: AU$2.24 per patient (calculated based on annual pharmacist salary and overhead costs minus GP time not required to prescribe).<br>***Cost-effectiveness***<br>- The probability of pharmacist prescribing and care being cost-effective at a willingness-to-pay of $AU40,000 was 95%.
Marra et al. (2017)[41]	Pharmacist IP	Reduced systolic blood pressure in patients with hypertension; relative risk of CVD; relative risk of renal disease; hazard ratio of mortality after CVD; life years; and HRQoL	Direct medical costs of the health conditions and the costs associated with implementing the pharmacist intervention strategy and training.	Canadian Medicare	***Consequences***<br>- Blood pressure and CVD cases: reduced by 0.21 per patient in the pharmacist prescribing group<br>- Life years: increased by 0.3 in the pharmacist prescribing group<br>- QALYs: increased by 0.4 in the pharmacist prescribing group<br>***Costs***<br>- Cost of pharmacist intervention – 1st year: CA$200 [baseline visit: CA$125 (baseline); CA$25 (per follow-up) and cost of half-day training: CA$500 (CA$33.3 per patient)].<br>- Cost of pharmacist intervention – 2nd year: CA$75.<br>- Cost of pharmacist intervention – 3rd year: CA$50.<br>***Cost-effectiveness***<br>- Pharmacist prescribing was found cost-effective and cost-saving (the reduction in costs associated with CVD and ESRD equated to cost-savings of CA$6,365 per patient in the pharmacist group over 30 years.

For peer review only - http://bmjopen.bmj.com/site/about/guidelines.xhtml

Authors (year)	Type of prescribing	Outcome measures	Cost and resource use measures	Perspective	Study main findings
i5 Health (2015)[44]	IP and SP (for a range of healthcare professions, e.g. physiotherapists, pharmacists and radiographers)<br>Community nurse prescribers	NA	Number of consultations; visits and referrals to medical prescribers avoided; lower A&E attendance and waiting times; avoided readmissions; inappropriate prescribing	NA	***Consequences***<br>- NMP practitioners provided information on patient's medications in 99% of reviews and identified inappropriate medication regimens in 50% of appointments.<br>***Costs***<br>- As shown by the i5 Health analysis of the audits for different non-medical prescriber groups in primary and secondary care in England, the main savings of NMP practices included 32% of GP appointments and 31% of follow-up appointments by a consultant.<br>- The annual value contributed by adding one new non-medical prescriber was over £270 million. The total cost-savings were predicted to be approximately £800 million in England in 2014.<br>- No NMP training-related costs (training course, travel, supervision time, etc) were reported.
Courtenay et al. (2015)[37]	Nurse IP	Patient-reported diabetes self-care activities; HbA1c test results; patients' satisfaction with activities, process and medicine management	Employment of cost; consultation cost; advice-seeking from other professionals and GP prescribing signing; prescribing costs; use of other health services	UK NHS	***Consequences***<br>- Patient satisfaction: in the nurse prescriber groups, patients were more satisfied with some specific aspects of care. However, there were no significant differences in general and overall satisfaction.<br>- Diabetes self-care activities: no significant differences were found.<br>- HbA1c test result: The HbA1c score decreased significantly in both groups over six months.<br>- Frequency of consultations: no significant differences were found.<br>***Costs***<br>- Consultation duration: longer consultations (average, 7.7 minutes) were provided by nurse prescribers for an additional cost of £6.<br>- New prescriptions and the use of healthcare services: no significant differences were reported regarding by patients in both groups.<br>- Most prescribing nurses were on a higher salary band than non-prescribing colleagues.
Neilson et al. (2015)[42]	Pharmacist IP	QALYs; chronic pain grade; anxiety and depression scale	Pharmacist training, pharmacist and GP time to deliver the intervention and provide follow-up; the number of hospitalisation days and outpatient visits); primary care visits for chronic pain (GP, nurse, healthcare assistant); telephone contacts for chronic pain; prescribed and non-prescribed OTC pain-related medications and health service resource use	UK NHS	***Consequences***<br>- QALYs: very small in both prescribing and review groups (relative to usual care, the mean differences in QALYs were 0.0069 for prescribing and 0.0097 for review groups, respectively)<br>***Costs***<br>- The average cost differences per patient were £77 for prescribing and £54 for review groups relative to usual care.<br>Overall, based on the expected value of sample information analysis the authors suggested a larger sample size (e.g. the optimal sample size was estimated at 780 for prescribing and 540 for the review group using a cost per QALY threshold of £20,000) for reliable findings.

For peer review only - http://bmjopen.bmj.com/site/about/guidelines.xhtml

Authors (year)	Type of prescribing	Outcome measures	Cost and resource use measures	Perspective	Study main findings
Norman et al. (2010)[43]	Nurse SP	Medication adherence; health status; adverse effects; patient satisfaction (with information, consultation, treatment, etc), patient's perception of improvement in their health problem; social functioning and impairment; depression scale	Training costs (time off work and other expenses, e.g. travel, supervision time, etc); costs of prescribing (e.g. time spent preparing for a prescribing, time taken to prescribe for a patient and number of patients prescribed for); patients' use of healthcare; cost of service per patient	Health and social care	***Consequences***<br>- Medicine adherence, health status, side effects, and satisfaction with overall care: no significant differences were reported between patients across the nurse and medical prescriber groups.<br>***Costs***<br>- Psychiatric inpatient costs were an average of £1,186 significantly higher per patient for the nurse prescriber group than those in the medical prescriber group.<br>- Total annual costs per patient: no significant differences were reported.<br>- The prescribing training course fee: £497 per patient.<br>- No additional training-related costs (travel, supervision time, etc) were reported.

Note. NMP: non-medical prescribing; PGD: patient group direction; IP: independent prescribing; SP: supplementary prescribing; CVD: cardiovascular disease; HRQoL: health-related quality of life; NHS: National Health Service; HbA1c: this refers to average blood sugar test. OTC: over the counter; QALYs: quality-adjusted life years. QALY is a generic metric used to value and quantify health outcomes in terms of both the quality and the quantity of life lived.[26]; ESRD: end-stage renal disease; VTE: venous thromboembolism, which manifests as either deep vein thrombosis (DVT) or pulmonary embolism (PE); GP: general practitioners; NA: not available.

For peer review only

For peer review only - http://bmjopen.bmj.com/site/about/guidelines.xhtml

Costs, consequences and value for money in non-medical prescribing: A scoping review

Journal:	*BMJ Open*
Manuscript ID	bmjopen-2022-067907.R2
Article Type:	Original research
Date Submitted by the Author:	20-Jan-2023
Complete List of Authors:	Babashahi, Saeideh; University of Sussex, Global Health and Infection<br>Carey, Nicola; University of the Highlands and Islands, Department of Nursing and Midwifery<br>Jani, Yogini; Imperial College London, Pharmacy Department<br>Hart, Kath; University of Surrey, Department of Nutritional Sciences<br>Hounsome, Natalia; Brighton and Sussex Medical School, Centre for Global Health Research
**Primary Subject Heading**:	Health economics
Secondary Subject Heading:	Global health, Health policy, Health services research
Keywords:	Health economics < HEALTH SERVICES ADMINISTRATION & MANAGEMENT, Health policy < HEALTH SERVICES ADMINISTRATION & MANAGEMENT, PUBLIC HEALTH

SCHOLARONE™
Manuscripts

I, the Submitting Author has the right to grant and does grant on behalf of all authors of the Work (as defined in the below author licence), an exclusive licence and/or a non-exclusive licence for contributions from authors who are: i) UK Crown employees; ii) where BMJ has agreed a CC-BY licence shall apply, and/or iii) in accordance with the terms applicable for US Federal Government officers or employees acting as part of their official duties; on a worldwide, perpetual, irrevocable, royalty-free basis to BMJ Publishing Group Ltd ("BMJ") its licensees and where the relevant Journal is co-owned by BMJ to the co-owners of the Journal, to publish the Work in this journal and any other BMJ products and to exploit all rights, as set out in our licence.

The Submitting Author accepts and understands that any supply made under these terms is made by BMJ to the Submitting Author unless you are acting as an employee on behalf of your employer or a postgraduate student of an affiliated institution which is paying any applicable article publishing charge ("APC") for Open Access articles. Where the Submitting Author wishes to make the Work available on an Open Access basis (and intends to pay the relevant APC), the terms of reuse of such Open Access shall be governed by a Creative Commons licence – details of these licences and which Creative Commons licence will apply to this Work are set out in our licence referred to above.

Other than as permitted in any relevant BMJ Author's Self Archiving Policies, I confirm this Work has not been accepted for publication elsewhere, is not being considered for publication elsewhere and does not duplicate material already published. I confirm all authors consent to publication of this Work and authorise the granting of this licence.

For peer review only

Costs, consequences and value for money in non-medical prescribing: A scoping review

Saeideh Babashahi[1,*], Nicola Carey[2], Yogini Jani[3], Kathryn Hart[4], Natalia Hounsome[5]

[1] Global Health and Infection, Brighton and Sussex Medical School, University of Sussex, Brighton, UK. Email: s.babashahi@bsms.ac.uk

[2] Department of Nursing and Midwifery, University of the Highlands and Islands, UK. Email: nicola.carey@uhi.ac.uk

[3] Centre for Medicines Optimisation Research and Education, UCLH NHS Foundation Trust and UCL School of Pharmacy, UK. Email: y.jani@ucl.ac.uk

[4] School of Biosciences & Medicine, Faculty of Health and Medical Sciences, University of Surrey Guildford, UK. Email: k.hart@surrey.ac.uk

[5] Global Health and Infection, Brighton and Sussex Medical School, University of Sussex, Brighton, UK. Email: n.ivashikina@bsms.ac.uk

* Correspondence: Saeideh Babashahi, Global Health and Infection, Brighton and Sussex Medical School, University of Sussex, Brighton, UK. Email: s.babashahi@bsms.ac.uk

Word count: 3898

For peer review only

ABSTRACT

Objectives: Non-medical prescribing (NMP) is a key feature of the UK healthcare system that refers to the legal prescribing rights granted to nurses, pharmacists and other non-medical healthcare professionals who have completed an approved training programme. NMP is deemed to facilitate better patient care and timely access to medicine. The aim of this scoping review is to identify, synthesise and report the evidence on the costs, consequences and value for money of NMP provided by non-medical healthcare professionals.

Design: Scoping review

Data sources: MEDLINE, Cochrane Library, Scopus, PubMed, ISI Web of Science and Google Scholar were systematically searched from 1999 to 2021.

Eligibility criteria: Peer-reviewed and grey literature written in English were included. The research was limited to original studies evaluating economic values only or both consequences and costs of NMP.

Data extraction and synthesis: The identified studies were screened independently by two reviewers for final inclusion. The results were reported in tabular form and descriptively.

Results: A total of 420 records were identified. Of these, nine studies evaluating and comparing NMP with patient group discussions, GP-led usual care, or services provided by non-prescribing colleagues were included. All studies evaluated the costs and economic values of prescribing services by non-medical prescribers, and eight assessed patient, health or clinical outcomes. Three studies showed pharmacist prescribing was superior in all outcomes and cost-saving at large scale. Others reported similar results in most health and patient outcomes across other non-medical prescribers and control groups. NMP was deemed resource-intensive to both providers and other groups of non-medical prescribers (e.g. nurses, physiotherapists, podiatrists).

Conclusions: The review demonstrated the need for quality evidence from more rigorous methodological studies examining all relevant costs and consequences to show value for money in NMP and inform the commissioning of NMP for different groups of healthcare professionals.

[Word count: 297]

Keywords: Non-medical prescribing (NMP), non-medical prescribers, health and non-health outcomes, economic impacts, cost-effectiveness, consequences, value for money

For peer review only - http://bmjopen.bmj.com/site/about/guidelines.xhtml

Strengths and limitations of this study

1) This scoping review addresses an under-researched area to provide evidence on resource use and consequences (e.g. service improvement, patient satisfaction, waiting times, safety, etc) of non-medical prescribing (NMP) from a large body of peer-reviewed and grey literature.

2) The review was limited to original studies that evaluated the economic impacts only or both costs and consequences of NMP.

3) Drawing conclusions on the cost-effectiveness and value for money in NMP remains difficult as the existing literature is heterogenous with significant variation in participants, NMP types, comparators, study designs, costs and consequences evaluated.

For peer review only

For peer review only - http://bmjopen.bmj.com/site/about/guidelines.xhtml

INTRODUCTION

Doctors have traditionally been authorised as the main group of healthcare professionals to prescribe medicines.[1],[2] With an increasing pace of population ageing and higher risks of chronic diseases, there is a growing demand for healthcare services and access to medicines.[3-5] Due, in part, to shortages within the medical workforce,[1],[6] the authority for other healthcare professionals, such as nurses and pharmacists, to prescribe medicines has been introduced in several countries such as the United States, United Kingdom (UK), Canada and Australia.[7-9]

Non-medical prescribing (NMP) is a key feature of the UK healthcare system that refers to the legal prescribing rights granted to nurses, pharmacists and other healthcare non-medical professionals who have completed an approved programme of education,[10-14] delivered via a variety of methods (often hybrid), including classroom teaching, one-to-one instruction, self-directed learning and e-learning.[15] NMP first emerged in the UK in 1999 for district nurses and health visitors,[2] and it came into effect for all registered nurses in 2001 and for pharmacists in 2003.[9],[16],[17] The UK has pioneered the gradual expansion of these prescribing roles to include a wider population of healthcare professionals across both primary care and secondary care.[8],[18] Since 2005 physiotherapists, podiatrists, and both diagnostic and therapeutic radiographers have been able to train to become supplementary prescribers (see Table 1 for definitions of supplementary prescribing (SP) and other terms).[11],[19] Independent prescribing (IP) rights were subsequently granted to optometrists in 2008[14],[20] and physiotherapists, podiatrists and chiropodists in 2013.[19],[21],[22] More recent changes in 2016 enabled therapeutic radiographers to train as independent prescribers,[23] and dietitians as supplementary prescribers[23] and in 2019 paramedics were awarded both IP and SP rights (see Table 1 for a glossary of terms).

For peer review only - http://bmjopen.bmj.com/site/about/guidelines.xhtml

Table 1. Definition of main terms (and variables) used in the study

Terminology	Definition
NMP	NMP is a term widely used in the UK, and it represents the prescribing authorities given to certain non-medical healthcare professionals (e.g. nurses, dietitians, physiotherapists) after completing a prescribing training course.[9],[15],[24]
IP	Those using IP are responsible for assessing patients' health conditions and making decisions about patients' treatment and clinical management, including prescribing, within their scope of practice.[9],[15],[24]
SP	Using SP, the initial assessment and diagnosis of a patient's condition are carried out by an independent prescriber (i.e. a GP or dentist), and the clinical condition is managed using a patient-specific clinical management plan agreed by the independent prescriber, supplementary prescriber and patient.[9],[15],[24]
PGD	PGD is a legal written framework that allows registered healthcare professionals to supply and/or prescribe specified medicines to a pre-defined group of patients without them having to see a medical prescriber (e.g. a GP).[7],[25]
Medicine management or prescribing activities	A system of processes that determines how medicines are used by patients and health providers. For the purposes of this study, medicine management and prescribing activities refer to prescribing and/or the process of giving advice about medicines and the supply of medicines, as described in the research questions sub-section.[18],[21]
Cost and resource use	This refers to the direct and/or indirect medical and/or non-medical resources consumed by the study population and/or the costs associated with setting up and implementing the intervention(s) under study.[26]
Consequence	This refers to the health, non-health, clinical and patient outcomes representing the effects of the intervention(s) under study.[26]
Perspective	This refers to (one or more groups of) stakeholders' viewpoints from which economic evaluation or cost analysis is conducted.[26] Examples include the patient perspective, societal perspective or healthcare provider perspective.
Comparator	This refers to the alternative courses of action (e.g. usual care) against which the intervention under study (e.g. NMP, the subject of this study) is evaluated.[26]

Note. NMP: non-medical prescribing; IP: independent prescribing; SP: supplementary prescribing; PGD: patient group direction; GP: general practitioner

Reviews of NMP developments and its benefits in the UK and other countries have been reported by others.[25],[27],[10] Although NMP is embedded within UK healthcare delivery in primary and secondary care, there is still a lack of evidence regarding its value for money.[16],[21],[28-31] Building on an earlier review by Noblet et al. (2018) on the effectiveness and cost-effectiveness of NMP from three randomised controlled trials (RCTs),[28] this scoping review aimed to assess a wider body of literature, including both peer-reviewed and grey

For peer review only - http://bmjopen.bmj.com/site/about/guidelines.xhtml

literature, to identify evidence on costs and consequences and the value for money of non-medical prescribing.

METHODS

The scoping review protocol was registered with the Open Science Framework Registry on 31 July 2021 (registered DOI: 10.17605/OSF.IO/PSR3N, accessible from https://osf.io/psr3n). We followed the Preferred Reporting Items for Systematic Reviews and Meta-Analysis extension for Scoping Reviews (PRISMA-ScR) reporting guideline recommended by Tricco et al. (2018) to report our scoping review study.[32] This scoping review was conducted using the five-stage methodological framework developed by Arksey and O'Malley and further developed by Levac et al. and the Joanna Briggs Institute to ensure rigour in reporting the review and its methodology.[33-35] The five stages are outlined below:

Stage 1: identifying the research questions

1) What types of prescribing practices (e.g. SP, IP) have been implemented and evaluated across eligible groups of healthcare professions (e.g. pharmacists, podiatrists, dietitians, etc) in different studies?

2) What measures and tools have been used to evaluate the economic values, safety, effectiveness and other consequences of prescribing by non-medical prescribers in various settings?

3) What are relevant costs, resource use, and consequences (e.g. health, non-health and clinical outcomes) associated with services provided by non-medical prescribers in both peer-reviewed and grey literature?

For peer review only - http://bmjopen.bmj.com/site/about/guidelines.xhtml

Stage 2: search strategy and screening

The scope and practice of NMPs vary globally.[7],[18],[30],[36],[37] For the purposes of this review, NMP was assumed to include medicine management activities that are legally and technically considered prescribing and provided by healthcare professionals who are eligible to prescribe and have completed an approved programme of education. Consistent with Courtenay et al. (2012) and Carey et al. (2020), these medicine management activities include "making recommendations for patients to buy medicine(s) over the counter; amending prescribed medication; medication review; written recommendation to GP; recommending in patients' hospital notes; prescribing via hospital medication charts; patient group directions; remote prescribing via telephone, email and fax; issuing hospital-specific prescription; signing issued prescription via GP repeat prescribing system; issuing private prescription directly to the patient".[21],[30]

A comprehensive search strategy was developed by the research team to enable a stepwise search process. Based on initial exploratory research, we included grey literature and journal and conference articles with full-text written in English from 1999 to 2021.[24] On 14 January 2022, we searched PubMed, Cochrane Database of Systematic Reviews, Web of Science, Scopus and MEDLINE databases for articles published between 1 January 1999 and 1 January 2022.

The detailed search terms and strategies for different databases are presented in Tables A.1 to A.5 in Supplementary Data. A non-systematic search in Google Scholar was performed to find the grey literature. The search terms used for Google Scholar are equivalent to those of other search engines. In brief, our search strategy included (non-medical prescrib* OR NMP OR non-doctor prescrib*) AND (pharmac* OR nurs* OR non-medical healthcare professionals OR allied health professionals OR AHPs OR diet* OR radiograph* OR midwiv* OR physiotherap* OR podiatr* OR optometr* OR paramedic*) AND (consequences OR health

For peer review only - http://bmjopen.bmj.com/site/about/guidelines.xhtml

outcomes OR non-health OR clinical outcomes OR effectiveness OR patient outcomes) AND (economic impacts OR costs OR resource use).

The scoping review included original research, RCT studies and grey literature analyses of resource use only or both consequences and costs to evaluate NMP provided by non-medical healthcare professionals. Commentaries, letters, protocols and editorials were excluded. A broad search strategy was implemented to ensure that the inclusion of studies was as comprehensive as possible. Search terms were derived from titles, abstracts, and keywords identified in key publications and from search terms used in previous reviews related to non-medical prescribing.[8],[9],[29] In addition, relevant references of included studies were checked (snowballing search).

All articles identified from the searches were transferred to the EndNote reference manager software version 20.2, and all duplicates and titles in languages other than English were removed. The PICOS (population, intervention, context, outcome, and study design) framework was used to establish eligibility criteria.[38] Table 2 provides further information regarding the inclusion criteria according to the PICOS approach.

Table 2. PICOS table describing inclusion criteria

Component	Description
Population	- Human participants (e.g. nurse, pharmacist and other non-medical prescribers, and patients with any health conditions managed by these groups)<br>- No restriction on age or gender
Intervention	- All types of non-medical prescribing (any medicine management activity that is legally and technically considered prescribing and provided by non-medical professionals)
Context	- All peer-reviewed published articles (in journals and conferences) and grey literature with full-text written in English from 1999 to 2021<br>- No restrictions on setting or country
Outcome	- Cost and consequence outcomes of NMP services provided by nurses, pharmacists and other non-medical prescribers
Study design	- Original research and clinical trials that evaluated costs and economic impacts of NMP only or both cost and consequence outcomes of NMP (peer-reviewed or grey literature)

Note. PICOS: (population, intervention, context, outcome, and study design); NMP: non-medical prescribing.

For peer review only - http://bmjopen.bmj.com/site/about/guidelines.xhtml

Stage 3: study selection

The review process included an initial screening of the title and abstract of the studies by three authors (SB, NH and NC) to assess their eligibility for full-text retrieval. Any studies that were not excluded confidently through title and abstract screening during the initial screening step were included for full-text screening. The full-text screening of the selected studies was divided between authors and carried out independently by two reviewers (SB, NH, YJ and KH). Any disagreement on selected papers was resolved through discussion among the authors. After identifying and removing duplicates, studies were excluded if (1) they were not original studies, (2) no abstract or full-text was available, (3) they were not in English, or (4) the focus of the study was outside the scope of our review (see Table 2), or (5) prescribing and medicine management activities evaluated did not meet those indicated by Courtenay et al. (2012) and Carey et al. (2020).[21],[30]

Stage 4: data extraction and analysis

Data from the articles and grey literature based on the inclusion criteria mentioned above were extracted using a bespoke data extraction form. A Microsoft Excel 2019 based form was initially developed by the first author and validated by other authors for charting the data from selected studies and reporting the variables regarding the study, participants, interventions and outcome characteristics – e.g. authors, publication year, study context and design, sample size, type of prescribing, cost and consequence outcomes measures and key findings – based on our research questions (Table 1 represents the definition of the main variables). Data extracted were checked by a second reviewer for accuracy and completeness.

Stage 5: collating and reporting the results

The PRISMA-ScR reporting checklist (Table B, Supplementary Data) was used to synthesise and report the results of our scoping review.[32] Data synthesis was undertaken by the first author in consultation with the research team. The findings of selected studies were

For peer review only - http://bmjopen.bmj.com/site/about/guidelines.xhtml

summarised and presented in tabular forms and descriptively highlighting the key research findings (e.g. economic impacts, consequences of NMP, setting, NMP type, etc) of selected studies and the existing research gaps around NMP practice.

Patient and Public Involvement

No patients or public were involved in the study.

RESULTS

Database search findings

The database search generated 420 records. A total of 236 records were removed due to duplication. Of the remaining 184 records, we excluded 171 records in the initial review of titles and abstracts as these studies were not original research evaluating NMP. For the remaining 13 records, the full-text papers were independently reviewed by two reviewers, and a further four studies were excluded because they did not report resource use and economic impacts of NMP or did not fit within our definition of prescribing and medicine management activities. Nine studies were included in the final review (eight original research studies[7],[21],[37],[39-43] and one grey literature paper[44]). Figure 1 shows the PRISMA flow chart of the included studies in our scoping review.

General characteristics of included studies

The key characteristics of included papers are summarised in Table 3 and Table C (Supplementary Data). Of the nine papers, six were from the UK,[7],[21],[37],[42-44] two from Canada,[39],[41] and one from Australia.[40] Papers were published between 2010 and 2022 and evaluated the impact of NMP practices by pharmacists (n=4),[39],[40-42] nurses (n=3),[7],[37],[43] physiotherapists and podiatrists (n=1),[21] and another estimating NMP cost-savings in primary and secondary care for a range of health professions.[44] Types of prescribing services

For peer review only - http://bmjopen.bmj.com/site/about/guidelines.xhtml

evaluated in these studies included SP (n=2)[44],[43] or IP (n=8)[7],[21],[37],[39-42],[44] and community nursing.[44]

For peer review only

For peer review only - http://bmjopen.bmj.com/site/about/guidelines.xhtml

Table 3: Overview and general characteristics of included studies

Authors (year)	Context (country/setting)	Study design	Type of prescribing	Comparator	Medicine management or prescribing activity	Study population
Black et al. (2022)[7]	UK, urban sexual health services	Mixed methods and a comparative case study (cost-consequence framework)	Nurse IP	PGD by non-prescribing nurses	Prescribed medications	N = 26 nurse prescribers<br>N = 67 PGDs users
Carey et al. (2020)[21]	UK mixed range of settings (primary and secondary care, social enterprise and private practice)	Mixed methods and a comparative case study (cost-consequence framework)	Physiotherapist IP<br>Podiatrist IP	Non-prescribing physiotherapists<br>Non-prescribing podiatrists	Prescribed and reviewed medications	N = 488 patients (243 IP sites and 245 NP sites)<br>N = 7 matched pairs of IP and NP sites (3 podiatrists and 4 physiotherapists)
Al Hamarneh et al. (2019)[39]	Canada, primary care (cardiovascular risk reduction)	Cost-effectiveness analysis (Markov model)	Pharmacist IP	Usual care	Prescribed and reviewed medications	The authors developed their model based on the population observed in the R_XEACH trial[45] as follows:<br>N = 723 patients (370 in intervention and 353 in control)<br>N = 54 pharmacies in the RCT
Hale et al. (2018)[40]	Australia, an elective surgery pre-admission clinic (venous thromboembolism)	Cost-effectiveness analysis (decision tree model)	Pharmacist IP	Usual care	Prescribed medications	The authors developed their model based on the population observed in an earlier trial[46] as follows:<br>N = 384 patients (194 in intervention and 190 in control)<br>N = 1 pharmacist prescriber<br>N = 59 medical prescribers
Marra et al. (2017)[41]	Canada, community care, hospitals, or primary care (hypertension)	Cost-effectiveness analysis (Markov model)	Pharmacist IP	Usual care	Prescribed medications	The authors developed their model based on the population observed in the R_XACRION trial[47] as follows:<br>N = 248 patients (181 in intervention and 67 in control)<br>N = 20 pharmacists practised in the community<br>N = 2 pharmacists from hospital outpatient clinics<br>N = 6 pharmacists from primary care clinics

For peer review only - http://bmjopen.bmj.com/site/about/guidelines.xhtml

i5 Health (2015)[44]	England, various settings (e.g. primary and secondary care)	Economic analysis of audits, self-reported questionnaires, interviews	IP and SP (for a range of professions, e.g. physiotherapists, podiatrists, midwives and radiographers)<br>Community nurse prescribers	NA	NA	Based on an estimation of the NMP practitioners registered with Northwest England NHS trusts (N = 1,566 unique prescribers)
Courtenay et al. (2015)[37]	England, primary care (type 2 diabetes)	Mixed methods and a comparative case study (cost-consequence framework)	Nurse IP	Non-prescribing nurses	Prescribed and reviewed medications, recommended decisions, provided advice, and discussed medications with GPs or colleagues	N = 12 general practices (6 prescribing nurses and 6 non-prescribing nurses)<br>N = 214 patients (131 in nurse prescriber sites and 83 in non-prescriber sites)
Neilson et al. (2015)[42]	UK, primary care (chronic pain)	Regression analysis of costs and effects; the expected value of sample information analysis	Pharmacist IP	Usual care	Prescribed and reviewed medications	N = 6 general practices<br>N = 125 patients (39 in prescribing, 44 in review and 42 in usual care arms)<br>No information is provided about the number of non-medical prescribers in the two groups
Norman et al. (2010)[43]	UK, primary care (mental health)	Cost-consequences analysis; matched post-test control study	Nurse SP	Usual care	Prescribed medicines	N = 90 patients (45 matched pairs)<br>No information is provided about the number of prescribers in the two groups

Note. PGD: patient group direction; IP: independent prescribing; NP: non-prescribing; SP: supplementary prescribing; RCT: randomised controlled trial; GP: general practitioner; NA: not available; NMP: non-medical prescribing.

For peer review only - http://bmjopen.bmj.com/site/about/guidelines.xhtml

Methodological and reporting considerations

Three out of nine papers conducted a model-based economic evaluation (i.e. cost-effectiveness analysis) using the outcomes from an earlier trial with an assessment of uncertainty (in the form of a deterministic and/or probabilistic sensitivity analysis).[39],[40],[41] Four studies conducted a cost-consequence-based approach listing costs and outcomes of NMP without assessing sources of uncertainty.[7],[21],[37],[43] A bottom-up costing approach (using detailed input data from records or questionnaires at the service provider level) was used in most studies with clear information on costs per unit.[7],[21],[37],[39-42] Overall non-model-based studies did not provide an explanation of sample size sufficiency. Only one study suggested that determining an optimal and larger sample size would be required to draw a precise and accurate conclusion.[42] Two studies failed to specify the number or characteristics of the study participants (e.g. non-medical prescribers or patients).[42],[43]

Measures of costs of NMP

The resource use and costs evaluated in the included studies fall into the following three main categories:

i) Prescribing training course

Four out of nine articles applied direct costs associated with prescribing training and NMP courses (e.g. training course fee, supervision time, employer-paid study time),[7],[39],[41],[42] with one study using time-off-work to complete the course.[43] Other relevant expenses such as out-of-pocket expenses (e.g. travel, accommodation, etc) by qualified non-medical prescribers and their (unpaid) personal study time were included in two studies.[7],[43]

ii) Prescription (and consultation)

For peer review only

Expenses applied in this category by some studies included tests and other relevant services, referrals to other healthcare professionals, frequency of follow-up, time spent preparing for a prescribing consultation, time taken to prescribe, review or complete the medication plan for a patient, and the number of patients prescribed for, consultation frequency, time spent discussing the patient and obtaining prescriptions or clinical advice sought from GPs or other NMP practitioners, unplanned consultations for the health condition after the index consultation and frequency of new medications.[7],[21],[37],[39],[40],[42],[43] Incorrect or over-prescribing was identified and considered as an indication of wastage, and the 'wasted' medication, as well as under-prescribed medicine that should have been prescribed, were considered as another source of cost.[7],[43]

iii) Other relevant expenses

Some studies also considered the expenses associated with service utilisation, e.g. hospital admissions, outpatient expenses, inpatient days and A&E visits,[21],[37],[41-43] or the healthcare and medical costs associated with targeted health conditions across case and control groups.[39],[40],[41]

Measures of consequences of NMP

Health-related quality of life was one of the main health outcomes evaluated using EQ-5D or SF-6D questionnaires,[21],[39],[40-42] and the benefits to patients of appropriate prescribing were measured in terms of increased quality-adjusted life years (QALYs) in some of these studies.[39],[40-42] Multiple studies evaluated patient experience and satisfaction as one of the main patient outcomes.[7],[21],[37],[43] Life years gained were applied by two studies.[39],[41] Medicine adherence and ease of access to services were other outcomes reported in one study.[21] Examples of specific clinical and health outcomes used in the studies were self-care and relevant clinical indicators such as HbA1c test results (mean blood sugar level) and body

For peer review only - http://bmjopen.bmj.com/site/about/guidelines.xhtml

mass index for patients with diabetes,[37] the reduced risk of the disease under study (e.g. venous thromboembolism, cardiovascular disease)[39],[40],[41] and reduced blood pressure.[41]

Key findings: the costs and consequences of NMP

A summary of the key cost and consequence findings is provided for pharmacists, nurses and other non-medical prescribers in turn (see Table C in Supplementary Data for detailed information).

Pharmacists

The NMP practices by pharmacists were evaluated across a range of health conditions (e.g. venous thromboembolism, hypertension, etc), and significant improvements in health and clinical outcomes were reported at the end of the observation in three studies.[39],[40],[41] As such, Marra et al. (2017) found the 30-year risk of cardiovascular disease (CVD) in the pharmacist prescriber group was reduced from 0.61 in base-case to 0.41 (indicating a reduction of two CVD events in every 10 individuals receiving the intervention).[41] Although the intervention was associated with increased costs of CA$7,145 due to the intervention itself and medications, this was compensated for by a reduction of CA$15,094 in CVD and other co-morbidities costs, suggesting pharmacist independent prescribing was less costly and more effective than usual care.[41]

Consistent with Marra et al. (2017), two other studies – i.e. Al Hamarneh et al. (2019) and Hale et al. (2018) – reported that pharmacist prescribing was cost-effective and cost-saving for patients with CVD and venous thromboembolism, respectively.[39],[40] Only Neilson et al. (2015) found that, relative to the usual-care arm, pharmacist prescribing for chronic pain was more costly (£77.5 for prescribing and £54.4 for review arms) and provided similar QALYs. Neilson et al. recruited a total sample of 125 patients in this RCT, but the authors recommended a larger sample size (between 460–690 for a threshold of £30,000 QALY gained or 540–780 for a threshold of £20,000 QALY gained) according to an expected value

For peer review only - http://bmjopen.bmj.com/site/about/guidelines.xhtml

of sample information analysis (indicating that additional information collected from a larger sample will reduce uncertainty and provide more reliable data).[42]

Nurses

Norman et al. (2010) indicated that patients in the mental health nurse prescriber group had a significantly higher level of satisfaction with nurse prescribers than those in the medical prescriber group.[43] Similarly, Courtenay et al. (2015) reported that the average patient satisfaction for some specified aspects of care was significantly higher among diabetic patients in the nurse prescriber group than among those of the non-prescribing nurses.[37] Nonetheless, no significant differences were reported with respect to patients' overall satisfaction by Courtenay et al. (2015) and Black et al. (2022). Other specific or generic health and social outcomes were found to be similar among nurse prescribers and the control groups in these three studies.[7],[37],[43]

For peer review only

NMP was deemed resource-intensive to both providers and nurse prescribers. According to Black et al. (2022), the training-related costs included the course fee (paid fully by employers or training grants – ranging from £900 to £3,555 in 2016), an average of 20.1 of employer-paid study days for 92% of nurse prescribers (ranging from 1 to 31 funded by employer), and an average of 7.4 supervised days (ranging from 2 to 13.7 day, incurring a cost of £6,451 to the NHS for each nurse – ranging from £1,283 to £11,138) during training.[7] It is important to note that although PGDs (i.e. patient group directions, please see Table 1 for more information) provide a legal framework for health professionals to supply and administer a specified medicine to a pre-defined group of patients, and there is no mandatory training required prior to their use; there are limitations to their use, indicating that NMP might be worth the training cost.[7],[25] Employment costs of prescribing nurses were deemed potentially higher as they were on higher pay bands compared to non-prescribing nurses, including PGD users.[7],[37],[43] Consultation durations and unplanned re-consultations were similar for both sexual health nurse prescribers and medical prescribers, as reported by Black et al. (2022).[7]

However, Courtenay et al. (2015) reported longer consultations for patients with diabetes managed by nurse prescribers suggesting it was more costly relative to GPs and non-prescribing nurses.[37] No statistically significant differences in prescribing new medicines or use of other healthcare services between groups were identified in any of the three studies assessing this outcome.[7],[37],[43]

Other non-medical prescribers

Only one study evaluated the benefits and costs of services by physiotherapist and podiatrist independent prescribers compared to non-prescribing physiotherapists and podiatrists.[21] Carey et al. (2020) showed the level of satisfaction with consultation and services was significantly higher in both non-medical prescriber groups. Patients of physiotherapist or podiatrist independent prescribers were more likely to receive medicine information or advice during consultations (39.7%) compared to patients managed by non-prescribers (24.5%). No significant differences were reported in the quality of life in patients for all groups.[21] Consultation durations were longer for both prescriber groups, resulting in increased costs for prescribing physiotherapists (£7.95 per contact) and prescribing podiatrists (£8.62) compared to non-prescribers. No training-related costs were reported.[21]

DISCUSSION

Building on a previous systematic review[28] which included only three RCTs published before 2015, we have included a wider range of studies evaluating the consequences, resource use, costs and value for money in NMP. We used the PRISMA-ScR framework to guide the review, searched multiple databases and used snowballing techniques to improve the comprehensiveness of the study. Despite this, only one additional source of evidence from the grey literature was identified. The NMP literature has largely focused on assessing the benefits and effectiveness of prescribing authorities without evaluating the costs and resource use. Some other studies have concentrated on topics such as NMP trends and related national

For peer review only - http://bmjopen.bmj.com/site/about/guidelines.xhtml

policies over time or implementation barriers and/or facilitators of NMP for different professions.[1],[9],[18],[24],[27],[48] This review demonstrated the lack of evidence on costs, consequences and value for money in NMP by different groups of healthcare professionals.

Our scoping review identified nine sources of evidence that evaluated the economic impacts, resource use and consequences of NMP. Three studies showed pharmacist prescribing was superior in all outcomes and cost-saving at large scale. Others reported similar results in most health, clinical and patient outcomes across other non-medical prescribers and control groups (e.g. GP-led usual care). NMP was deemed resource-intensive to both providers and other groups of non-medical prescribers (e.g. nurses, physiotherapists, podiatrists).

This scoping review revealed evidence sources were heterogeneous with regard to design, setting, range of cost and consequence outcomes, NMP types and comparators. In general, the existing evidence indicates that services provided by non-medical prescribers might positively influence patients' satisfaction with care, medication and their quality of life.[21],[37],[39],[40],[41] However, some of these findings came from non-RCT studies without robust evaluation of all relevant consequences and costs. Besides, some of these studies recruited small sample sizes, suggesting it is difficult to make any statement about the significance of the results beyond the sample included and therefore, these findings should be treated with caution.

The costs and consequences evidence on NMP has slowly grown since 2010 and appears to be concentrated in three countries, the UK, Canada and Australia, where prescribing rights are more developed. Despite the large increase in NMP in the UK and around the world and the increasing number of studies on NMP, there is still very limited information on the effectiveness, costs and cost-effectiveness of NMP by different professions. Many papers evaluated NMP delivered by nurses and pharmacists using various sources of costs, health, clinical and patient outcomes with varied comparators for a range of health conditions, which limits their generalisability and usefulness for other settings and professions. Only three

For peer review only - http://bmjopen.bmj.com/site/about/guidelines.xhtml

studies conducted a cost-effectiveness analysis to evaluate and demonstrate value for money in pharmacist prescribing.[9],[39],[41] Other studies used a cost-consequence approach (CCA) that provided disaggregated costs and outcomes of NMP for nurses, physiotherapists and podiatrists.[7],[21],[37] Although CCA helps identify and list relevant costs and outcomes associated with the interventions, it does not provide a definitive cost-outcome ratio and definite cost-effectiveness results for the interventions under study.[26]

The number of studies, particularly economic evaluation studies, assessing the economic burden and effectiveness of NMP has been increasing, but there is still a dearth of evidence on the cost-effectiveness and value for money in NMP authorities by recently awarded non-medical prescribers such as radiographers and dietitians. As most cost-effectiveness evidence relates to pharmacists, it is important to evaluate the impact, safety, resource use and economic value of prescribing by non-medical prescribers in other professions to inform policy and practice around NMP where it provides value for money. It is also important to acknowledge and further explore the challenges related to capturing these data, as NMP has been introduced as an additional role for healthcare professions, and hence it is not easy to separate and capture some of the added costs and values in terms of these additional prescriptive authorities.[21],[28]

There seem to be research-quality gaps in the literature. Although we did not assess the quality of included studies, some of the studies have performed non-model-based analysis using small samples that might affect the analysis, and in some cases, the main outcomes and sources of costs (e.g. training-related, etc) were not included in the analysis. Despite the importance of rigour in quantitative research, sample size reporting and sufficiency assessment remained inconsistent and partial in these studies.

Strengths and limitations

A rigorous search was conducted, allowing for a diverse set of literature (from both peer-reviewed and grey) to be identified in a robust and reproducible manner. To our knowledge,

For peer review only - http://bmjopen.bmj.com/site/about/guidelines.xhtml

this is the first scoping review covering and representing the largest and most up-to-date evidence on the costs and consequences of prescribing practices by nurses, pharmacists and other non-medical prescribers. This scoping review contributes to the discussion of the costs and consequences of NMP and the existing research gaps regarding value for money in NMP for different groups of healthcare professionals. Original studies that did not report resource use and costs associated with NMP were not included in our review. While this strategy contributed to a more focused search, studies that reported only the effectiveness and benefits of NMP practices without evaluating costs are missing. Comparison of studies was challenged by heterogeneity regarding the profession, type of NMP, costs and consequences evaluated.

CONCLUSION

NMP practice is now an integrated feature of healthcare delivery in the UK and around the world, but considerable uncertainty remains regarding the costs, consequences and cost-effectiveness of the prescribing rights granted to non-medical prescribers, including therapeutic radiographers and dietitians. In order to determine accurate mean values and detect cost and benefit differences across non-medical prescribers and control groups, it is important that future studies involve larger and more representative samples with greater power. Adopting a model-based approach within each profession using targeted outcome measures would also enable a more robust comparison and improve understanding of how to best utilise NMP and healthcare professionals' skills and ensure it offers a cost-effective solution to providing faster and improved access to medicine and healthcare services for patients by the most appropriate healthcare professionals.

Figure 1. PRISMA flowchart of the study selection process

For peer review only - http://bmjopen.bmj.com/site/about/guidelines.xhtml

Acknowledgements: None.

Author Contributions: SB: Study design; formulation of search strategies; investigation, screening and review – titles, abstracts and full-texts; data extraction; writing – original draft, review & editing; NC, YJ, KH, NH: Study design; formulation of search strategies; screening and review – full-texts, data extraction; writing – review & editing. All authors read and approved the final version of the manuscript before submission.

Funding: This scoping review is conducted as part of the health economic evaluation in the TRaDiP project that was funded by the Department of Health Policy Research Programme, the National Institute for Health Research (grant number: PRP12697/86202356).

Conflict of interest: Authors declare no competing interest.

Data availability: All data are available within the article and the supplementary materials.

Ethics statements

Ethics approval: This scoping review study does not require ethical approval as it consists of reviewing and collecting secondary data from already published papers.

Patient consent for publication: Not required.

For peer review only

REFERENCES

1. Cooper R, Bissell P, Ward P, et al. Further challenges to medical dominance? The case of nurse and pharmacist supplementary prescribing. *Health & Social Care in the Community* 2012;**16**(2):115–133. https://doi.org/10.1177/1363459310364159.

2. Cooper R, Guillaume L, Avery T, et al. Nonmedical prescribing in the United Kingdom: developments and stakeholder interests. *Journal of Ambulatory Care Management* 2008;**31**(3):244–52. https://doi.org/10.1097/01.JAC.0000324670.91153.b4.

3. World Health Organization. Ageing and health. World Health Organization, 2021. https://www.who.int/news-room/fact-sheets/detail/ageing-and-health. (Accessed 12 March 2022).

4. Maresova P, Javanmardi E, Barakovic S, et al. Consequences of chronic diseases and other limitations associated with old age - a scoping review. *BMC Public Health* 2019;**19**(1),1431. https://doi.org/10.1186/s12889-019-7762-5.

5. Kingston A, Comas-Herrera A, Jagger C, et al. Forecasting the care needs of the older population in England over the next 20 years: estimates from the Population Ageing and Care Simulation (PACSim) modelling study. *Lancet Public Health* 2018;**3**(9):e447–e455. https://doi.org/10.1016/S2468-2667(18)30118-X.

6. Nancarrow SA, Borthwick AM. Dynamic professional boundaries in the healthcare workforce. *Socioogy of Health and Illness* 2005;**27**(7):897–919. https://doi.org/10.1111/j.1467-9566.2005.00463.x.

7. Black A, Courtenay M, Norton C, et al. Independent nurse medication provision: A mixed method study assessing impact on patients' experience, processes, and costs in sexual health clinics. *Journal of Advanced Nursing* 2022;**78**(1):239–251. https://doi.org/10.1111/jan.15075.

8. Kroezen M, van Dijk L, Groenewegen PP, et al. Nurse prescribing of medicines in Western European and Anglo-Saxon countries: a systematic review of the literature. *BMC Health Services Research* 2011;**11**,127. https://doi.org/10.1186/1472-6963-11-127.

9. Graham-Clarke E, Rushton A, Noblet T, et al. Non-medical prescribing in the United Kingdom National Health Service: A systematic policy review. *PLoS One* 2019;**14**(7):e0214630. https://doi.org/10.1371/journal.pone.0214630.

10. Department of Health. The National Health System plan: A plan for investment. A plan for reform. Department of Health, 2000. http://1nj5ms2lli5hdggbe3mm7ms5.wpengine.netdna-cdn.com/files/2010/03/pnsuk1.pdf. (Accessed 12 August 2021).

11. Health and Care Professions Council. Non-medical prescribing approved courses. Health and Care Professions Council, 2021. https://www.hcpc-uk.org/education/approved-programmes/. (Accessed 10 August 2021).

12. General Pharmaceutical Council. Discussion paper on supervising pharmacist independent prescribers in training. General Pharmaceutical Council, 2016. https://www.pharmacyregulation.org/sites/default/files/discussion_paper_on_supervising_pharmacist_independent_prescribers_in_training_november_2016.pdf. (Accessed 12 March 2021).

13. Nursing and Midwifery Council. Standards for prescribing programmes. Nursing and Midwifery Council, 2018. https://www.nmc.org.uk/standards/standards-for-post-registration/standards-for-prescribers/standards-for-prescribing-programmes/. (Accessed 14 March 2021).

14. General Optical Council. Independent Prescribing. General Optical Council, 2021. https://optical.org/en/news/news-and-press-releases/goc-council-approves-updated-requirements-for-independent-prescribing-categories/. (Accessed 10 March 2021).

15. Black A, Gage H, Norton C, et al. Patient satisfaction with medication consultations and medicines information provided by nurses working autonomously in sexual health services: A questionnaire study. *Journal of Advanced Nursing* 2022;**78**(2):523–531. https://doi.org/10.1111/jan.15087.

16. Cope LC, Abuzour AS, Tully MP. Nonmedical prescribing: where are we now? *Therapeutic Advances Drug Safety* 2016;**7**(4):165–72. https://doi.org/10.1177/2042098616646726.

For peer review only - http://bmjopen.bmj.com/site/about/guidelines.xhtml

17. Department of Health and Social Security. Medicinal Products; Prescriptions by Nurses etc Act. Department of Health and Social Security, 1992. https://www.legislation.gov.uk/ukpga/1992/28/contents. (Accessed 10 February 2022).

18. Courtenay M, Carey N, Stenner K. Non medical prescribing leads views on their role and the implementation of non medical prescribing from a multi-organisational perspective. *BMC Health Services Research* 2011;**11**:142. https://doi.org/10.1186/1472-6963-11-142.

19. Allied Health Professions Federation. Outline curriculum framework for education programmes to prepare physiotherapists and podiatrists as independent/supplementary prescribers and to prepare radiographers as supplementary prescribers. Allied Health Professions Federation, 2013. http://www.ahpf.org.uk/AHP_Prescribing_Programme_Information.htm. (Accessed 10 March 2022).

20. Rumney N. Optometry and independent prescribing. *Journal of Prescribing Practice* 2019;**1**(2):87–92. https://doi.org/10.12968/jprp.2019.1.2.87.

21. Carey N, Edwards J, Otter S, et al. A comparative case study of prescribing and non-prescribing physiotherapists and podiatrists. *BMC Health Services Research* 2020;**20**(1):1074. https://doi.org/10.1186/s12913-020-05918-8.

22. Morris L. Nonmedical independent prescribing: Podiatrists to take the next step. *The Diabetic Foot Journal* 2014;**16**:146–50.

23. National Health Service. Non-medical prescribing by allied health professionals. National Health Service, 2016. https://www.england.nhs.uk/ahp/med-project/. (Accessed 10 March 2022).

24. Babashahi S, Carey N, Hart K, et al. Costs and consequences of services provided by non-medical prescribers: a scoping review protocol. *Journal of Prescribing Practice* 2022;**4**(4):160–164. https://doi.org/10.12968/jprp.2022.4.4.160.

25. Department of Health. Health Services Circular (HSC 2000/026): Patient Group Directions. Department of Health, 2000. http://webar chive.natio nalarchives.gov.uk/20130 10710 5354/http:/www.dh.gov.uk/prod_consum_dh/group s/dh_digit alass ets/@dh/@en/docum ents/digit alass et/dh_40122 60.pdf. (Accessed 15 July 2021).

26. Drummond M, Sculpher M, Torrance G. Methods for the economic evaluation of health care programmes. Oxford University Press: Oxford 2005.

27. Wilson D, Murphy J, Nam M, et al. Nurse and midwifery prescribing in Ireland: A scope-of-practice development for worldwide consideration. *Nursing and Health Sciences* 2018;**20**:264–70. https://doi.org/10.1111/nhs.12408.

28. Noblet T, Marriott J, Graham-Clarke E, et al. Clinical and cost-effectiveness of non-medical prescribing: A systematic review of randomised controlled trials. *PLoS One* 2018;**13**(3):e0193286. https://doi.org/10.1371/journal.pone.0193286.

29. Alghamdi SSA, Hodson K, Deslandes P, et al. Prescribing trends over time by non-medical independent prescribers in primary care settings across Wales (2011-2018): a secondary database analysis. *BMJ Open* 2020;**10**(10):e036379. https://doi.org/10.1136/bmjopen-2019-036379.

30. Courtenay M, Carey N, Stenner K. An overiew of non medical prescribing across one strategic health authority: a questionnaire survey. *BMC Health Services Research* 2012;**12**:138. https://doi.org/10.1186/1472-6963-12-138.

31. Graham-Clarke E, Rushton A, Marriott J. A Delphi study to explore and gain consensus regarding the most important barriers and facilitators affecting physiotherapist and pharmacist non-medical prescribing. *PLoS One* 2021;**16**(2):e0246273. https://doi.org/10.1371/journal.pone.0246273.

32. Tricco AC, Lillie E, Zarin W, et al. PRISMA Extension for Scoping Reviews (PRISMA-ScR): Checklist and Explanation. *Annals of Internal Medicine* 2018;**169**(7):467–473. https://doi.org/10.7326/M18-0850.

33. Arksey H, O'Malley L. Scoping studies: towards a methodological framework. *International Journal of Social Research Methodology* 2005;**8**:19–32. https://doi.org/10.1080/1364557032000119616.

For peer review only - http://bmjopen.bmj.com/site/about/guidelines.xhtml

34. Levac D, Colquhoun H, O'Brien K. Scoping studies: advancing the methodology. *Implementation Science* 2010;**5**(1):1–9. https://doi.org/10.1186/1748-5908-5-69.

35. Joanna Briggs Institute. The Joanna Briggs Institute Reviewers' Manual 2015: Methodology for JBI Scoping Reviews, 2015. The Joanna Briggs Institute. www.joannabriggs.org. (Accessed 15 July 2021).

36. Gielen SC, Dekker J, Francke AL, et al. The effects of nurse prescribing: a systematic review. *International Journal of Nursing Studies* 2014;**51**(7):1048–1061. https://doi.org/10.1016/j.ijnurstu.2013.12.003.

37. Courtenay M, Carey N, Gage H, et al. A comparison of prescribing and non-prescribing nurses in the management of people with diabetes. *Journal of Advanced Nursing* 2015;**71**(12):2950–2964. https://doi.org/10.1111/jan.12757.

38. Petticrew M, Roberts H. Systematic reviews in the social sciences: a practical guide. John Wiley & Sons 2008:79–246. https://doi.org/10.1002/9780470754887.

39. Al Hamarneh YN, Johnston K, Marra CA, et al. Pharmacist prescribing and care improves cardiovascular risk, but is it cost-effective? A cost-effectiveness analysis of the RxEACH study. *Canadian Pharmacists Journal* 2019;**152**(4):257–266. https://doi.org/10.1177/1715163519851822.

40. Hale A, Merlo G, Nissen L, et al. Cost-effectiveness analysis of doctor-pharmacist collaborative prescribing for venous thromboembolism in high risk surgical patients. *BMC Health Services Research* 2018;**18**(1):749. https://doi.org/10.1186/s12913-018-3557-0.

41. Marra C, Johnston K, Santschi V, et al. Cost-effectiveness of pharmacist care for managing hypertension in Canada. *Canadian Pharmacists Journal* 2017;**150**(3):184–197. https://doi.org/10.1177/1715163517701109.

42. Neilson AR, Bruhn H, Bond CM, et al. Pharmacist-led management of chronic pain in primary care: costs and benefits in a pilot randomised controlled trial. *BMJ Open* 2015;**5**(4):e006874. https://doi.org/10.1136/bmjopen-2014-006874.

43. Norman IJ, Coster S, McCrone P, et al. A comparison of the clinical effectiveness and costs of mental health nurse supplementary prescribing and independent medical prescribing: a post-test control group study. *BMC Health Services Research* 2010;**10**:4. https://doi.org/10.1186/1472-6963-10-4.

44. i5 Health. Non-medical prescribing (NMP): An economic evaluation. National Health System: Health Education North West 2015. http://www.i5health.com/NMP/NMPEconomicEvaluation.pdf.

45. Tsuyuki RT, Al Hamarneh YN, Jones CA, et al. The Effectiveness of Pharmacist Interventions on Cardiovascular Risk: The Multicenter Randomized Controlled RxEACH Trial. *Journal of American College of Cardiology* 2016;**67**(24):2846–2854. https://doi.org/10.1016/j.jacc.2016.03.528.

46. Hale AR, Coombes ID, Stokes J, et al. Perioperative medication management: expanding the role of the preadmission clinic pharmacist in a single centre, randomised controlled trial of collaborative prescribing. *BMJ Open* 2013;**3**(7):e003027. https://doi.org/10.1136/bmjopen-2013-003027.

47. Tsuyuki RT, Houle SK, Charrois TL, et al. Randomized Trial of the Effect of Pharmacist Prescribing on Improving Blood Pressure in the Community: The Alberta Clinical Trial in Optimizing Hypertension (RxACTION). *Circulation* 2015;**132**(2):93–100. https://doi.org/10.1161/CIRCULATIONAHA.115.015464.

48. Edwards J, Coward M, Carey N. Barriers and facilitators to implementation of non-medical independent prescribing in primary care in the UK: a qualitative systematic review. BMJ Open 2022;**12**(6):e052227. https://doi.org/10.1136/ bmjopen-2021-052227.

For peer review only

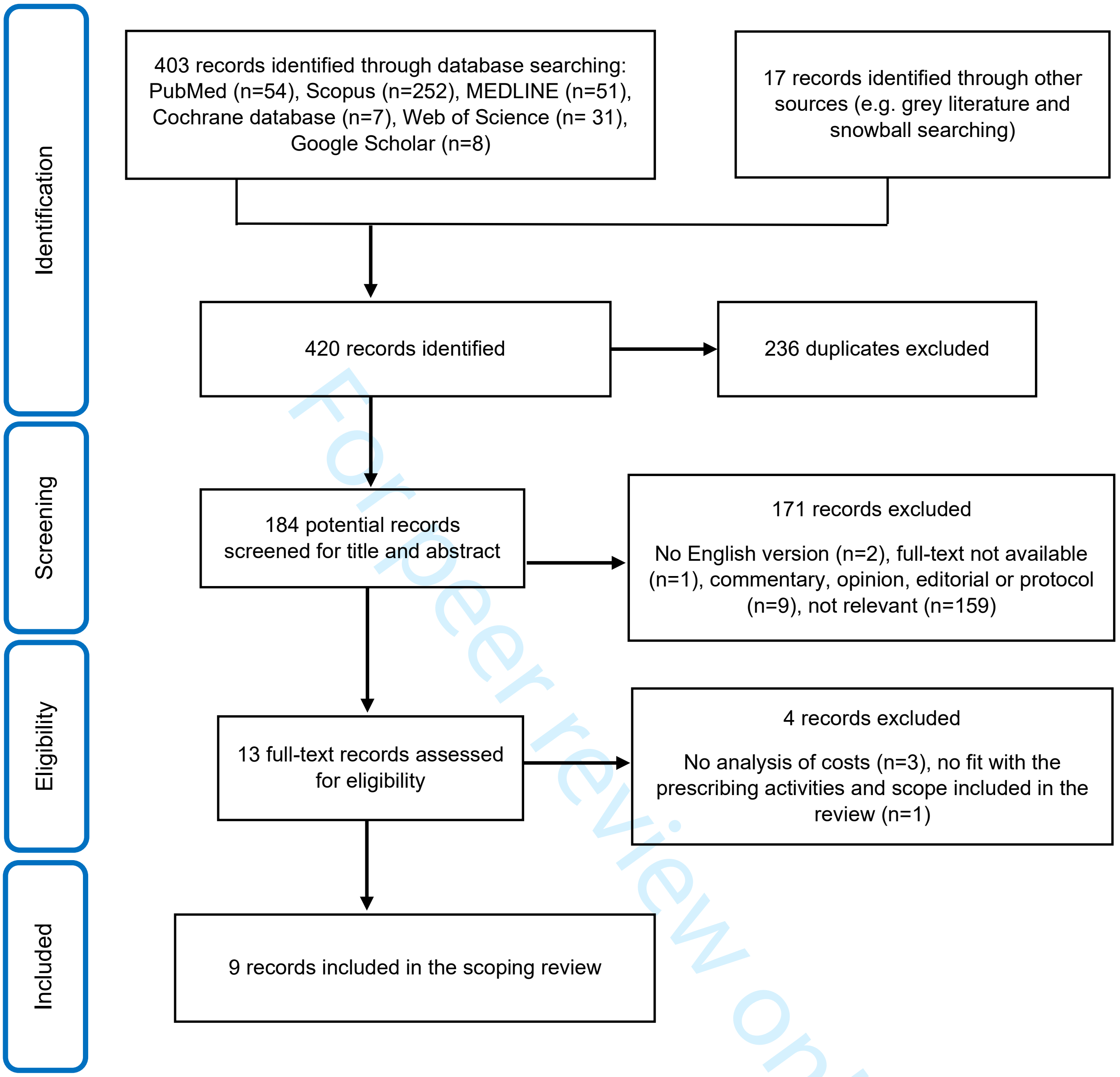

Figure 1. PRISMA flowchart of the study selection process

Costs, consequences and value for money in non-medical prescribing: A scoping review

Supplementary Data

Table A.1 Search terms used in PubMed from 1 January 1999 to 1 January 2022

**Database**: PubMed		
**Date of search**: 14 January 2022		
**Search step**	**Search terms and queries**	**Results**
S1	"non-medical prescribing" OR "non-medical prescriber" OR "non-medical prescribers" OR "non-doctor prescribing" OR "non-doctor prescriber" OR "non-doctor prescribers" [All Fields]	216
S2	"non-medical prescribing" OR "non-medical prescriber" OR "non-medical prescribers" OR "non-doctor prescribing" OR "non-doctor prescriber" OR "non-doctor prescribers" [All Fields] Filters: from 1999/1/1 - 2022/1/1	215
S3	"medicine management" AND activities [All Fields] Filters: from 1999/1/1 - 2022/1/1	47
S4	S2 OR S3	262
S5	(outcome OR consequence OR effectiveness OR impact) AND (economic OR cost) [All Fields] Filters: from 1999/1/1 - 2022/1/1	555,862
S6	S4 AND S5	28
S7	"allied health profession" OR "allied health professions" OR "allied health professional" OR "allied health professionals" OR "non-medical profession" OR "non-medical professions" OR “non-medical professional" OR "non-medical professionals" OR nurse OR pharmacist OR dietitian OR dietician OR radiographer OR podiatrist OR physiotherapist OR podiatrist OR optometrist OR paramedic OR midwife [All Fields] Filters: from 1999/1/1 - 2022/1/1	563,041
S8	S6 AND S7	26

Table A.2 Search terms used in MEDLINE from 1 January 1999 to 1 January 2022

**Database**: MEDLINE		
**Date of search**: 14 January 2022		
**Step**	**Search terms and queries**	**Results**
S1	"non-medical prescribing" OR "non-medical prescriber" OR "non-medical prescribers" OR "non-doctor prescribing" OR "non-doctor prescriber" OR "non-doctor prescribers" OR "medicine management activities" in Anywhere	212
S2	"non-medical prescribing" OR "non-medical prescriber" OR "non-medical prescribers" OR "medicine management activities" in Anywhere Narrowed by: Entered date: 1999-01-01 to 2022-01-01	211

For peer review only

S3	(outcome OR consequence OR effectiveness OR impact) AND (economic OR cost) in Anywhere	356,562
S4	S2 AND S3	26
S5	"allied health profession" OR "allied health professions" OR "allied health professional" OR "allied health professionals" OR "non-medical profession" OR "non-medical professions" OR “non-medical professional" OR "non-medical professionals" OR nurse OR pharmacist OR dietitian OR radiographer OR podiatrist OR physiotherapist OR podiatrist OR optometrist OR paramedic OR midwife in Anywhere	591,003
S6	S5 AND S4	25

Table A.3 Search terms used in Scopus from 1 January 1999 to 1 January 2022

**Database**: Scopus		
**Date of search**: 14 January 2022		
**Step**	**Search terms and queries**	**Results**
S1	ALL ("non-medical prescribing" OR "non-medical prescriber" OR "non-medical prescribers" OR "non-doctor prescribing" OR "non-doctor prescriber" OR "non-doctor prescribers") AND PUBYEAR > 1998	830
S2	ALL ("medicine management activities") AND PUBYEAR > 1998	11
S3	S1 OR S2	835
S4	ALL ((outcome OR consequence OR effectiveness OR impact) AND (economic OR cost)) AND PUBYEAR > 1998	4,456,222
S5	S3 AND S4	266
S6	ALL ("allied health profession" OR "allied health professions" OR "allied health professional" OR "allied health professionals" OR "non-medical profession" OR "non-medical professions" OR "non-medical professional" OR "non-medical professionals" OR nurse OR pharmacist OR dietitian OR radiographer OR podiatrist OR physiotherapist OR podiatrist OR optometrist OR paramedic OR midwife) AND PUBYEAR > 1998	1,091,538
S7	S5 AND S6	252

Table A.4 Search terms used in Web of Science from 1 January 1999 to 1 January 2022

**Database**: Web of Science		
**Date of search**: 14 January 2022		
**Step**	**Search terms and queries**	**Results**
S1	ALL=("non-medical prescribing" OR "non-medical prescriber" OR "non-medical prescribers" OR "non-doctor prescribing" OR "non-doctor prescriber" OR "non-doctor prescribers" OR "medicine management activities") Timespan: 1999-01-01 to 2022-01-01 (Publication date)	186
S2	ALL=(cost or economic) AND ALL=(outcome OR consequence OR impact OR effectiveness)	825,022
S3	S1 AND S2	17
S4	ALL=("allied health profession" OR "allied health professions" OR "allied health professional" OR "allied health professionals" OR "non-medical	407,152

For peer review only

For peer review only - http://bmjopen.bmj.com/site/about/guidelines.xhtml

	profession" OR "non-medical professions" OR “non-medical professional" OR "non-medical professionals" OR dietitian OR dietician OR radiographer OR therapist OR podiatrist OR physiotherapist OR paramedic OR optometrist OR midwife OR nurse OR pharmacist) Timespan: 1999-01-01 to 2022-01-01 (Publication date)	
S5	S3 AND S4	14

Table A.5 Search terms used in Cochrane Database of Systematic Reviews from 1 January 1999 to 1 January 2022

**Database**: Cochrane Database of Systematic Reviews		
**Date of search**: 14 January 2022		
**Step**	**Search terms and queries**	**Results**
S1	"non-medical prescribing" OR "non-medical prescriber" OR "non-medical prescribers" OR "non-doctor prescribing" OR "non-doctor prescriber" OR "medicine management activities" in All Texts	7
S2	(outcome OR effectiveness OR consequence OR impact) AND (cost or economic) in All Texts	5,675
S3	S1 AND S2	7

For peer review only - http://bmjopen.bmj.com/site/about/guidelines.xhtml
For peer review only

Costs, consequences and value for money in non-medical prescribing: A scoping review

Table B. Preferred Reporting Items for Systematic reviews and Meta-Analyses extension for Scoping Reviews (PRISMA-ScR) Checklist

SECTION	ITEM	PRISMA-ScR CHECKLIST ITEM	REPORTED ON PAGE #
**TITLE**			
Title	1	Identify the report as a scoping review.	1
**ABSTRACT**			
Structured summary	2	Provide a structured summary that includes (as applicable): background, objectives, eligibility criteria, sources of evidence, charting methods, results, and conclusions that relate to the review questions and objectives.	2
**INTRODUCTION**			
Rationale	3	Describe the rationale for the review in the context of what is already known. Explain why the review questions/objectives lend themselves to a scoping review approach.	5
Objectives	4	Provide an explicit statement of the questions and objectives being addressed with reference to their key elements (e.g., population or participants, concepts, and context) or other relevant key elements used to conceptualise the review questions and/or objectives.	4, 5-8
**METHODS**			
Protocol and registration	5	Indicate whether a review protocol exists; state if and where it can be accessed (e.g., a Web address); and if available, provide registration information, including the registration number.	6
Eligibility criteria	6	Specify characteristics of the sources of evidence used as eligibility criteria (e.g., years considered, language, and publication status), and provide a rationale.	7, 8
Information sources*	7	Describe all information sources in the search (e.g., databases with dates of coverage and contact with authors to identify additional sources), as well as the date the most recent search was executed.	7, 8
Search	8	Present the full electronic search strategy for at least 1 database, including any limits used, such that it could be repeated.	7, Tables A.1 to A.5 in Supplementary Data
Selection of sources of evidence†	9	State the process for selecting sources of evidence (i.e., screening and eligibility) included in the scoping review.	8, 9
Data charting process‡	10	Describe the methods of charting data from the included sources of evidence (e.g., calibrated forms or forms that have been tested by the team before their use, and whether data charting was done independently or in duplicate) and any processes for obtaining and confirming data from investigators.	8, 9
Data items	11	List and define all variables for which data were sought and any assumptions and simplifications made.	5, Table 1
Critical appraisal of individual sources of evidence§	12	If done, provide a rationale for conducting a critical appraisal of included sources of evidence; describe the methods used and how this information was used in any data synthesis (if appropriate).	NA
Synthesis of results	13	Describe the methods of handling and summarising the data that were charted.	9
**RESULTS**			
Selection of sources of evidence	14	Give numbers of sources of evidence screened, assessed for eligibility, and included in the review, with reasons for exclusions at each stage, ideally using a flow diagram.	10

For peer review only - http://bmjopen.bmj.com/site/about/guidelines.xhtml

SECTION	ITEM	PRISMA-ScR CHECKLIST ITEM	REPORTED ON PAGE #
Characteristics of sources of evidence	15	For each source of evidence, present characteristics for which data were charted and provide the citations.	10, Table 3 and Table C in Supplementary Data
Critical appraisal within sources of evidence	16	If done, present data on critical appraisal of included sources of evidence (see item 12).	NA
Results of individual sources of evidence	17	For each included source of evidence, present the relevant data that were charted that relate to the review questions and objectives.	10-20, Table 3 and Table C in Supplementary Data
Synthesis of results	18	Summarise and/or present the charting results as they relate to the review questions and objectives.	10-20, Table 3 and Table C in Supplementary Data
**DISCUSSION**			
Summary of evidence	19	Summarise the main results (including an overview of concepts, themes, and types of evidence available), link to the review questions and objectives, and consider the relevance to key groups.	20, 21
Limitations	20	Discuss the limitations of the scoping review process.	22, 23
Conclusions	21	Provide a general interpretation of the results with respect to the review questions and objectives, as well as potential implications and/or next steps.	23
**FUNDING**			
Funding	22	Describe sources of funding for the included sources of evidence, as well as sources of funding for the scoping review. Describe the role of the funders of the scoping review.	24

JBI = Joanna Briggs Institute; PRISMA-ScR = Preferred Reporting Items for Systematic reviews and Meta-Analyses extension for Scoping Reviews; NA = Not Available.
* Where *sources of evidence* (see second footnote) are compiled from, such as bibliographic databases, social media platforms, and Web sites.
† A more inclusive/heterogeneous term used to account for the different types of evidence or data sources (e.g., quantitative and/or qualitative research, expert opinion, and policy documents) that may be eligible in a scoping review as opposed to only studies. This is not to be confused with *information sources* (see first footnote).
‡ The frameworks by Arksey and O'Malley and Levac and colleagues and the JBI guidance refer to the process of data extraction in a scoping review as data charting.
§ The process of systematically examining research evidence to assess its validity, results, and relevance before using it to inform a decision. This term is used for items 12 and 19 instead of "risk of bias" (which is more applicable to systematic reviews of interventions) to include and acknowledge the various sources of evidence that may be used in a scoping review (e.g., quantitative and/or qualitative research, expert opinion, and policy document).

Reference: Tricco AC, Lillie E, Zarin W, O'Brien KK, Colquhoun H, Levac D, et al. PRISMA Extension for Scoping Reviews (PRISMAScR): Checklist and Explanation. Ann Intern Med. 2018;169:467–473. http://doi.org/10.7326/M18-0850.

For peer review only - http://bmjopen.bmj.com/site/about/guidelines.xhtml

Table C: Cost and outcome measures and key findings of included studies

Authors (year)	Type of prescribing	Outcome measures	Cost and resource use measures	Perspective	Study main findings
Black et al. (2022)[7]	Nurse IP	Patient consultation experience; patient satisfaction with information about medicines; medication appropriateness; medication effectiveness for the condition using the number of unplanned re-consultations in 3 months	From the NHS perspective: NMP course training and governance (staff supervision, study time and backfill); clinic processes (including medication provision, error, appropriateness, consultation duration, impact on the workload of other professionals, rates of unplanned re-consultations).<br>From nurses' perspective: study leave; personal time to study; out-of-pocket expenses for travel and purchase of learning resources for training; prospect promotions.	UK NHS, nurses and patients	***Consequences***<br>- Patient satisfaction: over 96% in both groups.<br>- Medication decisions safety: 96% for nurse prescribers vs 99% for PGD users.<br>- Medication errors: minor for both nurse prescribers (56%) and PGD users (62%), mainly documentation-related (78%), with no patients harmed.<br>- Consultation duration and unplanned re-consultations: similar for both groups. Nurse prescribers sought assistance from colleagues less frequently but provided longer consultations.<br>***Costs***<br>- NMP training fees: fully paid by their employers or health grants (£900 to £3,555) in 2016.<br>- Study days: An average of 20.1 employer-paid study days were reported by 92% of nurses, and an average of 7.4 clinically supervised days, for each nurse (an average cost of £6,45 to the NHS per nurse) during training. Eighty-one percent of nurse prescribers spent an average of 26.3 days of personal time studying for their NMP qualifications.<br>- PGD-related costs to the employer: £912 for creating a new PGD and £276 for updating.<br>- Medication costs: the average costs per patient were higher for the nurse prescriber group (£19) than for PGD users (£11.25).<br>- Nurse prescribers were in higher pay bands.
Carey et al. (2020)[21]	Physiotherapist IP<br>Podiatrist IP	Patient satisfaction; ease of access to services; quality of life	Rates of relevant tests or services ordered; frequency of new medicines; referrals to other health professionals; frequency of follow-up; consultations numbers and durations; time spent discussing the patient with other colleagues; unplanned consultations for the same condition within two months of the index consultation.	NA	***Consequences***<br>- Acceptability of independent prescribing: high (77%), with 23% preferring a GP to prescribe.<br>- Quality of life and patient satisfaction with services: significant improvements between baseline and follow-up with no significant differences in both groups.<br>***Costs***<br>- Consultation duration: An average of 6.8 minutes higher for physiotherapist prescribers and 3.5 minutes for podiatrist prescribers compared to non-prescribers.<br>- The prescriber podiatrists more frequently ordered medications and tests than non-prescribing colleagues. No cost was calculated and reported.<br>- Follow-up consultations: no significant differences in both groups.<br>- Cost of consultations: An average of £7.95 for physiotherapist prescribers and £8.62 for podiatrist prescribers higher than non-prescriber groups.<br>- No NMP training-related costs (training course, travel, supervision time, etc) were reported.

For peer review only

For peer review only - http://bmjopen.bmj.com/site/about/guidelines.xhtml

Authors (year)	Type of prescribing	Outcome measures	Cost and resource use measures	Perspective	Study main findings
Al Hamarneh et al. (2019)[39]	Pharmacist IP	QALYs; life years and CVD risk	Cost of baseline and follow-up visits, cost of half-day training and direct medical costs (e.g. inpatient, outpatient, medication, etc)	Canadian Medicare	***Consequences***<br>- Life years: increased by 0.11 per patient; QALYs: increased by 0.19; risk of CVD: decreased by 0.10.<br>***Costs***<br>- Medical costs: decreased by CA$2,149 compared to usual care.<br>- Cost of pharmacist intervention – 1st year: CA$233 [baseline visit: CA$125 (baseline); CA$25 (per follow-up) and cost of half-day training: CA$500 (CA$33.3 per patient)].<br>- Cost of pharmacist intervention – 2nd year: CA$175.<br>***Cost-effectiveness***<br>- Pharmacist prescribing was estimated to save more than CA$4.4 billion, add 576,689 QALYs and prevent more than 8.9 million CVD events over 30 years if applied to only 15% of the eligible patients in Canada.
Hale et al. (2018)[40]	Pharmacist IP	QALYs	Annual costs of existing and new pharmacy services; time taken by the pharmacist to prescribe (minus time saved for medical prescriber); annual salary of pharmacist (minus time costs offset by medical prescribers not having to prescribe); direct costs of acute DVT and PE to the healthcare system	Australian healthcare sector	***Consequences***<br>- QALYs: increased by 0.02 per patient in the pharmacist prescriber group.<br>- The proportion of treated VTE patients: 100% in the pharmacist prescriber and 91% in the GP groups.<br>***Costs***<br>- Cost of introducing the pharmacist IP: AU$2.24 per patient (calculated based on annual pharmacist salary and overhead costs minus GP time not required to prescribe).<br>***Cost-effectiveness***<br>- The probability of pharmacist prescribing and care being cost-effective at a willingness-to-pay of $AU40,000 was 95%.
Marra et al. (2017)[41]	Pharmacist IP	Reduced systolic blood pressure in patients with hypertension; relative risk of CVD; relative risk of renal disease; hazard ratio of mortality after CVD; life years; and HRQoL	Direct medical costs of the health conditions and the costs associated with implementing the pharmacist intervention strategy and training.	Canadian Medicare	***Consequences***<br>- Blood pressure and CVD cases: reduced by 0.21 per patient in the pharmacist prescribing group<br>- Life years: increased by 0.3 in the pharmacist prescribing group<br>- QALYs: increased by 0.4 in the pharmacist prescribing group<br>***Costs***<br>- Cost of pharmacist intervention – 1st year: CA$200 [baseline visit: CA$125 (baseline); CA$25 (per follow-up) and cost of half-day training: CA$500 (CA$33.3 per patient)].<br>- Cost of pharmacist intervention – 2nd year: CA$75.<br>- Cost of pharmacist intervention – 3rd year: CA$50.<br>***Cost-effectiveness***<br>- Pharmacist prescribing was found cost-effective and cost-saving (the reduction in costs associated with CVD and ESRD equated to cost-savings of CA$6,365 per patient in the pharmacist group over 30 years.

For peer review only - http://bmjopen.bmj.com/site/about/guidelines.xhtml

Authors (year)	Type of prescribing	Outcome measures	Cost and resource use measures	Perspective	Study main findings
i5 Health (2015)[44]	IP and SP (for a range of healthcare professions, e.g. physiotherapists, pharmacists and radiographers)<br>Community nurse prescribers	NA	Number of consultations; visits and referrals to medical prescribers avoided; lower A&E attendance and waiting times; avoided readmissions; inappropriate prescribing	NA	***Consequences***<br>- NMP practitioners provided information on patient's medications in 99% of reviews and identified inappropriate medication regimens in 50% of appointments.<br>***Costs***<br>- As shown by the i5 Health analysis of the audits for different non-medical prescriber groups in primary and secondary care in England, the main savings of NMP practices included 32% of GP appointments and 31% of follow-up appointments by a consultant.<br>- The annual value contributed by adding one new non-medical prescriber was over £270 million. The total cost-savings were predicted to be approximately £800 million in England in 2014.<br>- No NMP training-related costs (training course, travel, supervision time, etc) were reported.
Courtenay et al. (2015)[37]	Nurse IP	Patient-reported diabetes self-care activities; HbA1c test results; patients' satisfaction with activities, process and medicine management	Employment of cost; consultation cost; advice-seeking from other professionals and GP prescribing signing; prescribing costs; use of other health services	UK NHS	***Consequences***<br>- Patient satisfaction: in the nurse prescriber groups, patients were more satisfied with some specific aspects of care. However, there were no significant differences in general and overall satisfaction.<br>- Diabetes self-care activities: no significant differences were found.<br>- HbA1c test result: The HbA1c score decreased significantly in both groups over six months.<br>- Frequency of consultations: no significant differences were found.<br>***Costs***<br>- Consultation duration: longer consultations (average, 7.7 minutes) were provided by nurse prescribers for an additional cost of £6.<br>- New prescriptions and the use of healthcare services: no significant differences were reported regarding by patients in both groups.<br>- Most prescribing nurses were on a higher salary band than non-prescribing colleagues.
Neilson et al. (2015)[42]	Pharmacist IP	QALYs; chronic pain grade; anxiety and depression scale	Pharmacist training, pharmacist and GP time to deliver the intervention and provide follow-up; the number of hospitalisation days and outpatient visits); primary care visits for chronic pain (GP, nurse, healthcare assistant); telephone contacts for chronic pain; prescribed and non-prescribed OTC pain-related medications and health service resource use	UK NHS	***Consequences***<br>- QALYs: very small in both prescribing and review groups (relative to usual care, the mean differences in QALYs were 0.0069 for prescribing and 0.0097 for review groups, respectively)<br>***Costs***<br>- The average cost differences per patient were £77 for prescribing and £54 for review groups relative to usual care.<br>Overall, based on the expected value of sample information analysis the authors suggested a larger sample size (e.g. the optimal sample size was estimated at 780 for prescribing and 540 for the review group using a cost per QALY threshold of £20,000) for reliable findings.

For peer review only - http://bmjopen.bmj.com/site/about/guidelines.xhtml

Authors (year)	Type of prescribing	Outcome measures	Cost and resource use measures	Perspective	Study main findings
Norman et al. (2010)[43]	Nurse SP	Medication adherence; health status; adverse effects; patient satisfaction (with information, consultation, treatment, etc), patient's perception of improvement in their health problem; social functioning and impairment; depression scale	Training costs (time off work and other expenses, e.g. travel, supervision time, etc); costs of prescribing (e.g. time spent preparing for a prescribing, time taken to prescribe for a patient and number of patients prescribed for); patients' use of healthcare; cost of service per patient	Health and social care	***Consequences***<br>- Medicine adherence, health status, side effects, and satisfaction with overall care: no significant differences were reported between patients across the nurse and medical prescriber groups.<br>***Costs***<br>- Psychiatric inpatient costs were an average of £1,186 significantly higher per patient for the nurse prescriber group than those in the medical prescriber group.<br>- Total annual costs per patient: no significant differences were reported.<br>- The prescribing training course fee: £497 per patient.<br>- No additional training-related costs (travel, supervision time, etc) were reported.

Note. NMP: non-medical prescribing; PGD: patient group direction; IP: independent prescribing; SP: supplementary prescribing; CVD: cardiovascular disease; HRQoL: health-related quality of life; NHS: National Health Service; HbA1c: this refers to average blood sugar test. OTC: over the counter; QALYs: quality-adjusted life years. QALY is a generic metric used to value and quantify health outcomes in terms of both the quality and the quantity of life lived.[26]; ESRD: end-stage renal disease; VTE: venous thromboembolism, which manifests as either deep vein thrombosis (DVT) or pulmonary embolism (PE); GP: general practitioners; NA: not available.

For peer review only - http://bmjopen.bmj.com/site/about/guidelines.xhtml
